# Supplementary material for: Adaptation of Helicoverpa armigera to Soybean Peptidase Inhibitors Is Associated with the Transgenerational Upregulation of Serine Peptidases
Source: Int J Mol Sci. 2022 Nov 18;23(22):14301. doi: 10.3390/ijms232214301 (PMC9693090; doi:10.3390/ijms232214301)
Supplement: Supplementary file 1 [file ijms-23-14301-s001.zip › ijms-1995534-supplementary figures.pdf]

International Journal of
Molecular Sciences

MDPI

Supplementary Figures

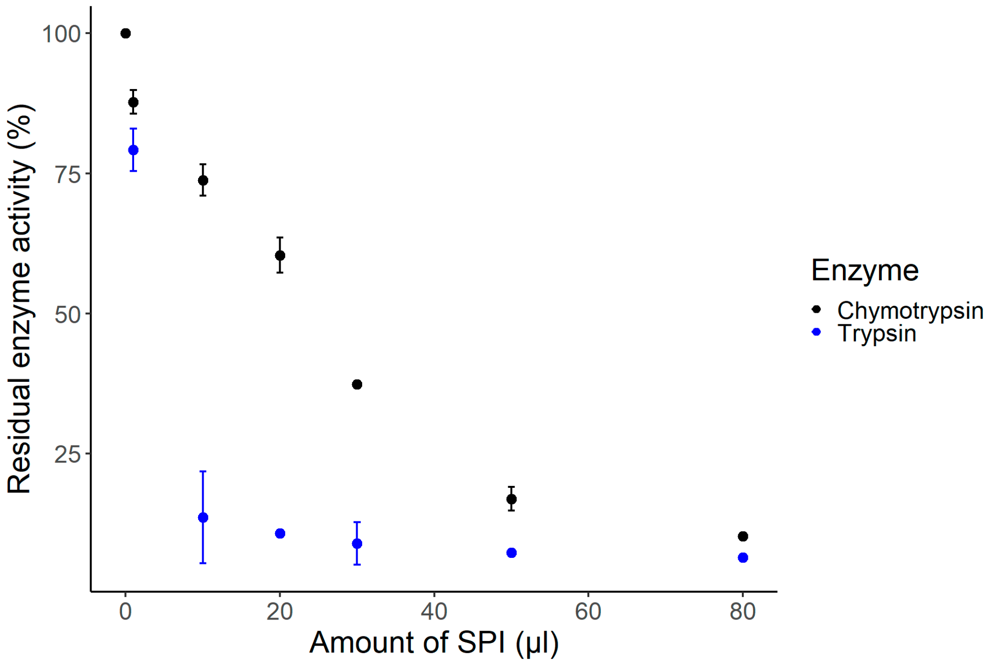

Figure S1. Inhibitory activity of soybean protein extracts against trypsins (blue) and chymotrypsins (black). Error bars represent standard error from three replicates.

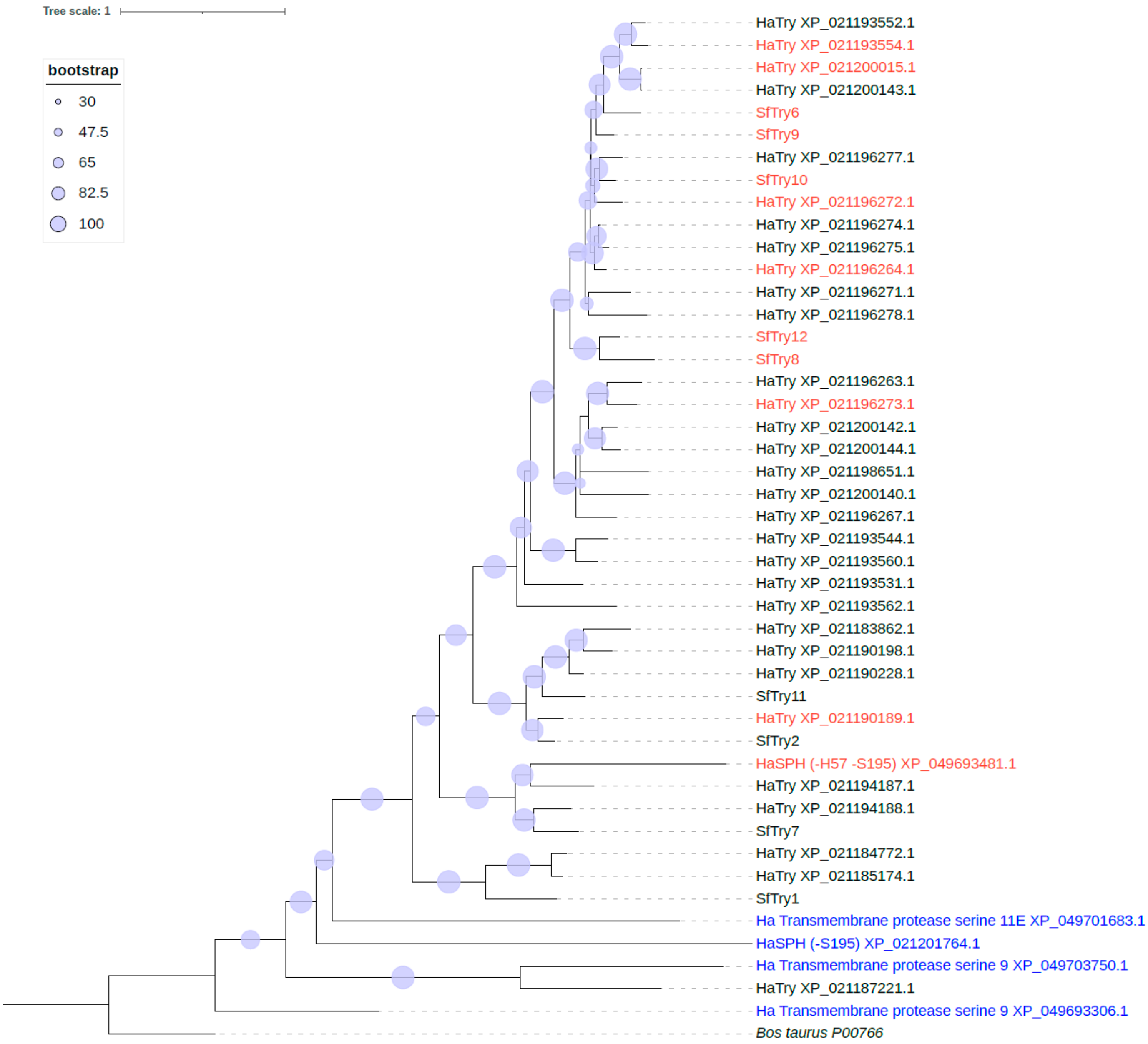

Figure S2. Phylogenetic analysis of amino acid sequences of the *S. frugiperda* (SfTry) and *H. armigera* (HaTry) unresponsive and responsive trypsin, serine peptidase homologs (SPHs) and transmembrane protease serine genes. SPHs did not present the complete catalytic triad (H57, D102 and S195). The phylogenetic tree was constructed using the neighbor-joining method. The bootstrap values were calculated on 1000 replicates.

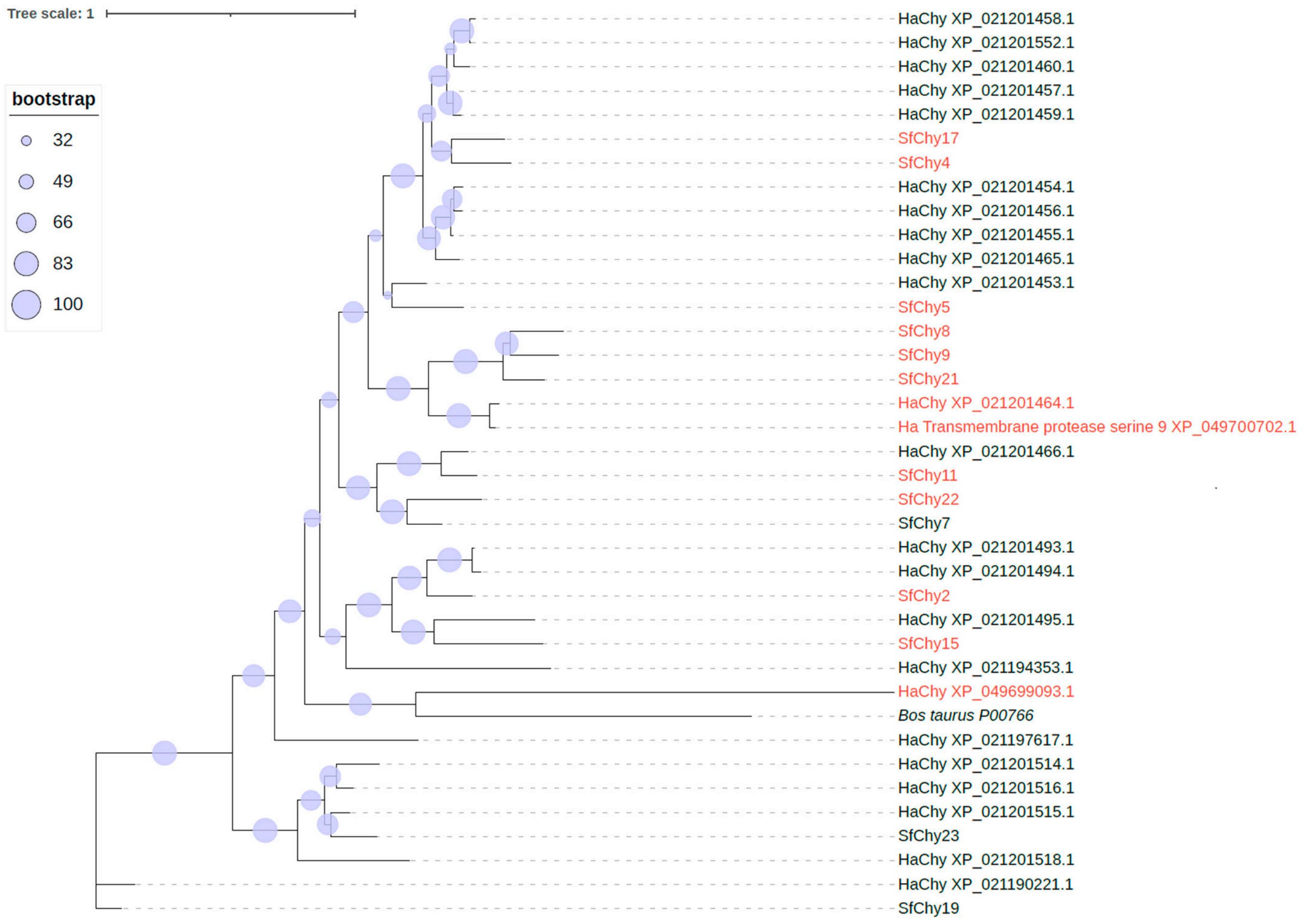

Figure S3. Phylogenetic analysis of amino acid sequences of the *S. frugiperda* (SfChy) and *H. armigera* (HaChy) unresponsive and responsive trypsin and transmembrane protease serine genes. SPHs did not present the complete catalytic triad (H57, D102 and S195). The phylogenetic tree was constructed using the neighbor-joining method. The bootstrap values were calculated on 1000 replicates.

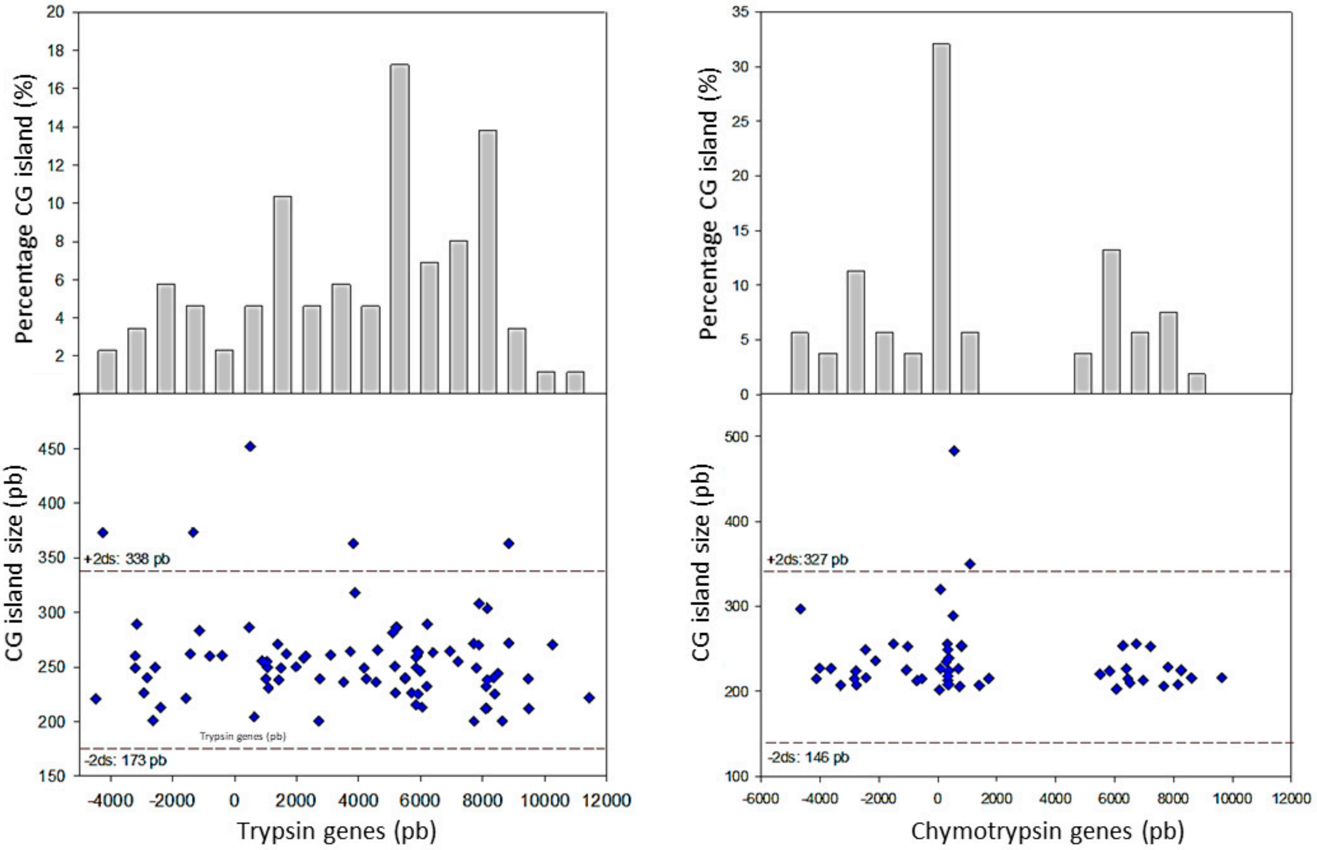

Figure S4. Distribution of CG islands (CGIs) for *H. armigera* trypsin and chymotrypsin genes. Mapping of CGIs was performed along the gene body and the 5 kb regulatory regions upstream and downstream. CGIs were predicted using the CPGPLOT program (EMBOSS).

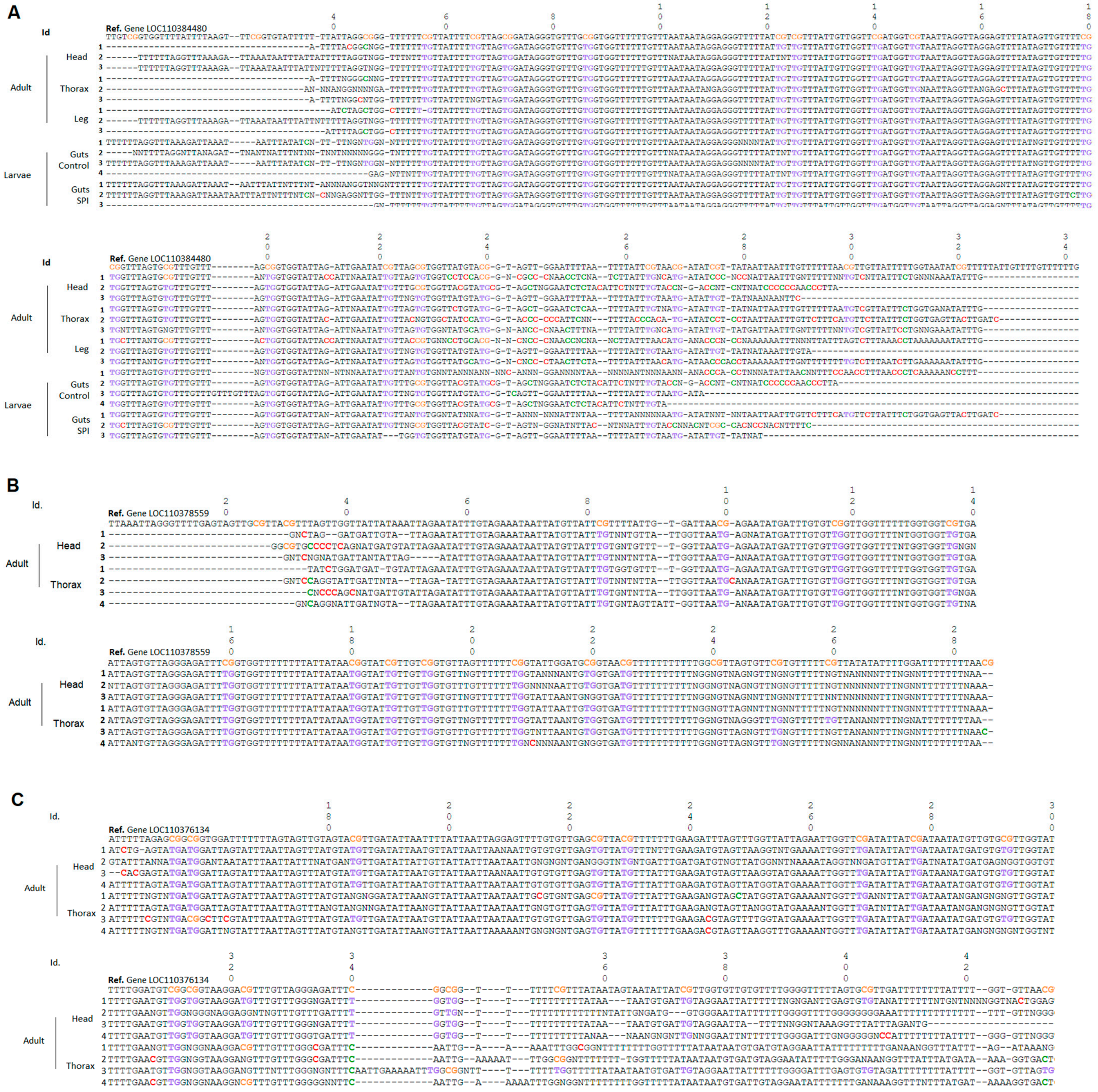

Figure S5. Results of Bisulfite Sequence PCR (BSP) analysis of trypsin genes. Tissue samples were obtained from the digestive system of *H. armigera* larvae fed a diet supplemented with and without SPI (A) and tissues from the head, thorax and legs (A, B, C). The results were consistent with those obtained using whole-genome bisulfite sequencing (WGBS). The CG islands are almost completely hypomethylated in the coding region in both the digestive system and adult tissues.

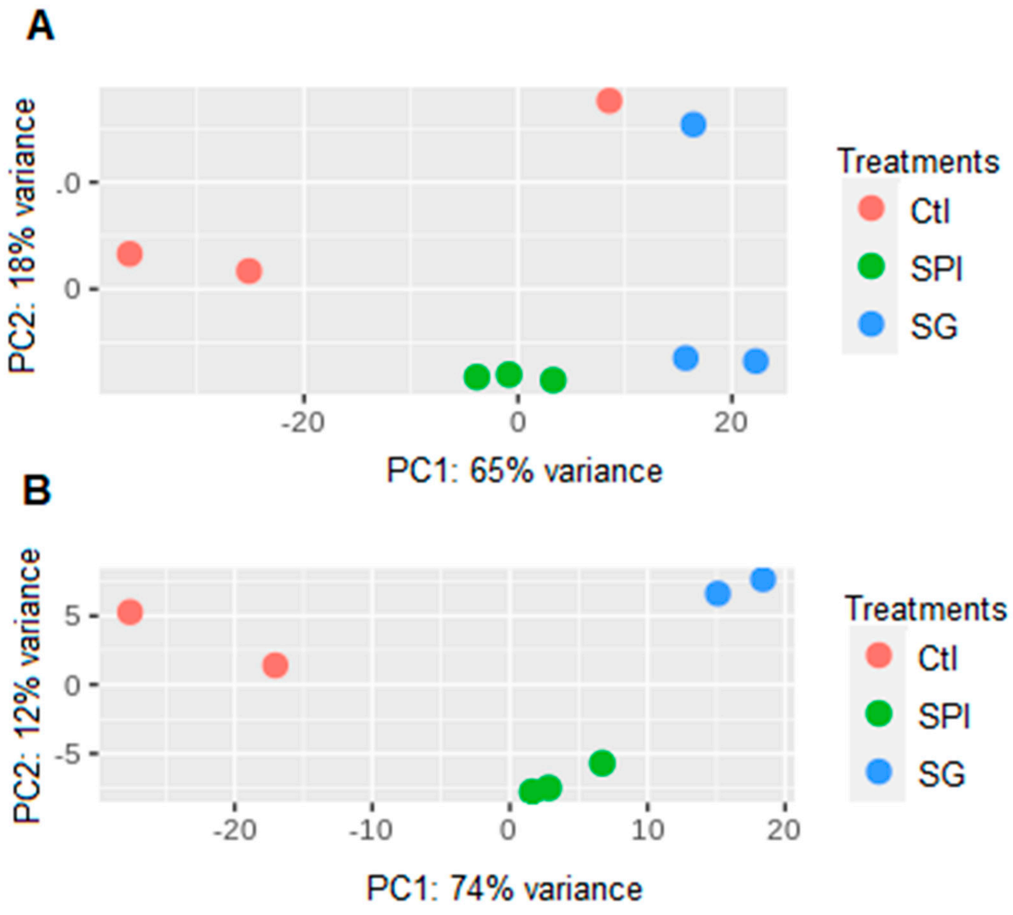

Figure S6. Principal component analysis of RNA-seq data derived from nine experimental treatments. First generation larvae fed on artificial diet (Ctl), on artificial diet supplemented with PIs (IPS), progeny of larvae fed on artificial diet supplemented with PIs (SG) (A). The two main components explain 86% of the total variance (B).
