# Supplementary material for: Adaptation of Helicoverpa armigera to Soybean Peptidase Inhibitors Is Associated with the Transgenerational Upregulation of Serine Peptidases
Source: Int J Mol Sci. 2022 Nov 18;23(22):14301. doi: 10.3390/ijms232214301 (PMC9693090; doi:10.3390/ijms232214301)
Supplement: Supplementary file 1 [file ijms-23-14301-s001.zip › ijms-1995534-supplementary tables.pdf]

Supplementary Tables

Table S1. Differentially expressed genes in *H. armigera* larvae exposed to SPI.

Locus	Protein	SPI vs. CTL Log2 Fold-Change	(*p-adjust*<0.05)	SG vs. CTL Log2 Fold-Change	(*p-adjust*<0.05)	SG vs. SPI Log2 Fold-Change	(*p-adjust*<0.05)
LOC110371322	craniofacial development protein 2-like	NA	NA	-7.070436957	2.82E-59	NA	NA
LOC110378338	Fanconi anemia group I protein-like	-1.791640797	7.18E-09	-4.251071867	2.87E-34	NA	NA
LOC110382545	uncharacterized protein LOC110382545	-2.776694574	2.72E-09	-5.952436796	9.04E-29	NA	NA
LOC110371485	uncharacterized protein LOC110371485	NA	NA	3.043802104	2.05E-27	NA	NA
LOC110378414	mucin-2-like	-4.816802708	7.02E-28	-6.128282836	2.05E-27	NA	NA
LOC110376175	uncharacterized protein LOC110376175	-2.80169976	2.98E-09	-5.706842781	1.79E-25	NA	NA
LOC110380962	uncharacterized protein LOC110380962 isoform X2	-2.585618097	1.89E-11	-5.055150463	8.08E-25	NA	NA
LOC110380962	uncharacterized protein LOC110380962 isoform X1	-2.585618097	1.89E-11	-5.055150463	8.08E-25	NA	NA
LOC110371042	myosin-2 heavy chain-like isoform X1	-3.086495998	1.40E-11	-5.500475426	2.00E-24	NA	NA
LOC110371042	myosin-2 heavy chain-like isoform X2	-3.086495998	1.40E-11	-5.500475426	2.00E-24	NA	NA

LOC110381109	LOW QUALITY PROTEIN: uncharacterized protein LOC110381109, partial	-2.600367654	8.56E-09	-5.052862274	3.45E-24	NA	NA
LOC110376926	uncharacterized protein LOC110376926	-2.716950034	1.18E-11	-5.162609947	1.39E-23	NA	NA
LOC110373825	uncharacterized protein LOC110373825 isoform X1	-2.955437688	3.86E-10	-6.578996959	2.03E-23	NA	NA
LOC110373825	uncharacterized protein LOC110373825 isoform X2	-2.955437688	3.86E-10	-6.578996959	2.03E-23	NA	NA
LOC110373825	uncharacterized protein LOC110373825 isoform X3	-2.955437688	3.86E-10	-6.578996959	2.03E-23	NA	NA
LOC110378201	proton-coupled amino acid transporter-like protein CG1139	-2.436275894	2.88E-10	-6.799703961	1.29E-22	NA	NA
LOC110373449	uncharacterized protein LOC110373449 isoform X2	-2.882338445	3.66E-14	-4.391013864	1.29E-22	NA	NA
LOC110373449	uncharacterized protein LOC110373449 isoform X3	-2.882338445	3.66E-14	-4.391013864	1.29E-22	NA	NA
LOC110373449	uncharacterized protein LOC110373449 isoform X1	-2.882338445	3.66E-14	-4.391013864	1.29E-22	NA	NA
LOC110369643	uncharacterized protein LOC110369643 isoform X2	-2.559660133	3.05E-10	-4.578849366	3.57E-22	NA	NA

LOC110369643	uncharacterized protein LOC110369643 isoform X1	-2.559660133	3.05E-10	-4.578849366	3.57E-22	NA	NA
LOC110384673	titin-like	-3.109117908	1.67E-11	-5.565106463	5.61E-22	NA	NA
LOC110379210	paramyosin-like	-2.850235276	4.06E-11	-4.918885836	7.30E-22	NA	NA
LOC110369613	uncharacterized protein LOC110369613	-3.172077248	3.94E-13	-5.560161239	5.20E-21	NA	NA
LOC110376913	uncharacterized protein LOC110376913	-2.77404049	2.98E-09	-4.925374651	9.61E-21	NA	NA
LOC110377986	NA	NA	NA	3.274861428	3.48E-20	NA	NA
LOC110381954	NADP-dependent malic enzyme-like	-3.398327044	4.03E-13	-5.7546632	4.10E-20	NA	NA
LOC110382743	keratin-associated protein 10-4-like	-2.647651022	1.55E-08	-6.080902784	1.28E-19	NA	NA
LOC110379119	63 kDa chaperonin, mitochondrial	-2.943293763	6.93E-11	-6.118361017	1.90E-19	NA	NA
LOC110382220	uncharacterized protein LOC110382220 isoform X1	-2.460733868	4.11E-09	-4.289733238	2.07E-19	NA	NA
LOC110382220	uncharacterized protein LOC110382220 isoform X3	-2.460733868	4.11E-09	-4.289733238	2.07E-19	NA	NA
LOC110382220	uncharacterized protein LOC110382220 isoform X2	-2.460733868	4.11E-09	-4.289733238	2.07E-19	NA	NA
LOC110383737	uncharacterized protein LOC110383737	2.480535729	0.004552097	6.415140033	2.18E-19	NA	NA
LOC110377976	uncharacterized protein LOC110377976	-3.063099525	6.57E-10	-6.353291917	3.76E-19	NA	NA

LOC110380328	apoptosis-inducing factor 1, mitochondrial-like	-1.963933693	1.82E-08	-3.335925898	7.86E-19	NA	NA
LOC110382225	uncharacterized protein LOC110382225 isoform X2	-3.114515861	8.91E-11	-6.910346401	2.45E-18	NA	NA
LOC110382225	uncharacterized protein LOC110382225 isoform X1	-3.114515861	8.91E-11	-6.910346401	2.45E-18	NA	NA
LOC110382225	uncharacterized protein LOC110382225 isoform X3	-3.114515861	8.91E-11	-6.910346401	2.45E-18	NA	NA
LOC110373892	uncharacterized protein LOC110373892 isoform X3	-2.566527042	2.42E-08	-5.773192752	3.43E-18	NA	NA
LOC110373892	uncharacterized protein LOC110373892 isoform X2	-2.566527042	2.42E-08	-5.773192752	3.43E-18	NA	NA
LOC110373892	proteoglycan 4-like isoform X1	-2.566527042	2.42E-08	-5.773192752	3.43E-18	NA	NA
LOC110373892	uncharacterized protein LOC110373892 isoform X4	-2.566527042	2.42E-08	-5.773192752	3.43E-18	NA	NA
LOC110378119	uncharacterized protein LOC110378119	-2.967038037	2.16E-11	-6.614896643	6.65E-18	NA	NA
LOC110381629	uncharacterized protein LOC110381629	-3.058866756	5.44E-10	-5.248242203	1.10E-17	NA	NA
LOC110371804	high mobility group protein B1-like isoform X2	NA	NA	-2.408117686	2.12E-17	NA	NA

LOC110371804	high mobility group protein B3-like isoform X1	NA	NA	-2.408117686	2.12E-17	NA	NA
LOC110370601	uncharacterized protein LOC110370601	-2.148541298	2.05E-05	-5.938894694	4.60E-17	NA	NA
LOC110381137	uncharacterized protein LOC110381137 isoform X1	-3.071137244	1.71E-09	-5.525201282	5.16E-17	NA	NA
LOC110381137	uncharacterized protein LOC110381137 isoform X2	-3.071137244	1.71E-09	-5.525201282	5.16E-17	NA	NA
LOC110377275	uncharacterized protein DDB_G0284459-like isoform X2	-2.245328568	1.01E-06	-4.559704901	6.06E-17	NA	NA
LOC110377275	uncharacterized protein DDB_G0284459-like isoform X1	-2.245328568	1.01E-06	-4.559704901	6.06E-17	NA	NA
LOC110377275	uncharacterized protein DDB_G0284459-like isoform X3	-2.245328568	1.01E-06	-4.559704901	6.06E-17	NA	NA
LOC110379359	uncharacterized protein LOC110379359	-2.865352965	3.80E-10	-6.415756984	1.32E-16	NA	NA
LOC110377447	solute carrier family 13 member 5-like isoform X3	-2.347171258	3.38E-05	-6.379326617	1.57E-16	NA	NA
LOC110377447	solute carrier family 13 member 5-like isoform X2	-2.347171258	3.38E-05	-6.379326617	1.57E-16	NA	NA

LOC110377447	solute carrier family 13 member 5-like isoform X1	-2.347171258	3.38E-05	-6.379326617	1.57E-16	NA	NA
LOC110373242	uncharacterized protein LOC110373242 isoform X2	-3.012854331	3.05E-09	-5.911764284	1.71E-16	NA	NA
LOC110373242	uncharacterized protein LOC110373242 isoform X1	-3.012854331	3.05E-09	-5.911764284	1.71E-16	NA	NA
LOC110374621	putative sulfiredoxin	-2.268784562	3.38E-12	-2.830940083	1.81E-16	NA	NA
LOC110369700	uncharacterized protein LOC110369700 isoform X2	-2.675096755	1.23E-07	-5.301801023	5.55E-16	NA	NA
LOC110369700	uncharacterized protein LOC110369700 isoform X1	-2.675096755	1.23E-07	-5.301801023	5.55E-16	NA	NA
LOC110373034	ADP/ATP translocase 1-like	-2.615219155	2.95E-07	-6.233124931	6.12E-16	NA	NA
LOC110374272	LOW QUALITY PROTEIN: sugar transporter ERD6-like 4	-1.45172201	0.003383335	-3.900354086	1.08E-15	NA	NA
LOC110378563	uncharacterized protein LOC110378563	-2.654289688	3.44E-08	-4.84219662	1.71E-15	NA	NA
LOC110380305	probable sulfite oxidase, mitochondrial isoform X2	NA	NA	-1.518049345	1.88E-15	NA	NA
LOC110380305	probable sulfite oxidase, mitochondrial isoform X3	NA	NA	-1.518049345	1.88E-15	NA	NA
LOC110380305	probable sulfite oxidase, mitochondrial isoform X1	NA	NA	-1.518049345	1.88E-15	NA	NA

LOC110381094	uncharacterized protein LOC110381094	-3.07507178	5.12E-08	-6.046304375	2.04E-15	NA	NA
LOC110377177	NA	-4.794686633	6.86E-18	-5.173190826	4.13E-15	NA	NA
LOC110373463	uncharacterized protein LOC110373463	-2.550086482	3.86E-08	-5.802799034	4.49E-15	NA	NA
LOC110375807	uncharacterized protein LOC110375807	NA	NA	5.986785807	5.04E-15	NA	NA
LOC110382327	protamine-like isoform X3	-2.430979754	2.09E-06	-6.007864506	5.99E-15	NA	NA
LOC110382327	protamine-2-like isoform X4	-2.430979754	2.09E-06	-6.007864506	5.99E-15	NA	NA
LOC110382327	protamine-like isoform X1	-2.430979754	2.09E-06	-6.007864506	5.99E-15	NA	NA
LOC110382327	protamine-like isoform X2	-2.430979754	2.09E-06	-6.007864506	5.99E-15	NA	NA
LOC110373271	succinate dehydrogenase	-2.949611003	4.37E-13	-7.706568626	6.67E-15	NA	NA
LOC110374870	uncharacterized protein LOC110374870	-2.901179151	3.20E-08	-6.327933604	1.14E-14	NA	NA
LOC110375099	mitochondrial pyruvate carrier 1-like	-2.731863721	1.39E-10	-6.788687891	1.71E-14	NA	NA
LOC110369603	uncharacterized protein LOC110369603	-2.769475477	1.24E-07	-4.601805915	1.92E-14	NA	NA
LOC110377093	long-chain fatty acid transport protein 1	-1.733097521	1.37E-20	-1.559535077	2.12E-14	NA	NA
LOC110374062	uncharacterized protein LOC110374062	-2.992852529	2.70E-08	-5.252563304	3.31E-14	NA	NA

LOC110373251	mitochondrial 2-oxoglutarate/malate carrier protein-like	-3.095635976	3.38E-12	-7.634081449	3.39E-14	NA	NA
LOC110381130	uncharacterized protein LOC110381130	-2.01368117	2.38E-05	-5.916118433	3.75E-14	NA	NA
LOC110382131	dihydrolipoyllysine-residue succinyltransferase component of 2-oxoglutarate dehydrogenase complex, mitochondrial-like	-2.649238077	9.84E-06	-6.046038919	4.63E-14	NA	NA
LOC110382814	kallikrein-7-like	-2.721899323	3.46E-06	-6.170281243	5.86E-14	NA	NA
LOC110384502	uncharacterized protein LOC110384502	-2.635768601	1.85E-09	-3.932111628	6.39E-14	NA	NA
LOC110384517	probable cytochrome P450 301a1, mitochondrial	-2.587896245	1.30E-06	-4.919381617	6.67E-14	NA	NA
LOC110371748	major heat shock 70 kDa protein Ab-like	-2.659793905	2.15E-08	-6.825604098	6.82E-14	NA	NA
LOC110378137	centrosomal protein of 290 kDa-like	-1.832322271	5.37E-06	-3.298722784	7.46E-14	NA	NA
LOC110371947	WW domain-containing oxidoreductase-like isoform X1	-2.919322193	8.80E-09	-5.136113144	1.20E-13	NA	NA
LOC110371947	WW domain-containing oxidoreductase-like isoform X3	-2.919322193	8.80E-09	-5.136113144	1.20E-13	NA	NA

LOC110371947	WW domain-containing oxidoreductase-like isoform X4	-2.919322193	8.80E-09	-5.136113144	1.20E-13	NA	NA
LOC110371947	WW domain-containing oxidoreductase-like isoform X2	-2.919322193	8.80E-09	-5.136113144	1.20E-13	NA	NA
LOC110381192	stathmin-4 isoform X3	-2.46442169	3.28E-27	-1.870502723	1.27E-13	NA	NA
LOC110381192	probable kinetochore protein nuf2 isoform X1	-2.46442169	3.28E-27	-1.870502723	1.27E-13	NA	NA
LOC110381192	probable kinetochore protein nuf2 isoform X2	-2.46442169	3.28E-27	-1.870502723	1.27E-13	NA	NA
LOC110378259	uncharacterized protein LOC110378259	-2.632593905	9.60E-09	-6.68064603	1.30E-13	NA	NA
LOC110373002	otoferlin-like	-3.197866123	2.28E-08	-6.014817512	1.49E-13	NA	NA
LOC110376858	mitochondrial pyruvate carrier 2-like	-2.441729507	2.47E-07	-4.112605173	1.51E-13	NA	NA
LOC110378349	uncharacterized protein LOC110378349 isoform X1	-2.532915232	4.38E-06	-5.871938886	1.51E-13	NA	NA
LOC110378349	uncharacterized protein LOC110378349 isoform X2	-2.532915232	4.38E-06	-5.871938886	1.51E-13	NA	NA
LOC110370522	cytochrome P450 6B6-like	NA	NA	-1.406564442	1.59E-13	NA	NA
LOC110369699	uncharacterized protein LOC110369699	-3.578260824	3.38E-12	-4.518881057	1.59E-13	NA	NA
LOC110383215	myosin-7-like isoform X5	-2.231965799	1.78E-05	-4.634372498	1.92E-13	NA	NA
LOC110383215	myosin-7-like isoform X2	-2.231965799	1.78E-05	-4.634372498	1.92E-13	NA	NA
LOC110383215	myosin-7-like isoform X3	-2.231965799	1.78E-05	-4.634372498	1.92E-13	NA	NA

LOC110383215	myosin-7-like isoform X4	-2.231965799	1.78E-05	-4.634372498	1.92E-13	NA	NA
LOC110383215	myosin-7-like isoform X1	-2.231965799	1.78E-05	-4.634372498	1.92E-13	NA	NA
LOC110377973	uncharacterized protein LOC110377973 isoform X1	-3.067485036	1.24E-07	-5.648193904	2.10E-13	NA	NA
LOC110377973	uncharacterized protein LOC110377973 isoform X2	-3.067485036	1.24E-07	-5.648193904	2.10E-13	NA	NA
LOC110375008	uncharacterized protein LOC110375008	-2.668759824	8.52E-06	-5.008597124	2.57E-13	NA	NA
LOC110381615	UDP-glucuronosyltransferase 2B14-like	NA	NA	-2.851065873	2.99E-13	NA	NA
LOC110376454	uncharacterized protein LOC110376454 isoform X1	-2.682311629	8.66E-07	-6.034368447	3.10E-13	NA	NA
LOC110376454	uncharacterized protein LOC110376454 isoform X2	-2.682311629	8.66E-07	-6.034368447	3.10E-13	NA	NA
LOC110374087	uncharacterized protein LOC110374087	-1.624491952	3.01E-05	-3.330956177	3.11E-13	NA	NA
LOC110373980	uncharacterized protein LOC110373980	-3.178301953	1.31E-08	-6.45038998	3.87E-13	NA	NA
LOC110371816	uncharacterized protein LOC110371816	-2.947701984	2.99E-10	-7.35676634	4.57E-13	NA	NA
LOC110371614	stearoyl-CoA desaturase 5	NA	NA	1.685516387	5.12E-13	NA	NA

LOC110373207	mitochondrial 2-oxoglutarate/malate carrier protein-like isoform X1	-2.530716892	1.23E-06	-5.856954588	5.25E-13	NA	NA
LOC110373207	mitochondrial 2-oxoglutarate/malate carrier protein-like isoform X2	-2.530716892	1.23E-06	-5.856954588	5.25E-13	NA	NA
LOC110381643	uncharacterized protein LOC110381643	-2.861506377	1.08E-07	-5.669303673	5.57E-13	NA	NA
LOC110372872	uncharacterized protein LOC110372872 isoform X2	NA	NA	1.73809456	5.69E-13	NA	NA
LOC110372872	uncharacterized protein LOC110372872 isoform X1	NA	NA	1.73809456	5.69E-13	NA	NA
LOC110373622	uncharacterized protein LOC110373622	NA	NA	-6.457376288	5.81E-13	NA	NA
LOC110373875	uncharacterized protein LOC110373875 isoform X7	-2.596562576	8.95E-06	-4.742782061	5.92E-13	NA	NA
LOC110373875	uncharacterized protein LOC110373875 isoform X1	-2.596562576	8.95E-06	-4.742782061	5.92E-13	NA	NA
LOC110373875	uncharacterized protein LOC110373875 isoform X6	-2.596562576	8.95E-06	-4.742782061	5.92E-13	NA	NA

LOC110373875	uncharacterized protein LOC110373875 isoform X5	-2.596562576	8.95E-06	-4.742782061	5.92E-13	NA	NA
LOC110373875	uncharacterized protein LOC110373875 isoform X4	-2.596562576	8.95E-06	-4.742782061	5.92E-13	NA	NA
LOC110373875	uncharacterized protein LOC110373875 isoform X3	-2.596562576	8.95E-06	-4.742782061	5.92E-13	NA	NA
LOC110373875	uncharacterized protein LOC110373875 isoform X2	-2.596562576	8.95E-06	-4.742782061	5.92E-13	NA	NA
LOC110373875	uncharacterized protein LOC110373875 isoform X8	-2.596562576	8.95E-06	-4.742782061	5.92E-13	NA	NA
LOC110375962	tektin-3-like	-2.681151607	1.42E-07	-4.959361209	7.67E-13	NA	NA
LOC110375170	mono	-2.645817042	3.20E-08	-5.569233557	1.15E-12	NA	NA
LOC110372316	uncharacterized protein LOC110372316	-3.128607504	8.87E-09	-6.219684748	1.27E-12	NA	NA
LOC110370282	uncharacterized protein LOC110370282	-2.656218547	4.57E-08	-4.325816012	1.54E-12	NA	NA
LOC110380264	peroxisomal N(1)-acetyl-spermine/spermidine oxidase-like isoform X2	NA	NA	-1.453921083	1.59E-12	NA	NA
LOC110380264	peroxisomal N(1)-acetyl-spermine/spermidine oxidase-like isoform X7	NA	NA	-1.453921083	1.59E-12	NA	NA

LOC110380264	peroxisomal N(1)-acetyl-spermine/spermidine oxidase-like isoform X4	NA	NA	-1.453921083	1.59E-12	NA	NA
LOC110380264	peroxisomal N(1)-acetyl-spermine/spermidine oxidase-like isoform X3	NA	NA	-1.453921083	1.59E-12	NA	NA
LOC110380264	peroxisomal N(1)-acetyl-spermine/spermidine oxidase-like isoform X1	NA	NA	-1.453921083	1.59E-12	NA	NA
LOC110380264	peroxisomal N(1)-acetyl-spermine/spermidine oxidase-like isoform X5	NA	NA	-1.453921083	1.59E-12	NA	NA
LOC110380264	peroxisomal N(1)-acetyl-spermine/spermidine oxidase-like isoform X6	NA	NA	-1.453921083	1.59E-12	NA	NA
LOC110373188	uncharacterized protein LOC110373188	-2.399855537	3.71E-09	-3.056246409	1.66E-12	NA	NA
LOC110382756	UDP-glucuronosyltransferase 2B10-like	NA	NA	-2.303251319	1.69E-12	NA	NA
LOC110380426	protein pellino isoform X1	-1.279743098	1.49E-09	-1.567514723	1.80E-12	NA	NA
LOC110380426	protein pellino isoform X2	-1.279743098	1.49E-09	-1.567514723	1.80E-12	NA	NA
LOC110371072	peroxisomal N(1)-acetyl-spermine/spermidine oxidase-like	-2.85337786	1.24E-07	-5.802635132	1.82E-12	NA	NA

LOC110370323	organic cation transporter-like protein	-2.654447599	1.03E-08	-3.785513196	2.22E-12	NA	NA
LOC110378009	katanin p60 ATPase-containing subunit A1-like isoform X1	-2.83674996	3.17E-07	-4.972571193	2.91E-12	NA	NA
LOC110378009	katanin p60 ATPase-containing subunit A1-like isoform X2	-2.83674996	3.17E-07	-4.972571193	2.91E-12	NA	NA
LOC110373500	testis-specific serine/threonine-protein kinase 2	-2.444501927	4.38E-06	-7.302413274	3.03E-12	NA	NA
LOC110374876	uncharacterized protein LOC110374876	-1.799843551	9.12E-05	-5.335327506	3.46E-12	NA	NA
LOC110381761	uncharacterized protein LOC110381761	-2.730345507	1.24E-07	-3.902158431	3.89E-12	NA	NA
LOC110370783	uncharacterized protein LOC110370783	-2.934228162	3.02E-07	-4.486289225	3.89E-12	NA	NA
LOC110372470	uncharacterized protein LOC110372470	-2.899558963	2.24E-07	-4.234030453	4.23E-12	NA	NA
LOC110378185	aldose 1-epimerase-like	-2.612930917	1.52E-06	-5.250057744	4.25E-12	NA	NA
LOC110371281	uncharacterized protein LOC110371281 isoform X1	-2.879046522	2.47E-06	-4.931328644	4.78E-12	NA	NA
LOC110371281	uncharacterized protein LOC110371281 isoform X2	-2.879046522	2.47E-06	-4.931328644	4.78E-12	NA	NA
LOC110371281	uncharacterized protein LOC110371281 isoform X3	-2.879046522	2.47E-06	-4.931328644	4.78E-12	NA	NA

LOC110382578	uncharacterized protein LOC110382578	NA	NA	-2.533903909	5.58E-12	NA	NA
LOC110378813	uncharacterized protein LOC110378813	-2.327434927	5.64E-06	-5.042217306	6.44E-12	NA	NA
LOC110374478	uncharacterized protein LOC110374478	-2.617610922	5.20E-05	-5.453825977	6.44E-12	NA	NA
LOC110374895	uncharacterized protein LOC110374895	-2.963974017	1.09E-08	-5.498118999	8.38E-12	NA	NA
LOC110384496	uncharacterized protein LOC110384496	-2.671537496	9.79E-07	-5.180106655	8.53E-12	NA	NA
LOC110384469	cholinesterase 1-like	1.851320332	0.001320609	3.696631281	9.61E-12	NA	NA
LOC110373410	uncharacterized protein LOC110373410	-2.656556275	1.59E-06	-4.642172282	1.23E-11	NA	NA
LOC110373893	enolase-like	-3.221874478	7.55E-08	-5.124597627	1.76E-11	NA	NA
LOC110377083	uncharacterized protein LOC110377083	-4.641920269	6.71E-22	-3.288940255	1.92E-11	NA	NA
LOC110375026	uncharacterized protein LOC110375026	-2.461407442	2.87E-05	-5.448998325	2.41E-11	NA	NA
LOC110382056	tyrosine-protein phosphatase cdcA-like	-2.85711781	5.08E-06	-5.312666611	3.16E-11	NA	NA
LOC110369641	uncharacterized protein LOC110369641 isoform X1	-3.060450584	1.23E-08	-5.762726261	3.20E-11	NA	NA
LOC110369641	uncharacterized protein LOC110369641 isoform X2	-3.060450584	1.23E-08	-5.762726261	3.20E-11	NA	NA
LOC110375230	traB domain-containing protein-like isoform X1	-2.419217302	1.04E-05	-4.332239905	3.47E-11	NA	NA

LOC110375230	traB domain-containing protein-like isoform X2	-2.419217302	1.04E-05	-4.332239905	3.47E-11	NA	NA
LOC110377338	NA	-2.202823974	0.000572087	-5.805977268	3.73E-11	NA	NA
LOC110378550	uncharacterized protein LOC110378550 isoform X2	-2.615360446	1.78E-05	-5.498585695	3.90E-11	NA	NA
LOC110378550	uncharacterized protein LOC110378550 isoform X3	-2.615360446	1.78E-05	-5.498585695	3.90E-11	NA	NA
LOC110378550	uncharacterized protein LOC110378550 isoform X4	-2.615360446	1.78E-05	-5.498585695	3.90E-11	NA	NA
LOC110378550	uncharacterized protein LOC110378550 isoform X5	-2.615360446	1.78E-05	-5.498585695	3.90E-11	NA	NA
LOC110378550	uncharacterized protein LOC110378550 isoform X1	-2.615360446	1.78E-05	-5.498585695	3.90E-11	NA	NA
LOC110372253	protein yippee-like CG15309 isoform X2	-1.480644985	3.38E-12	-1.493735139	5.06E-11	NA	NA
LOC110372253	protein yippee-like CG15309 isoform X1	-1.480644985	3.38E-12	-1.493735139	5.06E-11	NA	NA
LOC110372253	protein yippee-like CG15309 isoform X3	-1.480644985	3.38E-12	-1.493735139	5.06E-11	NA	NA
LOC110382031	putative uncharacterized protein DDB_G0282133	-2.486841091	5.80E-05	-4.809782354	5.85E-11	NA	NA
LOC110376624	uncharacterized protein LOC110376624	-2.363021638	2.89E-05	-6.24471259	6.72E-11	NA	NA

LOC110384653	uncharacterized protein LOC110384653	-2.438392066	2.01E-06	-4.032621788	1.00E-10	NA	NA
LOC110373327	tektin-B1-like	-3.36401886	9.50E-07	-6.289844853	1.06E-10	NA	NA
LOC110384657	uncharacterized protein LOC110384657	-2.645052046	3.38E-05	-5.198319183	1.10E-10	NA	NA
LOC110374146	pyruvate kinase-like	-2.787297166	1.41E-06	-6.217198299	1.32E-10	NA	NA
LOC110383819	uncharacterized protein LOC110383819	-2.360557027	0.000105465	-4.226586758	1.55E-10	NA	NA
LOC110377912	glutathione S-transferase 1-like	-2.483739061	2.49E-05	-5.449973703	1.70E-10	NA	NA
LOC110381513	tektin-4-like	-2.360738543	7.97E-05	-6.89539732	1.85E-10	NA	NA
LOC110374529	probable serine/threonine-protein kinase kinX	-2.898589658	5.34E-06	-5.280558208	1.89E-10	NA	NA
LOC110371408	ubiquitin carboxyl-terminal hydrolase 7-like isoform X1	-2.436845587	0.000285029	-5.250491966	1.94E-10	NA	NA
LOC110371408	ubiquitin carboxyl-terminal hydrolase 7-like isoform X2	-2.436845587	0.000285029	-5.250491966	1.94E-10	NA	NA
LOC110371408	ubiquitin carboxyl-terminal hydrolase 7-like isoform X3	-2.436845587	0.000285029	-5.250491966	1.94E-10	NA	NA
LOC110379885	uncharacterized protein LOC110379885	NA	NA	6.126745309	2.60E-10	NA	NA
LOC110375085	glutamate dehydrogenase, mitochondrial-like	-2.859744862	0.003286446	-5.986777265	3.09E-10	NA	NA

LOC110370375	uncharacterized protein LOC110370375	-2.669164098	2.41E-06	-5.263786139	3.39E-10	NA	NA
LOC110372655	uncharacterized protein LOC110372655	-2.52657299	6.39E-06	-5.227446658	3.57E-10	NA	NA
LOC110379266	uncharacterized protein LOC110379266 isoform X1	-3.388820199	1.11E-06	-5.377385588	3.85E-10	NA	NA
LOC110379266	uncharacterized protein LOC110379266 isoform X2	-3.388820199	1.11E-06	-5.377385588	3.85E-10	NA	NA
LOC110370610	putative ribosome-binding factor A, mitochondrial	-1.652787262	1.28E-06	-2.243380289	4.25E-10	NA	NA
LOC110376796	thioredoxin domain-containing protein 12-like	-2.513018122	2.10E-06	-5.398922627	4.30E-10	NA	NA
LOC110372889	uncharacterized protein LOC110372889 isoform X1	-2.286006231	1.59E-05	-6.528284853	4.93E-10	NA	NA
LOC110372889	uncharacterized protein LOC110372889 isoform X2	-2.286006231	1.59E-05	-6.528284853	4.93E-10	NA	NA
LOC110379292	facilitated trehalose transporter Tret1-like	-1.416501606	0.00322019	-2.819390184	4.98E-10	NA	NA
LOC110377256	uncharacterized protein LOC110377256 isoform X2	-2.788211697	2.10E-06	-5.077506207	4.98E-10	NA	NA
LOC110377256	uncharacterized protein LOC110377256 isoform X1	-2.788211697	2.10E-06	-5.077506207	4.98E-10	NA	NA

LOC110376139	putative carbonic anhydrase 5	NA	NA	-2.802683117	5.57E-10	NA	NA
LOC110371916	peroxisomal N(1)-acetyl-spermine/spermidine oxidase-like	-2.623953935	9.38E-06	-4.460720141	7.73E-10	NA	NA
LOC110378184	uncharacterized protein LOC110378184	NA	NA	-4.448932676	8.35E-10	NA	NA
LOC110371237	testis-specific serine/threonine-protein kinase 3 isoform X3	-2.573557331	3.36E-05	-5.661787183	8.59E-10	NA	NA
LOC110371237	testis-specific serine/threonine-protein kinase 3 isoform X5	-2.573557331	3.36E-05	-5.661787183	8.59E-10	NA	NA
LOC110371237	testis-specific serine/threonine-protein kinase 3 isoform X4	-2.573557331	3.36E-05	-5.661787183	8.59E-10	NA	NA
LOC110371237	testis-specific serine/threonine-protein kinase 3 isoform X2	-2.573557331	3.36E-05	-5.661787183	8.59E-10	NA	NA
LOC110371237	testis-specific serine/threonine-protein kinase 3 isoform X1	-2.573557331	3.36E-05	-5.661787183	8.59E-10	NA	NA
LOC110371237	testis-specific serine/threonine-protein kinase 3 isoform X6	-2.573557331	3.36E-05	-5.661787183	8.59E-10	NA	NA
LOC110373453	uncharacterized protein LOC110373453	1.130877149	0.004731078	2.257997603	8.86E-10	NA	NA
LOC110374413	uncharacterized protein LOC110374413	-2.684347271	9.30E-07	-5.350240103	9.19E-10	NA	NA

LOC110382413	serine/threonine-protein kinase MARK2-like isoform X2	-2.157379215	1.86E-05	-3.394223895	1.08E-09	NA	NA
LOC110382413	serine/threonine-protein kinase MARK2-like isoform X3	-2.157379215	1.86E-05	-3.394223895	1.08E-09	NA	NA
LOC110382413	serine/threonine-protein kinase MARK2-like isoform X1	-2.157379215	1.86E-05	-3.394223895	1.08E-09	NA	NA
LOC110382460	meiosis-specific nuclear structural protein 1-like isoform X1	-2.340538778	2.17E-06	-3.375612588	1.16E-09	NA	NA
LOC110382460	meiosis-specific nuclear structural protein 1-like isoform X2	-2.340538778	2.17E-06	-3.375612588	1.16E-09	NA	NA
LOC110382460	meiosis-specific nuclear structural protein 1-like isoform X3	-2.340538778	2.17E-06	-3.375612588	1.16E-09	NA	NA
LOC110371220	leucine-rich repeat-containing protein 49	-3.298660005	3.33E-09	-3.924421067	1.25E-09	NA	NA
LOC110376912	uncharacterized protein LOC110376912 isoform X1	-2.429811365	0.000545484	-5.888162294	1.38E-09	NA	NA
LOC110376912	uncharacterized protein LOC110376912 isoform X2	-2.429811365	0.000545484	-5.888162294	1.38E-09	NA	NA
LOC110378930	serine/arginine repetitive matrix protein 1-like isoform X1	-2.597450188	7.41E-07	-6.350581325	1.38E-09	NA	NA

LOC110378930	serine/arginine repetitive matrix protein 1-like isoform X2	-2.597450188	7.41E-07	-6.350581325	1.38E-09	NA	NA
LOC110371214	uncharacterized protein PFB0765w-like isoform X2	-2.039814248	3.05E-09	-2.254312187	1.42E-09	NA	NA
LOC110371214	golgin subfamily B member 1-like isoform X1	-2.039814248	3.05E-09	-2.254312187	1.42E-09	NA	NA
LOC110375317	heat shock protein 70 B2-like	-2.392879568	0.000192588	-4.7352481	1.73E-09	NA	NA
LOC110374104	NA	-2.226337789	4.81E-05	-5.690138731	1.93E-09	NA	NA
LOC110369844	NA	-2.424862048	5.14E-05	-5.871567372	1.93E-09	NA	NA
LOC110378519	uncharacterized protein LOC110378519 isoform X1	-2.487734321	0.000242383	-5.473211016	1.93E-09	NA	NA
LOC110378519	uncharacterized protein LOC110378519 isoform X3	-2.487734321	0.000242383	-5.473211016	1.93E-09	NA	NA
LOC110378519	uncharacterized protein LOC110378519 isoform X5	-2.487734321	0.000242383	-5.473211016	1.93E-09	NA	NA
LOC110378519	uncharacterized protein LOC110378519 isoform X4	-2.487734321	0.000242383	-5.473211016	1.93E-09	NA	NA
LOC110378519	uncharacterized protein LOC110378519 isoform X2	-2.487734321	0.000242383	-5.473211016	1.93E-09	NA	NA

LOC110377084	sperm flagellar protein 1-like	-2.97296555	4.38E-06	-5.654965823	2.05E-09	NA	NA
LOC110380171	uncharacterized protein LOC110380171 isoform X2	-2.334658812	2.85E-07	-3.030667029	2.21E-09	NA	NA
LOC110380171	uncharacterized protein LOC110380171 isoform X1	-2.334658812	2.85E-07	-3.030667029	2.21E-09	NA	NA
LOC110382166	IQ domain-containing protein G-like, partial	-2.842076031	6.44E-07	-4.46008549	2.40E-09	NA	NA
LOC110372657	WAS/WASL-interacting protein family member 3-like	-2.335289544	1.74E-05	-6.308635007	2.41E-09	NA	NA
LOC110383214	cilia- and flagella-associated protein 58-like isoform X1	-2.576264503	4.51E-10	-2.657526605	2.41E-09	NA	NA
LOC110383214	cilia- and flagella-associated protein 58-like isoform X2	-2.576264503	4.51E-10	-2.657526605	2.41E-09	NA	NA
LOC110383214	cilia- and flagella-associated protein 58-like isoform X3	-2.576264503	4.51E-10	-2.657526605	2.41E-09	NA	NA
LOC110380848	MICOS complex subunit Mic60-like	-3.009259277	4.02E-06	-5.642144822	2.50E-09	NA	NA
LOC110370517	spidroin-2-like	-3.051275889	6.66E-08	-4.384595549	2.70E-09	NA	NA
LOC110375579	alanine aminotransferase 1-like isoform X2	-1.463924045	0.000103981	-2.314124063	2.73E-09	NA	NA
LOC110375579	alanine aminotransferase 1-like isoform X4	-1.463924045	0.000103981	-2.314124063	2.73E-09	NA	NA

LOC110375579	alanine aminotransferase 1-like isoform X3	-1.463924045	0.000103981	-2.314124063	2.73E-09	NA	NA
LOC110375579	alanine aminotransferase 1-like isoform X1	-1.463924045	0.000103981	-2.314124063	2.73E-09	NA	NA
LOC110377741	cilia- and flagella-associated protein 44-like	-2.999796829	3.57E-06	-5.31788219	2.75E-09	NA	NA
LOC110382292	stabilizer of axonemal microtubules 2	-1.44723993	1.74E-05	-2.090141116	2.78E-09	NA	NA
LOC110384189	uncharacterized protein LOC110384189 isoform X2	NA	NA	-2.942477473	2.80E-09	NA	NA
LOC110384189	uncharacterized protein LOC110384189 isoform X1	NA	NA	-2.942477473	2.80E-09	NA	NA
LOC110376876	WD repeat-containing protein on Y chromosome-like isoform X1	-2.716309575	1.60E-06	-5.684317204	2.95E-09	NA	NA
LOC110376876	WD repeat-containing protein on Y chromosome-like isoform X2	-2.716309575	1.60E-06	-5.684317204	2.95E-09	NA	NA
LOC110371171	hyaluronidase PH-20-like isoform X1	-3.34608721	2.16E-11	-3.207515823	3.04E-09	NA	NA
LOC110371171	hyaluronidase-like isoform X2	-3.34608721	2.16E-11	-3.207515823	3.04E-09	NA	NA
LOC110376119	uncharacterized protein LOC110376119	NA	NA	2.368236656	3.19E-09	NA	NA

LOC110383615	sideroflexin-1-like	-2.440962891	1.34E-05	-3.728858446	3.58E-09	NA	NA
LOC110381642	serine protease inhibitor 88Ea-like isoform X1	NA	NA	1.417690693	4.01E-09	NA	NA
LOC110381642	serine protease inhibitor 88Ea-like isoform X2	NA	NA	1.417690693	4.01E-09	NA	NA
LOC110377809	myosin-9-like isoform X2	-2.712561642	1.96E-05	-5.890242079	4.01E-09	NA	NA
LOC110377809	myosin-9-like isoform X1	-2.712561642	1.96E-05	-5.890242079	4.01E-09	NA	NA
LOC110373289	glutathione S-transferase 1-like	NA	NA	2.536565338	4.20E-09	NA	NA
LOC110384092	m-AAA protease-interacting protein 1, mitochondrial	-1.927946192	0.000821991	-3.700179	4.33E-09	NA	NA
LOC110376877	pollen-specific leucine-rich repeat extensin-like protein 2 isoform X1	-2.730348714	1.34E-05	-5.149035631	4.44E-09	NA	NA
LOC110376877	keratin-associated protein 10-6-like isoform X2	-2.730348714	1.34E-05	-5.149035631	4.44E-09	NA	NA
LOC110377775	mitochondrial import receptor subunit TOM40 homolog	-3.113077888	1.81E-06	-5.241368251	4.98E-09	NA	NA
LOC110377313	LOW QUALITY PROTEIN: uncharacterized protein LOC110377313	-2.598611854	9.16E-06	-5.665343888	5.11E-09	NA	NA
LOC110379313	protein lethal(2)essential for life-like	-2.254229971	0.000242383	-4.547486825	5.17E-09	NA	NA
LOC110383040	serine protease snake-like	NA	NA	-4.938338525	5.92E-09	NA	NA

LOC110381372	uncharacterized protein LOC110381372	-2.314113517	0.000598658	-5.255873441	6.14E-09	NA	NA
LOC110369728	uncharacterized protein LOC110369728	-2.811304785	6.67E-07	-5.521951422	7.26E-09	NA	NA
LOC110374415	TBC1 domain family member 19 isoform X2	-1.628764909	0.003274863	-3.65846593	7.34E-09	NA	NA
LOC110374415	TBC1 domain family member 19 isoform X1	-1.628764909	0.003274863	-3.65846593	7.34E-09	NA	NA
LOC110374415	TBC1 domain family member 19 isoform X3	-1.628764909	0.003274863	-3.65846593	7.34E-09	NA	NA
LOC110375605	uncharacterized protein LOC110375605	-1.584165943	0.000811964	-2.778193357	7.35E-09	NA	NA
LOC110382785	uncharacterized protein LOC110382785	-2.570416204	2.38E-05	-4.398713432	7.35E-09	NA	NA
LOC110374512	uncharacterized protein LOC110374512	-2.802670325	5.51E-09	-7.088252671	7.35E-09	NA	NA
LOC110371098	tubulin glycylase 3A-like isoform X2	-2.552499638	2.58E-05	-4.969473881	8.10E-09	NA	NA
LOC110371098	tubulin glycylase 3A-like isoform X3	-2.552499638	2.58E-05	-4.969473881	8.10E-09	NA	NA
LOC110371098	tubulin glycylase 3A-like isoform X4	-2.552499638	2.58E-05	-4.969473881	8.10E-09	NA	NA
LOC110371098	tubulin glycylase 3A-like isoform X1	-2.552499638	2.58E-05	-4.969473881	8.10E-09	NA	NA
LOC110373473	uncharacterized protein LOC110373473	-2.573711798	6.50E-06	-6.171392373	8.51E-09	NA	NA

LOC110372740	cAMP and cAMP-inhibited cGMP 3\\',5\\'-cyclic phosphodiesterase 10A-like	-2.07152641	0.002266562	-5.438415379	9.24E-09	NA	NA
LOC110377915	uncharacterized protein LOC110377915	-2.344711335	0.000276698	-4.727812045	9.44E-09	NA	NA
LOC110370789	uncharacterized protein KIAA1841 homolog	-2.003852886	0.000168023	-3.452858554	9.50E-09	NA	NA
LOC110374828	uncharacterized protein LOC110374828	-2.767704346	3.77E-05	-4.740128793	1.15E-08	NA	NA
LOC110378232	teneurin-a isoform X5	-2.261646805	3.00E-07	-2.745189015	1.30E-08	NA	NA
LOC110378232	teneurin-a isoform X3	-2.261646805	3.00E-07	-2.745189015	1.30E-08	NA	NA
LOC110378232	teneurin-a isoform X2	-2.261646805	3.00E-07	-2.745189015	1.30E-08	NA	NA
LOC110378232	teneurin-a isoform X6	-2.261646805	3.00E-07	-2.745189015	1.30E-08	NA	NA
LOC110378232	teneurin-a isoform X4	-2.261646805	3.00E-07	-2.745189015	1.30E-08	NA	NA
LOC110378232	teneurin-a isoform X1	-2.261646805	3.00E-07	-2.745189015	1.30E-08	NA	NA
LOC110382845	uncharacterized protein LOC110382845	-2.901727323	1.21E-06	-6.170240322	1.32E-08	NA	NA
LOC110370672	aminoacylase-1-like	NA	NA	-1.383598269	1.33E-08	NA	NA
LOC110374751	malate dehydrogenase, mitochondrial-like	-2.309722067	1.78E-05	-3.623315412	1.38E-08	NA	NA
LOC110382959	sialin-like	-2.887109468	1.87E-05	-6.407273436	1.47E-08	NA	NA
LOC110381512	tektin-4-like	-3.248261049	6.05E-07	-5.297335698	1.53E-08	NA	NA
LOC110371958	uncharacterized protein LOC110371958 isoform X1	-3.630892236	2.24E-08	-4.491983544	1.53E-08	NA	NA

LOC110371958	uncharacterized protein LOC110371958 isoform X2	-3.630892236	2.24E-08	-4.491983544	1.53E-08	NA	NA
LOC110378680	O-acyltransferase like protein-like	NA	NA	-5.313274926	1.57E-08	NA	NA
LOC110383213	zinc transporter 1-like	NA	NA	-1.07654527	1.96E-08	NA	NA
LOC110374129	uncharacterized protein LOC110374129	-2.063293701	0.000416365	-3.814036941	2.01E-08	NA	NA
LOC110374722	coiled-coil domain-containing protein 181-like	-2.687575712	2.05E-05	-6.198587543	2.05E-08	NA	NA
LOC110384193	glutamine synthetase 2 cytoplasmic-like	-2.902474388	2.53E-07	-7.083602488	2.08E-08	NA	NA
LOC110377216	acidic amino acid decarboxylase GADL1	NA	NA	-1.840685626	2.11E-08	NA	NA
LOC110376050	dynein assembly factor 1, axonemal homolog	-2.367699779	0.00029683	-4.020927586	2.23E-08	NA	NA
LOC110378317	coiled-coil domain-containing protein 40	-2.434499465	2.71E-05	-7.091439458	2.38E-08	NA	NA
LOC110374506	succinate--CoA ligase	-3.05573835	1.11E-06	-4.156501662	2.80E-08	NA	NA
LOC110370568	diuretic hormone 1 isoform X3	1.034336587	0.002016224	1.762531211	2.83E-08	NA	NA
LOC110370568	diuretic hormone 1 isoform X2	1.034336587	0.002016224	1.762531211	2.83E-08	NA	NA
LOC110370568	diuretic hormone 45 isoform X1	1.034336587	0.002016224	1.762531211	2.83E-08	NA	NA
LOC110376297	cholinesterase 1-like	NA	NA	-1.380283297	3.25E-08	NA	NA
LOC110373247	cyclin-dependent kinase-like 1	-2.531142739	0.000129783	-4.425582887	3.51E-08	NA	NA

LOC110383732	uncharacterized protein LOC110383732 isoform X3	-1.372608916	0.001514806	-2.49881807	3.83E-08	NA	NA
LOC110383732	uncharacterized protein LOC110383732 isoform X1	-1.372608916	0.001514806	-2.49881807	3.83E-08	NA	NA
LOC110383732	uncharacterized protein LOC110383732 isoform X2	-1.372608916	0.001514806	-2.49881807	3.83E-08	NA	NA
LOC110380587	trypsin, alkaline C-like	NA	NA	1.350737156	3.86E-08	NA	NA
LOC110375792	protein KIAA0556-like	-2.22264192	0.0006597	-4.684596772	4.01E-08	NA	NA
LOC110376868	alpha-(1,3)-fucosyltransferase C-like	-2.651841646	0.000450928	-5.896884083	4.20E-08	NA	NA
LOC110378278	probable beta-hexosaminidase fdl	-2.855884351	6.00E-05	-5.76101149	4.20E-08	NA	NA
LOC110375852	zinc finger protein 555-like	-2.85646846	1.79E-06	-5.949047133	4.22E-08	NA	NA
LOC110378422	uncharacterized protein LOC110378422	-2.527650039	5.44E-05	-6.041252265	4.23E-08	NA	NA
LOC110374092	NA	-2.307886272	0.04978224	-5.595268428	4.26E-08	NA	NA
LOC110372665	NA	-2.482636883	0.000123822	-5.121014891	4.50E-08	NA	NA
LOC110377461	uncharacterized protein LOC110377461	-2.428381155	0.000244107	-4.368289856	4.77E-08	NA	NA
LOC110373290	ubiquitin-like protein 3	-1.409180864	1.93E-09	-1.381451787	4.79E-08	NA	NA
LOC110376974	transmembrane protein 231	-1.496854875	0.007821621	-3.402656893	4.79E-08	NA	NA
LOC110375674	sterol O-acyltransferase 2	NA	NA	-1.462822423	5.02E-08	NA	NA

LOC110383500	ATP synthase subunit beta, mitochondrial-like	-3.146567264	4.07E-05	-5.189492988	5.24E-08	NA	NA
LOC110372851	uncharacterized protein LOC110372851	-2.026110644	0.003565124	-4.92813292	5.49E-08	NA	NA
LOC110370816	uncharacterized protein LOC110370816	-2.267398777	3.96E-05	-6.831986658	5.79E-08	NA	NA
LOC110375054	leucine-rich repeat-containing protein 71-like	-2.770761242	4.59E-06	-6.956523551	6.18E-08	NA	NA
LOC110370125	zinc finger protein 14-like	NA	NA	-1.17940177	7.76E-08	NA	NA
LOC110376767	uncharacterized protein LOC110376767	-2.774995497	3.38E-05	-6.054406466	7.96E-08	NA	NA
LOC110379684	adenylate kinase isoenzyme 1-like	-2.338321904	0.001707113	-4.910638984	9.64E-08	NA	NA
LOC110379804	uncharacterized protein LOC110379804	-2.652492188	0.000259583	-4.876144603	9.76E-08	NA	NA
LOC110379249	decaprenyl-diphosphate synthase subunit 2-like	-2.389573472	0.000197247	-6.923956317	1.04E-07	NA	NA
LOC110378217	uncharacterized protein LOC110378217	-2.616535002	0.00010108	-6.005397321	1.05E-07	NA	NA
LOC110379730	uncharacterized protein LOC110379730	-3.23422452	4.70E-08	-6.785949034	1.08E-07	NA	NA
LOC110381211	uncharacterized protein LOC110381211	-2.792495757	6.11E-05	-5.14739488	1.23E-07	NA	NA
LOC110379987	IQ and AAA domain-containing protein 1-like	-3.096359965	4.74E-08	-6.696011961	1.24E-07	NA	NA
LOC110373377	uncharacterized protein LOC110373377	-2.21313234	0.001351158	-5.076294414	1.26E-07	NA	NA

LOC110379853	cilia- and flagella-associated protein 157	-2.418923012	0.000327654	-5.960634457	1.26E-07	NA	NA
LOC110376887	WD repeat-containing protein on Y chromosome-like	-2.752768433	4.81E-05	-5.06301361	1.26E-07	NA	NA
LOC110380588	trypsin, alkaline C-like	NA	NA	-1.624041222	1.29E-07	NA	NA
LOC110372548	uncharacterized protein LOC110372548 isoform X2	-1.945469753	1.29E-06	-2.264235925	1.30E-07	NA	NA
LOC110372548	uncharacterized protein LOC110372548 isoform X1	-1.945469753	1.29E-06	-2.264235925	1.30E-07	NA	NA
LOC110377979	neural/ectodermal development factor IMP-L2-like	NA	NA	2.556705981	1.35E-07	NA	NA
LOC110372981	uncharacterized protein LOC110372981	-2.977622523	5.62E-05	-5.294028008	1.36E-07	NA	NA
LOC110375052	uncharacterized protein LOC110375052	-2.792043081	3.64E-05	-6.953393402	1.42E-07	NA	NA
LOC110376464	uncharacterized protein LOC110376464 isoform X3	-2.535744977	9.11E-06	-6.686441407	1.43E-07	NA	NA
LOC110376464	uncharacterized protein LOC110376464 isoform X6	-2.535744977	9.11E-06	-6.686441407	1.43E-07	NA	NA
LOC110376464	nucleolar complex protein 2 homolog isoform X5	-2.535744977	9.11E-06	-6.686441407	1.43E-07	NA	NA

LOC110376464	uncharacterized protein LOC110376464 isoform X2	-2.535744977	9.11E-06	-6.686441407	1.43E-07	NA	NA
LOC110376464	uncharacterized protein LOC110376464 isoform X1	-2.535744977	9.11E-06	-6.686441407	1.43E-07	NA	NA
LOC110376464	uncharacterized protein LOC110376464 isoform X4	-2.535744977	9.11E-06	-6.686441407	1.43E-07	NA	NA
LOC110379315	uncharacterized protein LOC110379315	-2.687380696	4.48E-05	-5.888187856	1.51E-07	NA	NA
LOC110383071	putative serine/threonine-protein kinase C05D10.2	-2.355566653	1.41E-06	-2.797959639	1.53E-07	NA	NA
LOC110372948	uncharacterized protein LOC110372948	-2.720668308	0.000145257	-4.629686209	1.57E-07	NA	NA
LOC110378657	uncharacterized protein LOC110378657 isoform X2	-2.007580618	0.00396424	-4.757928605	1.58E-07	NA	NA
LOC110378657	uncharacterized protein LOC110378657 isoform X4	-2.007580618	0.00396424	-4.757928605	1.58E-07	NA	NA
LOC110378657	uncharacterized protein LOC110378657 isoform X1	-2.007580618	0.00396424	-4.757928605	1.58E-07	NA	NA
LOC110378657	uncharacterized protein LOC110378657 isoform X3	-2.007580618	0.00396424	-4.757928605	1.58E-07	NA	NA
LOC110377590	probable tubulin polyglutamylase TTLL1	-2.693404957	2.58E-05	-9.097676963	1.66E-07	NA	NA

LOC110376703	uncharacterized protein LOC110376703	-2.796652623	1.59E-05	-5.250218819	1.69E-07	NA	NA
LOC110372788	septin-2 isoform X1	NA	NA	-3.225696465	1.73E-07	NA	NA
LOC110372788	septin-2 isoform X2	NA	NA	-3.225696465	1.73E-07	NA	NA
LOC110375188	uncharacterized protein LOC110375188	-2.4699082	0.000759617	-5.184692607	1.77E-07	NA	NA
LOC110378389	uncharacterized protein LOC110378389	-2.367314738	0.001483212	-5.542642423	1.87E-07	NA	NA
LOC110382192	uncharacterized protein LOC110382192	-2.605045354	0.000106883	-5.879390807	1.89E-07	NA	NA
LOC110382145	uncharacterized protein LOC110382145	-2.519891186	0.000128349	-5.821343869	2.02E-07	NA	NA
LOC110381514	tektin-4-like	-2.550086827	0.000163478	-9.083021481	2.13E-07	NA	NA
LOC110377835	uncharacterized protein LOC110377835	-2.4553038	0.000268625	-5.292121722	2.17E-07	NA	NA
LOC110384382	kelch-like protein 10 isoform X4	-1.617320623	0.003606942	-2.960574015	2.36E-07	NA	NA
LOC110384382	kelch-like protein 10 isoform X3	-1.617320623	0.003606942	-2.960574015	2.36E-07	NA	NA
LOC110384382	kelch-like protein 10 isoform X2	-1.617320623	0.003606942	-2.960574015	2.36E-07	NA	NA
LOC110384382	kelch-like protein 10 isoform X1	-1.617320623	0.003606942	-2.960574015	2.36E-07	NA	NA
LOC110377250	endothelin-converting enzyme homolog	-2.531337488	0.000102247	-4.079026616	2.51E-07	NA	NA
LOC110374999	uncharacterized protein LOC110374999	-3.622815258	1.42E-07	-6.825908792	2.51E-07	NA	NA

LOC110378287	uncharacterized protein LOC110378287	-2.201348343	0.001886905	-5.002583362	2.53E-07	NA	NA
LOC110377104	NA	-2.287719084	0.000282603	-6.676199974	2.58E-07	NA	NA
LOC110378143	E3 ubiquitin-protein ligase MARCH3-like isoform X3	-2.506838631	0.000787695	-5.157126551	2.67E-07	NA	NA
LOC110378143	uncharacterized protein LOC110378143 isoform X1	-2.506838631	0.000787695	-5.157126551	2.67E-07	NA	NA
LOC110378143	E3 ubiquitin-protein ligase MARCH3-like isoform X2	-2.506838631	0.000787695	-5.157126551	2.67E-07	NA	NA
LOC110377707	uncharacterized protein LOC110377707 isoform X3	-2.465176967	0.000287052	-4.261269344	2.79E-07	NA	NA
LOC110377707	uncharacterized protein LOC110377707 isoform X2	-2.465176967	0.000287052	-4.261269344	2.79E-07	NA	NA
LOC110377707	uncharacterized protein LOC110377707 isoform X1	-2.465176967	0.000287052	-4.261269344	2.79E-07	NA	NA
LOC110374802	WD repeat-containing protein 75-like	NA	NA	1.644192148	3.05E-07	NA	NA
LOC110371428	uncharacterized protein LOC110371428	-2.293612928	0.007137107	-7.119949016	3.05E-07	NA	NA
LOC110372416	dynein heavy chain 8, axonemal-like	-2.862999466	0.000277542	-4.934523284	3.09E-07	NA	NA
LOC110369731	glutamine synthetase 1, mitochondrial-like	-2.508096509	0.000269014	-4.68151913	3.17E-07	NA	NA

LOC110383188	SPRY domain-containing SOCS box protein 3-like isoform X2	-1.001330842	0.035081055	-2.119116028	3.29E-07	NA	NA
LOC110383188	SPRY domain-containing SOCS box protein 3-like isoform X1	-1.001330842	0.035081055	-2.119116028	3.29E-07	NA	NA
LOC110377335	uncharacterized protein LOC110377335	NA	NA	-1.221511139	3.46E-07	NA	NA
LOC110380818	uncharacterized protein LOC110380818 isoform X3	-2.349293792	0.002254598	-5.162218564	3.46E-07	NA	NA
LOC110380818	uncharacterized protein LOC110380818 isoform X1	-2.349293792	0.002254598	-5.162218564	3.46E-07	NA	NA
LOC110380818	uncharacterized protein LOC110380818 isoform X2	-2.349293792	0.002254598	-5.162218564	3.46E-07	NA	NA
LOC110374280	protein I\\'m not dead yet-like	-2.738875279	0.043731732	-6.49635557	3.46E-07	NA	NA
LOC110381768	serine/arginine-rich splicing factor 4-like isoform X2	-2.705593355	0.000178172	-5.368570022	3.51E-07	NA	NA
LOC110381768	serine/arginine-rich splicing factor 4-like X1	-2.705593355	0.000178172	-5.368570022	3.51E-07	NA	NA
LOC110372476	protein ABHD11-like	-2.842648957	2.10E-06	-6.517034569	3.84E-07	NA	NA
LOC110384406	uncharacterized protein LOC110384406	-3.755537949	7.28E-07	-4.845577683	3.84E-07	NA	NA

LOC110382557	ATP synthase subunit beta, mitochondrial-like, partial	-3.784879114	6.05E-07	-5.378831277	4.03E-07	NA	NA
LOC110380491	titin homolog	-1.631131986	6.57E-05	-2.166275674	4.13E-07	NA	NA
LOC110379481	dynein light chain roadblock-type 1-like	-2.711214839	0.000313661	-5.110023767	4.15E-07	NA	NA
LOC110373581	uncharacterized protein LOC110373581 isoform X1	-2.597591354	0.000684717	-4.921352314	4.16E-07	NA	NA
LOC110373581	uncharacterized protein LOC110373581 isoform X2	-2.597591354	0.000684717	-4.921352314	4.16E-07	NA	NA
LOC110376125	acyl-CoA Delta(11) desaturase-like	NA	NA	1.181032583	4.26E-07	NA	NA
LOC110374758	uncharacterized protein LOC110374758	-2.701760874	1.14E-09	-8.565066285	4.34E-07	NA	NA
LOC110375896	coiled-coil-helix-coiled-coil-helix domain-containing protein 10, mitochondrial-like isoform X1	-2.965333579	4.83E-05	-4.783439507	4.62E-07	NA	NA
LOC110375896	coiled-coil-helix-coiled-coil-helix domain-containing protein 10, mitochondrial-like isoform X2	-2.965333579	4.83E-05	-4.783439507	4.62E-07	NA	NA
LOC110371488	coiled-coil domain-containing protein 39	-1.458921439	0.014588716	-3.085501101	4.70E-07	NA	NA

LOC110374160	adiponectin receptor protein	NA	NA	1.272467292	4.77E-07	NA	NA
LOC110370512	elongation of very long chain fatty acids protein 7-like isoform X2	-2.350298425	0.001949466	-5.057795653	4.96E-07	NA	NA
LOC110370512	elongation of very long chain fatty acids protein 7-like isoform X1	-2.350298425	0.001949466	-5.057795653	4.96E-07	NA	NA
LOC110373081	NA	NA	NA	1.715693465	5.06E-07	NA	NA
LOC110371099	tubulin glycylase 3A-like	-3.079998566	1.34E-05	-6.722735433	5.06E-07	NA	NA
LOC110381433	uncharacterized protein LOC110381433	NA	NA	1.3626307	5.43E-07	NA	NA
LOC110372634	transcriptional regulator ATRX-like	-2.889653623	0.040545955	-6.55958652	5.95E-07	NA	NA
LOC110379314	uncharacterized protein LOC110379314	-2.953602732	4.34E-05	-4.156686838	6.04E-07	NA	NA
LOC110378604	LOW QUALITY PROTEIN: dynein beta chain, ciliary-like	-2.270716161	0.000635786	-3.753843626	6.66E-07	NA	NA
LOC110379985	succinate dehydrogenase cytochrome b560 subunit, mitochondrial-like	-2.808773491	0.000649862	-6.938328808	6.98E-07	NA	NA
LOC110374199	uncharacterized protein LOC110374199	-2.003323464	0.012769146	-5.815366943	8.31E-07	NA	NA
LOC110374100	vegetative cell wall protein gp1-like	-2.679989188	1.14E-05	-6.353843937	8.68E-07	NA	NA

LOC110384177	uncharacterized protein LOC110384177	-2.674161369	0.0001121	-6.5489838	8.79E-07	NA	NA
LOC110375795	malate dehydrogenase, glyoxysomal-like isoform X1	NA	NA	-6.49700771	9.15E-07	NA	NA
LOC110375795	malate dehydrogenase 1, peroxisomal-like isoform X2	NA	NA	-6.49700771	9.15E-07	NA	NA
LOC110382488	uncharacterized protein LOC110382488	-3.312288571	1.96E-05	-4.569003547	9.48E-07	NA	NA
LOC110382734	LOW QUALITY PROTEIN: transient-receptor-potential-like protein	-2.546119918	0.00045211	-5.684086124	9.49E-07	NA	NA
LOC110372115	pyruvate carboxylase, mitochondrial-like	-1.073055058	0.000417089	-1.504871831	9.56E-07	NA	NA
LOC110370751	uncharacterized protein LOC110370751	-2.876657225	0.000157919	-5.290419741	9.90E-07	NA	NA
LOC110377991	uncharacterized protein LOC110377991	NA	NA	1.507005542	1.05E-06	NA	NA
LOC110377750	lipoyltransferase 1, mitochondrial isoform X1	NA	NA	-1.36321409	1.10E-06	NA	NA
LOC110377750	lipoyltransferase 1, mitochondrial isoform X2	NA	NA	-1.36321409	1.10E-06	NA	NA
LOC110377452	chromosome-associated kinesin KIF4 isoform X2	-1.463510461	0.000471204	-2.129834226	1.18E-06	NA	NA
LOC110377452	chromosome-associated kinesin KIF4 isoform X3	-1.463510461	0.000471204	-2.129834226	1.18E-06	NA	NA

LOC110377452	chromosome-associated kinesin KIF4 isoform X1	-1.463510461	0.000471204	-2.129834226	1.18E-06	NA	NA
LOC110384122	uncharacterized protein LOC110384122	-3.064746263	1.12E-05	-6.483980569	1.19E-06	NA	NA
LOC110382733	tektin-1	-2.661009984	8.04E-05	-6.420548797	1.24E-06	NA	NA
LOC110380814	uncharacterized protein LOC110380814 isoform X1	-2.241661159	0.001373902	-8.552126878	1.27E-06	NA	NA
LOC110380814	uncharacterized protein LOC110380814 isoform X2	-2.241661159	0.001373902	-8.552126878	1.27E-06	NA	NA
LOC110375870	uncharacterized protein LOC110375870	-2.246273132	0.000598792	-4.119195791	1.30E-06	NA	NA
LOC110373624	uncharacterized protein LOC110373624	NA	NA	-4.450256332	1.35E-06	NA	NA
LOC110381798	RNA pseudouridylate synthase domain-containing protein 1-like	-1.713601908	3.50E-06	-1.911433143	1.35E-06	NA	NA
LOC110384654	uncharacterized protein LOC110384654	-1.819392785	0.012680203	-5.503612192	1.35E-06	NA	NA
LOC110371962	pyruvate kinase-like	-2.49325386	0.00074166	-5.63219056	1.38E-06	NA	NA
LOC110383025	tubulin beta chain	NA	NA	-6.436327075	1.70E-06	NA	NA
LOC110381028	uncharacterized protein LOC110381028 isoform X1	-2.40512958	0.001252152	-4.787675827	1.70E-06	NA	NA
LOC110381028	uncharacterized protein LOC110381028 isoform X2	-2.40512958	0.001252152	-4.787675827	1.70E-06	NA	NA

LOC110374151	RIB43A-like with coiled-coils protein 2	-2.610126017	0.000843083	-4.926228232	1.70E-06	NA	NA
LOC110376798	NA	-3.123568362	1.16E-05	-8.467068846	1.70E-06	NA	NA
LOC110374258	sodium/potassium/calcium exchanger 4-like	-1.425043747	1.93E-05	-1.683097758	1.72E-06	NA	NA
LOC110379284	serine/arginine repetitive matrix protein 5-like isoform X2	-2.316431054	0.001469788	-4.496800165	1.76E-06	NA	NA
LOC110379284	trichohyalin-like isoform X1	-2.316431054	0.001469788	-4.496800165	1.76E-06	NA	NA
LOC110369704	sodium- and chloride-dependent neutral and basic amino acid transporter B(0+)-like isoform X1	-2.389528225	0.000489871	-4.175660307	1.87E-06	NA	NA
LOC110369704	uncharacterized protein LOC110369704 isoform X2	-2.389528225	0.000489871	-4.175660307	1.87E-06	NA	NA
LOC110370445	tubulin alpha-8 chain-like isoform X1	NA	NA	-6.254233215	1.89E-06	NA	NA
LOC110370445	tubulin alpha-8 chain-like isoform X2	NA	NA	-6.254233215	1.89E-06	NA	NA
LOC110380247	uncharacterized protein LOC110380247	-2.690165082	0.000185135	-5.511891492	1.90E-06	NA	NA
LOC110373446	uncharacterized protein LOC110373446	NA	NA	1.993475874	1.91E-06	NA	NA
LOC110371087	uncharacterized protein LOC110371087	-2.438546435	0.000327654	-6.310336319	1.93E-06	NA	NA

LOC110374403	organic cation/carnitine transporter 7-like	NA	NA	-3.079060789	1.97E-06	NA	NA
LOC110373138	dynein heavy chain 7, axonemal	-1.951233785	0.001804897	-3.265352551	1.99E-06	NA	NA
LOC110374597	uncharacterized protein LOC110374597	NA	NA	1.330522597	2.04E-06	NA	NA
LOC110383952	cytochrome P450 6l1-like	NA	NA	-1.509500424	2.08E-06	NA	NA
LOC110380138	dentin sialophosphoprotein-like	-2.60214244	0.020443999	-4.844852604	2.08E-06	NA	NA
LOC110383727	centrosomal protein of 131 kDa	-2.117604778	0.000491423	-3.181001274	2.10E-06	NA	NA
LOC110375583	serine palmitoyltransferase 1	NA	NA	1.2795141	2.12E-06	NA	NA
LOC110374513	uncharacterized protein LOC110374513	-3.328815952	2.85E-05	-8.528924172	2.22E-06	NA	NA
LOC110378192	uncharacterized protein LOC110378192 isoform X1	-2.301795218	0.000959814	-6.303609363	2.24E-06	NA	NA
LOC110378192	uncharacterized protein LOC110378192 isoform X2	-2.301795218	0.000959814	-6.303609363	2.24E-06	NA	NA
LOC110376022	cilia- and flagella-associated protein 206-like	-2.780692957	4.45E-05	-3.786367755	2.25E-06	NA	NA
LOC110374193	uncharacterized protein LOC110374193	-2.312740419	6.50E-06	-2.662272282	2.39E-06	NA	NA
LOC110370890	uncharacterized protein LOC110370890	-2.549676305	0.00014374	-6.234006754	2.43E-06	NA	NA

LOC110379517	uncharacterized protein LOC110379517 isoform X1	-1.608088406	0.000947079	-2.460965078	2.45E-06	NA	NA
LOC110379517	uncharacterized protein LOC110379517 isoform X2	-1.608088406	0.000947079	-2.460965078	2.45E-06	NA	NA
LOC110379596	LOW QUALITY PROTEIN: uncharacterized protein LOC110379596	NA	NA	2.501564197	2.59E-06	NA	NA
LOC110380870	cyclin-dependent kinase 9 isoform X2	NA	NA	1.849871391	2.62E-06	NA	NA
LOC110380870	cyclin-dependent kinase 9 isoform X1	NA	NA	1.849871391	2.62E-06	NA	NA
LOC110370207	uncharacterized protein LOC110370207	NA	NA	-1.677354948	2.69E-06	NA	NA
LOC110374549	eukaryotic translation initiation factor 4E-1A-like	NA	NA	1.92748525	2.73E-06	NA	NA
LOC110383656	uncharacterized protein LOC110383656, partial	-1.636722733	0.015619785	-6.118145294	2.73E-06	NA	NA
LOC110383267	NA	-2.459661481	0.000798764	-4.646632203	2.73E-06	NA	NA
LOC110373062	leucine zipper putative tumor suppressor 2 homolog isoform X1	NA	NA	1.678222099	2.74E-06	NA	NA
LOC110373062	leucine zipper putative tumor suppressor 2 homolog isoform X2	NA	NA	1.678222099	2.74E-06	NA	NA

LOC110381952	NADP-dependent malic enzyme-like	-2.650403768	2.25E-05	-8.278035859	2.76E-06	NA	NA
LOC110380583	trypsin CFT-1-like	1.591641729	4.34E-05	1.895224339	2.88E-06	NA	NA
LOC110381119	small G protein signaling modulator 3 homolog	NA	NA	-1.319009141	3.07E-06	NA	NA
LOC110374839	ubiquitin carboxyl-terminal hydrolase-like	-2.68873122	0.000190635	-8.281939159	3.11E-06	NA	NA
LOC110381367	uncharacterized protein LOC110381367	-3.267863536	8.57E-05	-8.464721534	3.27E-06	NA	NA
LOC110380076	mitochondrial import receptor subunit TOM40 homolog	-3.452741319	1.98E-05	-8.398145289	3.27E-06	NA	NA
LOC110383992	uncharacterized protein LOC110383992 isoform X3	-2.695030729	0.045486398	-5.745132634	3.34E-06	NA	NA
LOC110383992	uncharacterized protein LOC110383992 isoform X4	-2.695030729	0.045486398	-5.745132634	3.34E-06	NA	NA
LOC110383992	uncharacterized protein LOC110383992 isoform X2	-2.695030729	0.045486398	-5.745132634	3.34E-06	NA	NA
LOC110383992	uncharacterized protein LOC110383992 isoform X1	-2.695030729	0.045486398	-5.745132634	3.34E-06	NA	NA
LOC110369974	ecdysone receptor isoform X4	NA	NA	-1.301302716	3.45E-06	NA	NA
LOC110369974	ecdysone receptor isoform X5	NA	NA	-1.301302716	3.45E-06	NA	NA

LOC110369974	ecdysone receptor isoform X1	NA	NA	-1.301302716	3.45E-06	NA	NA
LOC110369974	ecdysone receptor isoform X2	NA	NA	-1.301302716	3.45E-06	NA	NA
LOC110369974	ecdysone receptor isoform X7	NA	NA	-1.301302716	3.45E-06	NA	NA
LOC110369974	ecdysone receptor isoform X3	NA	NA	-1.301302716	3.45E-06	NA	NA
LOC110369974	ecdysone receptor isoform X6	NA	NA	-1.301302716	3.45E-06	NA	NA
LOC110372835	lysophospholipid acyltransferase 7-like	-2.702748051	0.000205545	-5.383477404	3.51E-06	NA	NA
LOC110377317	NA	-2.257874185	0.005318458	-5.079816729	3.69E-06	NA	NA
LOC110374573	uncharacterized protein LOC110374573	-2.970674196	0.000300389	-5.655567215	3.71E-06	NA	NA
LOC110370490	putative tripartite motif-containing protein 61 isoform X1	-3.053487415	0.000101681	-4.76641648	3.72E-06	NA	NA
LOC110370490	uncharacterized protein LOC110370490 isoform X2	-3.053487415	0.000101681	-4.76641648	3.72E-06	NA	NA
LOC110374858	2-oxoglutarate dehydrogenase-like, mitochondrial, partial	-3.114237583	1.09E-08	-8.049417948	3.74E-06	NA	NA
LOC110372728	cilia- and flagella-associated protein 251-like	-2.413378893	0.006858228	-5.760959875	3.92E-06	NA	NA
LOC110377568	protein KBP homolog	NA	NA	-6.379334436	3.98E-06	NA	NA

LOC110379755	protein lethal(2)essential for life-like	NA	NA	-3.361564584	4.04E-06	NA	NA
LOC110370550	dynein intermediate chain 2, ciliary isoform X2	-2.162450677	0.000371826	-3.139973075	4.12E-06	NA	NA
LOC110370550	dynein intermediate chain 2, ciliary isoform X1	-2.162450677	0.000371826	-3.139973075	4.12E-06	NA	NA
LOC110371826	uncharacterized protein LOC110371826	-2.196347449	0.00165785	-5.248598025	4.12E-06	NA	NA
LOC110382972	uncharacterized protein LOC110382972	-3.258877337	3.99E-05	-8.289667273	4.12E-06	NA	NA
LOC110380421	NA	-2.033208655	0.006646842	-4.322921042	4.22E-06	NA	NA
LOC110373866	uncharacterized protein LOC110373866	-2.582547922	0.000191422	-6.126058268	4.36E-06	NA	NA
LOC110382870	sodium-independent sulfate anion transporter isoform X2	NA	NA	1.250273748	4.37E-06	NA	NA
LOC110382870	sodium-independent sulfate anion transporter isoform X1	NA	NA	1.250273748	4.37E-06	NA	NA
LOC110374626	adenylate kinase 8-like	-2.639600056	0.000287052	-4.058021312	4.51E-06	NA	NA
LOC110376126	uncharacterized protein LOC110376126	-1.652949312	0.002602483	-2.744517376	4.65E-06	NA	NA
LOC110379416	alpha-tocopherol transfer protein	-2.237108414	1.42E-07	-2.121325281	4.65E-06	NA	NA
LOC110384302	uncharacterized protein LOC110384302	-2.473673338	0.001856461	-5.026151467	4.65E-06	NA	NA
LOC110377443	organic cation transporter protein-like	NA	NA	-1.621494811	4.96E-06	NA	NA

LOC110379288	uncharacterized protein LOC110379288	-2.544475673	0.001025125	-4.95051971	5.11E-06	NA	NA
LOC110373001	dynein heavy chain 5, axonemal isoform X1	NA	NA	-3.050767693	5.39E-06	NA	NA
LOC110373001	dynein heavy chain 5, axonemal isoform X2	NA	NA	-3.050767693	5.39E-06	NA	NA
LOC110370510	uncharacterized protein LOC110370510 isoform X3	-2.22705479	0.006739175	-4.127386487	5.47E-06	NA	NA
LOC110370510	uncharacterized protein LOC110370510 isoform X1	-2.22705479	0.006739175	-4.127386487	5.47E-06	NA	NA
LOC110370510	uncharacterized protein LOC110370510 isoform X2	-2.22705479	0.006739175	-4.127386487	5.47E-06	NA	NA
LOC110379371	DNA ligase 1-like	-2.859492255	0.000193203	-5.36910352	5.84E-06	NA	NA
LOC110370477	proton-coupled folate transporter-like	NA	NA	-1.059803923	5.97E-06	NA	NA
LOC110369951	probable chitinase 10 isoform X1	-1.795637747	0.006194911	-3.02702738	6.23E-06	NA	NA
LOC110369951	probable chitinase 10 isoform X2	-1.795637747	0.006194911	-3.02702738	6.23E-06	NA	NA
LOC110379355	GILT-like protein 1	NA	NA	-2.997909595	6.37E-06	NA	NA
LOC110383228	uncharacterized protein LOC110383228	-1.685380504	0.016449545	-3.289323576	6.64E-06	NA	NA
LOC110372877	uncharacterized protein LOC110372877	-3.380787922	0.000168023	-8.331002802	6.64E-06	NA	NA
LOC110378029	uncharacterized protein LOC110378029	-2.173507994	0.012148294	-8.203538008	6.92E-06	NA	NA

LOC110382554	uncharacterized protein LOC110382554	-3.52094898	1.09E-08	-7.932947137	7.17E-06	NA	NA
LOC110380278	guanine nucleotide-binding protein-like 1 isoform X2	NA	NA	1.23080376	7.23E-06	NA	NA
LOC110380278	guanine nucleotide-binding protein-like 1 isoform X1	NA	NA	1.23080376	7.23E-06	NA	NA
LOC110370170	uncharacterized protein LOC110370170 isoform X2	NA	NA	1.294678525	7.25E-06	NA	NA
LOC110370170	uncharacterized protein LOC110370170 isoform X1	NA	NA	1.294678525	7.25E-06	NA	NA
LOC110373882	nuclear pore complex protein Nup153-like isoform X1	NA	NA	-2.649690338	7.32E-06	NA	NA
LOC110373882	nuclear pore complex protein Nup153-like isoform X2	NA	NA	-2.649690338	7.32E-06	NA	NA
LOC110383946	cytochrome b5-related protein-like	NA	NA	-1.563428388	7.46E-06	NA	NA
LOC110377319	uncharacterized protein LOC110377319	-2.71286438	0.000443671	-8.053008931	7.47E-06	NA	NA
LOC110371813	uncharacterized protein LOC110371813	-2.752413528	0.000102247	-6.012716607	7.47E-06	NA	NA
LOC110380256	uncharacterized protein LOC110380256 isoform X1	-1.872700357	0.01015744	-3.26260836	7.58E-06	NA	NA

LOC110380256	enkurin domain-containing protein 1-like isoform X2	-1.872700357	0.01015744	-3.26260836	7.58E-06	NA	NA
LOC110382745	uncharacterized protein LOC110382745	NA	NA	-5.955408489	7.71E-06	NA	NA
LOC110377074	uncharacterized protein LOC110377074	-2.768004911	0.000203325	-6.091312747	7.78E-06	NA	NA
LOC110371298	NA	-1.97285959	0.023782177	-8.123995244	7.96E-06	NA	NA
LOC110383190	dynein intermediate chain 3, ciliary-like isoform X2	-2.195225225	0.006702268	-5.343070686	8.00E-06	NA	NA
LOC110383190	dynein intermediate chain 3, ciliary-like isoform X1	-2.195225225	0.006702268	-5.343070686	8.00E-06	NA	NA
LOC110383190	dynein intermediate chain 3, ciliary-like isoform X3	-2.195225225	0.006702268	-5.343070686	8.00E-06	NA	NA
LOC110379471	galactose-specific cell agglutination protein gsf2-like	-3.583695206	3.39E-06	-6.081698716	8.10E-06	NA	NA
LOC110371744	cytochrome P450 6B5-like	NA	NA	1.165794969	8.66E-06	NA	NA
LOC110374306	serine/threonine-protein kinase pelle-like	NA	NA	1.617468344	9.00E-06	NA	NA
LOC110378663	uncharacterized protein LOC110378663 isoform X1	-2.381572512	0.00063903	-5.932538761	9.08E-06	NA	NA

LOC110378663	uncharacterized protein LOC110378663 isoform X2	-2.381572512	0.00063903	-5.932538761	9.08E-06	NA	NA
LOC110372477	protein ABHD11-like	-2.631250566	0.001070611	-8.03731474	9.18E-06	NA	NA
LOC110376853	spidroin-2-like	-2.820975574	0.000414305	-4.285767952	9.46E-06	NA	NA
LOC110379411	uncharacterized protein LOC110379411	-2.327903812	0.005151753	-5.374198539	9.72E-06	NA	NA
LOC110375020	protein FAM13A isoform X1	-1.686370696	0.011825137	-3.079481825	1.00E-05	NA	NA
LOC110375020	protein FAM13A isoform X2	-1.686370696	0.011825137	-3.079481825	1.00E-05	NA	NA
LOC110375020	protein FAM13A isoform X3	-1.686370696	0.011825137	-3.079481825	1.00E-05	NA	NA
LOC110371672	zonadhesin	NA	NA	2.459142583	1.03E-05	NA	NA
LOC110380915	cilia- and flagella-associated protein 58-like	-3.029388014	0.000343764	-8.062602989	1.03E-05	NA	NA
LOC110370293	uncharacterized protein LOC110370293	-2.622533571	0.004207091	-5.602865691	1.09E-05	NA	NA
LOC110376967	nucleolar complex protein 2 homolog	NA	NA	1.233150244	1.10E-05	NA	NA
LOC110382223	uncharacterized protein LOC110382223 isoform X1	-2.735570459	0.000168565	-5.946789534	1.11E-05	NA	NA
LOC110382223	uncharacterized protein LOC110382223 isoform X2	-2.735570459	0.000168565	-5.946789534	1.11E-05	NA	NA
LOC110379482	uncharacterized protein LOC110379482	-2.794488555	0.004020078	-8.266718903	1.11E-05	NA	NA

LOC110374988	aminoacylase-1-like	NA	NA	-1.039113551	1.17E-05	NA	NA
LOC110379225	sodium/potassium-transporting ATPase subunit alpha-like	-2.145750555	0.017594595	-8.057542555	1.17E-05	NA	NA
LOC110378001	uncharacterized protein C1orf194	NA	NA	-5.276176302	1.20E-05	NA	NA
LOC110380384	lysoplasmalogenase-like protein TMEM86A	NA	NA	-1.389977636	1.31E-05	NA	NA
LOC110376833	riboflavin kinase	NA	NA	-1.135193066	1.31E-05	NA	NA
LOC110379532	zinc finger C2HC domain-containing protein 1C isoform X2	-1.74396231	7.82E-09	-1.449776239	1.37E-05	NA	NA
LOC110379532	zinc finger C2HC domain-containing protein 1C isoform X3	-1.74396231	7.82E-09	-1.449776239	1.37E-05	NA	NA
LOC110379532	zinc finger C2HC domain-containing protein 1C isoform X1	-1.74396231	7.82E-09	-1.449776239	1.37E-05	NA	NA
LOC110370026	NA	-2.733528387	0.001501219	-5.402607892	1.46E-05	NA	NA
LOC110375539	beta-1,3-glucan-binding protein-like	-2.78091226	2.98E-11	-2.009141478	1.48E-05	NA	NA
LOC110370600	uncharacterized protein LOC110370600	NA	NA	-6.276883791	1.56E-05	NA	NA
LOC110372286	uncharacterized protein LOC110372286 isoform X2	-2.360650551	0.001181531	-3.66266201	1.57E-05	NA	NA

LOC110372286	uncharacterized protein LOC110372286 isoform X1	-2.360650551	0.001181531	-3.66266201	1.57E-05	NA	NA
LOC110370841	uncharacterized protein LOC110370841	NA	NA	1.205801789	1.62E-05	NA	NA
LOC110376797	NA	-3.0009298	0.000126031	-4.676094895	1.63E-05	NA	NA
LOC110380161	monoacylglycerol lipase ABHD12-like	-2.581271057	0.000654017	-5.904173003	1.64E-05	NA	NA
LOC110378417	uncharacterized protein LOC110378417 isoform X2	-2.385278762	0.00227124	-5.946570886	1.65E-05	NA	NA
LOC110378417	uncharacterized protein LOC110378417 isoform X3	-2.385278762	0.00227124	-5.946570886	1.65E-05	NA	NA
LOC110378417	uncharacterized protein LOC110378417 isoform X1	-2.385278762	0.00227124	-5.946570886	1.65E-05	NA	NA
LOC110378417	uncharacterized protein LOC110378417 isoform X4	-2.385278762	0.00227124	-5.946570886	1.65E-05	NA	NA
LOC110384190	adenylate kinase 7-like	-3.051566431	4.02E-06	-7.734830028	1.65E-05	NA	NA
LOC110373751	uncharacterized protein LOC110373751	-2.376707867	0.008798319	-7.963706746	1.66E-05	NA	NA
LOC110371978	U3 small nucleolar RNA-associated protein 6 homolog	NA	NA	1.381206999	1.68E-05	NA	NA
LOC110374139	uncharacterized protein K02A2.6-like	NA	NA	2.288534939	1.68E-05	NA	NA

LOC110370156	uncharacterized protein LOC110370156	-3.241372076	9.38E-05	-5.244454751	1.69E-05	NA	NA
LOC110378364	fas-binding factor 1 homolog	-2.399469877	0.000914499	-3.58042812	1.70E-05	NA	NA
LOC110380975	uncharacterized protein LOC110380975	-2.76664869	0.004926916	-8.117508825	1.70E-05	NA	NA
LOC110372983	ATPase inhibitor A, mitochondrial-like	-2.818534313	0.000929201	-5.330210653	1.70E-05	NA	NA
LOC110381838	uncharacterized protein LOC110381838	-1.884826713	0.014564019	-3.414581687	1.75E-05	NA	NA
LOC110377836	sorting and assembly machinery component 50 homolog A-like	-2.20873971	0.010013903	-4.534674293	1.93E-05	NA	NA
LOC110376104	long-chain-fatty-acid--CoA ligase 5 isoform X2	NA	NA	-1.787691042	1.96E-05	NA	NA
LOC110376104	long-chain-fatty-acid--CoA ligase 5 isoform X1	NA	NA	-1.787691042	1.96E-05	NA	NA
LOC110376104	long-chain-fatty-acid--CoA ligase 5 isoform X3	NA	NA	-1.787691042	1.96E-05	NA	NA
LOC110376879	WD repeat-containing protein on Y chromosome-like	-2.679954173	0.002468978	-6.143461881	1.98E-05	NA	NA
LOC110370786	uncharacterized protein LOC110370786	-3.131923884	0.000124898	-4.723259922	2.02E-05	NA	NA
LOC110382752	UDP-glucuronosyltransferase 2B17-like	NA	NA	-2.561053677	2.04E-05	NA	NA
LOC110381066	NA	NA	NA	-1.451071509	2.04E-05	NA	NA

LOC110372326	uncharacterized protein LOC110372326	-2.799590381	0.000331245	-5.883698633	2.09E-05	NA	NA
LOC110378476	cytochrome P450 9e2-like	NA	NA	1.494466146	2.14E-05	NA	NA
LOC110375903	uncharacterized protein LOC110375903	-1.951139417	1.36E-05	-2.049111707	2.14E-05	NA	NA
LOC110381640	uncharacterized protein LOC110381640 isoform X1	-2.035633017	1.31E-05	-2.129625676	2.14E-05	NA	NA
LOC110381640	uncharacterized protein LOC110381640 isoform X2	-2.035633017	1.31E-05	-2.129625676	2.14E-05	NA	NA
LOC110381640	uncharacterized protein LOC110381640 isoform X3	-2.035633017	1.31E-05	-2.129625676	2.14E-05	NA	NA
LOC110380917	cilia- and flagella-associated protein 58-like	-2.690350314	0.001041793	-5.960633522	2.23E-05	NA	NA
LOC110377303	uncharacterized protein LOC110377303	-2.16061871	0.005098815	-5.797882772	2.31E-05	NA	NA
LOC110374991	uncharacterized protein LOC110374991	-3.113809151	0.000466794	-4.951296469	2.31E-05	NA	NA
LOC110374033	NA	-2.724369493	0.000541794	-5.866310505	2.34E-05	NA	NA
LOC110383052	uncharacterized protein LOC110383052	-2.081839644	0.008170249	-4.087365431	2.37E-05	NA	NA
LOC110377355	uncharacterized protein LOC110377355 isoform X2	-1.110205969	0.019065102	-1.893899685	2.38E-05	NA	NA

LOC110377355	uncharacterized protein LOC110377355 isoform X1	-1.110205969	0.019065102	-1.893899685	2.38E-05	NA	NA
LOC110380303	putative WEB family protein At1g65010, chloroplastic	-2.23086994	0.001147614	-7.616424667	2.38E-05	NA	NA
LOC110373692	probable E3 ubiquitin-protein ligase sinah isoform X2	-2.656499242	5.81E-14	-1.672140198	2.38E-05	NA	NA
LOC110373692	probable E3 ubiquitin-protein ligase sinah isoform X1	-2.656499242	5.81E-14	-1.672140198	2.38E-05	NA	NA
LOC110383216	trichohyalin-like isoform X1	NA	NA	-6.021108456	2.41E-05	NA	NA
LOC110383216	trichohyalin-like isoform X2	NA	NA	-6.021108456	2.41E-05	NA	NA
LOC110383216	golgin subfamily B member 1-like isoform X3	NA	NA	-6.021108456	2.41E-05	NA	NA
LOC110371309	uncharacterized protein LOC110371309	-2.438606754	0.007007226	-5.31886597	2.42E-05	NA	NA
LOC110377245	phosphate carrier protein, mitochondrial-like	-2.400402559	0.002219224	-5.823541297	2.53E-05	NA	NA
LOC110374025	chymotrypsin-like protease CTRL-1	-2.556888349	0.001052649	-5.82567314	2.53E-05	NA	NA
LOC110380720	dynein assembly factor with WDR repeat domains 1	-3.456931384	0.000237724	-5.473766678	2.56E-05	NA	NA

LOC110383969	uncharacterized protein LOC110383969	NA	NA	-5.79958807	2.58E-05	NA	NA
LOC110382239	uncharacterized protein LOC110382239	-2.157085295	0.008391296	-4.0375552	2.58E-05	NA	NA
LOC110378465	sporozoite surface protein 2-like	-2.053845273	0.001043696	-7.492260032	2.61E-05	NA	NA
LOC110382990	uncharacterized protein LOC110382990	-2.638071458	0.000119921	-3.239596695	2.61E-05	NA	NA
LOC110373566	uncharacterized protein LOC110373566	NA	NA	1.420056072	2.62E-05	NA	NA
LOC110370304	protein canopy homolog 3	NA	NA	1.236206035	2.64E-05	NA	NA
LOC110383962	uncharacterized protein LOC110383962	-2.768097182	2.45E-07	-2.458222767	2.66E-05	NA	NA
LOC110379431	protein lethal(2)essential for life-like	-2.624045944	0.004851134	-7.822796451	2.67E-05	NA	NA
LOC110382152	radial spoke head protein 6 homolog A	-2.782762534	0.0006597	-5.097710468	2.67E-05	NA	NA
LOC110382754	UDP-glucuronosyltransferase 2B17-like isoform X1	NA	NA	-2.936950454	2.70E-05	NA	NA
LOC110382754	UDP-glucuronosyltransferase 2B17-like isoform X2	NA	NA	-2.936950454	2.70E-05	NA	NA
LOC110382754	UDP-glucuronosyltransferase 2B19-like isoform X3	NA	NA	-2.936950454	2.70E-05	NA	NA
LOC110375492	uncharacterized protein LOC110375492	1.157974457	0.011730546	1.813648226	2.70E-05	NA	NA

LOC110371510	collagen alpha-1(III) chain-like isoform X1	-1.574582347	0.000124266	-1.818178	2.77E-05	NA	NA
LOC110371510	collagen alpha-1(III) chain-like isoform X3	-1.574582347	0.000124266	-1.818178	2.77E-05	NA	NA
LOC110371510	collagen alpha-1(III) chain-like isoform X2	-1.574582347	0.000124266	-1.818178	2.77E-05	NA	NA
LOC110379945	uncharacterized protein LOC110379945	-2.226908007	0.007074947	-4.362932706	2.84E-05	NA	NA
LOC110380714	uncharacterized protein LOC110380714	-2.608858319	0.001635029	-4.428761658	2.84E-05	NA	NA
LOC110378771	uncharacterized protein LOC110378771	NA	NA	1.273526507	2.85E-05	NA	NA
LOC110383242	uncharacterized protein LOC110383242	-1.666694037	0.030488483	-3.629936775	2.85E-05	NA	NA
LOC110380922	NADH dehydrogenase	-1.869164024	0.027808974	-4.590800799	2.85E-05	NA	NA
LOC110374519	V-type proton ATPase 116 kDa subunit a-like	-3.506535455	3.38E-05	-4.388560819	2.97E-05	NA	NA
LOC110377280	uncharacterized protein LOC110377280	-2.801588286	0.003939074	-7.846510405	3.03E-05	NA	NA
LOC110383961	cytochrome P450 6l1-like	NA	NA	-1.581858608	3.06E-05	NA	NA
LOC110376741	paramyosin-like	-2.047211398	1.38E-05	-2.121566928	3.09E-05	NA	NA
LOC110374363	uncharacterized protein LOC110374363	-3.155716953	6.11E-05	-5.746498393	3.09E-05	NA	NA
LOC110380880	uncharacterized protein LOC110380880 isoform X3	-2.012090795	0.002142965	-2.97563632	3.10E-05	NA	NA

LOC110380880	uncharacterized protein LOC110380880 isoform X1	-2.012090795	0.002142965	-2.97563632	3.10E-05	NA	NA
LOC110380880	uncharacterized protein LOC110380880 isoform X2	-2.012090795	0.002142965	-2.97563632	3.10E-05	NA	NA
LOC110380446	uncharacterized protein LOC110380446	-3.212459017	3.74E-06	-7.534380694	3.10E-05	NA	NA
LOC110376517	cytochrome P450 9e2-like	-7.494108233	3.67E-09	-5.56314076	3.35E-05	NA	NA
LOC110378654	uncharacterized protein LOC110378654	NA	NA	-3.993863353	3.48E-05	NA	NA
LOC110373476	uncharacterized protein LOC110373476	-2.68541831	0.000366487	-3.721341204	3.48E-05	NA	NA
LOC110376428	uncharacterized protein LOC110376428	NA	NA	1.254667231	3.52E-05	NA	NA
LOC110372063	DNA-directed RNA polymerase III subunit RPC5	NA	NA	1.37609276	3.52E-05	NA	NA
LOC110379984	succinate dehydrogenase	NA	NA	-6.682392493	3.60E-05	NA	NA
LOC110378600	alcohol dehydrogenase	NA	NA	-1.543964288	3.61E-05	NA	NA
LOC110378600	uncharacterized protein LOC110378600 isoform X1	NA	NA	-1.543964288	3.61E-05	NA	NA
LOC110382644	ribonuclease UK114-like	-2.412614961	0.004731078	-5.075306633	3.68E-05	NA	NA
LOC110371723	uncharacterized protein LOC110371723	-2.629451048	0.00191147	-5.850447016	3.70E-05	NA	NA

LOC110378186	proton-coupled amino acid transporter-like protein CG1139	-1.968445414	0.031382504	-4.130026193	3.71E-05	NA	NA
LOC110379983	glutamine synthetase 2 cytoplasmic-like	NA	NA	-1.248698926	3.74E-05	NA	NA
LOC110381627	leucine-rich repeat-containing protein 71-like	-2.645156625	0.001253884	-3.88340573	3.76E-05	NA	NA
LOC110369602	uncharacterized protein LOC110369602	-3.212385047	6.88E-05	-5.716894651	3.86E-05	NA	NA
LOC110377222	uncharacterized protein LOC110377222	NA	NA	-5.75749991	3.88E-05	NA	NA
LOC110380982	uncharacterized protein LOC110380982	-3.690671871	2.72E-06	-7.59370123	3.88E-05	NA	NA
LOC110379375	OCIA domain-containing protein 1-like	-2.539872901	0.001959003	-4.991047096	3.93E-05	NA	NA
LOC110381057	UPF0605 protein CG18335	-2.098444306	0.013611066	-4.566765903	4.08E-05	NA	NA
LOC110378191	uncharacterized protein LOC110378191	-2.57411917	0.000168023	-7.401961158	4.28E-05	NA	NA
LOC110369711	peroxisomal acyl-coenzyme A oxidase 3	NA	NA	1.76595419	4.35E-05	NA	NA
LOC110377935	elongation factor 1-alpha 2	-1.701561775	0.002230591	-2.306177669	4.42E-05	NA	NA
LOC110382979	alanine aminotransferase 2-like	NA	NA	-6.266808636	4.49E-05	NA	NA
LOC110372857	uncharacterized protein LOC110372857	-1.978578211	0.010531245	-5.571760712	4.49E-05	NA	NA

LOC110375143	nucleoside diphosphate kinase 7-like	-3.094641214	3.80E-06	-7.352367213	4.50E-05	NA	NA
LOC110378117	carboxypeptidase B-like isoform X1	-2.822685112	0.005800828	-7.764496191	4.51E-05	NA	NA
LOC110378117	carboxypeptidase B-like isoform X2	-2.822685112	0.005800828	-7.764496191	4.51E-05	NA	NA
LOC110381123	pseudouridine-5\\'-phosphatase-like	-2.952984275	0.000292355	-5.70632342	4.52E-05	NA	NA
LOC110373949	LOW QUALITY PROTEIN: uncharacterized protein LOC110373949	-2.52528563	0.01343809	-7.710566696	4.80E-05	NA	NA
LOC110379285	uncharacterized protein LOC110379285	-2.890661014	0.000899266	-5.068592179	4.92E-05	NA	NA
LOC110382064	NADH dehydrogenase	-3.33061522	0.001189487	-7.733608188	4.92E-05	NA	NA
LOC110376469	uncharacterized protein LOC110376469	-2.668971341	0.001219348	-5.695662748	5.18E-05	NA	NA
LOC110370909	farnesol dehydrogenase-like	-2.727429452	0.000787695	-3.662855208	5.26E-05	NA	NA
LOC110373865	uncharacterized protein LOC110373865	-1.858793886	0.039360449	-4.94552457	5.31E-05	NA	NA
LOC110376593	exonuclease 3\\'-5\\' domain-containing protein 2	NA	NA	1.345402798	5.34E-05	NA	NA
LOC110381133	outer dense fiber protein 3-like protein 2	-2.198169053	0.048099552	-7.733495821	5.34E-05	NA	NA
LOC110376232	phospholipid phosphatase 2-like isoform X1	NA	NA	1.50689203	5.45E-05	NA	NA

LOC110376232	phospholipid phosphatase 2-like isoform X2	NA	NA	1.50689203	5.45E-05	NA	NA
LOC110375980	cytochrome c oxidase subunit 6B1-like	-1.942323247	0.007740148	-3.21015644	5.59E-05	NA	NA
LOC110377969	kelch-like protein 10	-2.790969879	0.040952849	-8.228370806	5.69E-05	NA	NA
LOC110370036	cytochrome P450 6a2-like	NA	NA	1.175319186	5.71E-05	NA	NA
LOC110377188	uncharacterized protein LOC110377188 isoform X1	-2.617507889	0.002806579	-5.009638894	6.14E-05	NA	NA
LOC110377188	uncharacterized protein LOC110377188 isoform X2	-2.617507889	0.002806579	-5.009638894	6.14E-05	NA	NA
LOC110369690	glutamine synthetase 1, mitochondrial-like	-2.799081179	0.000899266	-4.114753697	6.16E-05	NA	NA
LOC110376364	uncharacterized protein LOC110376364 isoform X3	-3.122461536	0.000297512	-5.716694091	6.18E-05	NA	NA
LOC110376364	uncharacterized protein LOC110376364 isoform X2	-3.122461536	0.000297512	-5.716694091	6.18E-05	NA	NA
LOC110376364	uncharacterized protein LOC110376364 isoform X1	-3.122461536	0.000297512	-5.716694091	6.18E-05	NA	NA
LOC110380657	ATP synthase subunit b, mitochondrial-like	-3.611756194	0.000886549	-7.721964637	6.19E-05	NA	NA
LOC110379589	membrane-bound alkaline phosphatase-like	NA	NA	1.463630237	6.26E-05	NA	NA

LOC110369973	uncharacterized protein LOC110369973	-3.453520793	0.000117699	-5.762605262	6.41E-05	NA	NA
LOC110384543	beta-hexosaminidase subunit beta-like isoform X1	NA	NA	-1.554254425	6.44E-05	NA	NA
LOC110384543	beta-hexosaminidase subunit beta-like isoform X2	NA	NA	-1.554254425	6.44E-05	NA	NA
LOC110376836	uncharacterized protein LOC110376836	-2.603441558	0.001514806	-4.851374482	6.47E-05	NA	NA
LOC110372172	LOW QUALITY PROTEIN: uncharacterized protein LOC110372172	NA	NA	-4.364683925	6.56E-05	NA	NA
LOC110379942	uncharacterized protein LOC110379942	-2.583808097	0.00253604	-5.677378979	6.61E-05	NA	NA
LOC110371551	caspase-1-like	NA	NA	2.005908602	6.70E-05	NA	NA
LOC110372919	17-beta-hydroxysteroid dehydrogenase 13-like	-2.785420099	0.003323736	-4.796266756	6.94E-05	NA	NA
LOC110381020	uncharacterized protein LOC110381020	-3.301004092	0.001433076	-7.567104634	7.26E-05	NA	NA
LOC110378413	delta-1-pyrroline-5-carboxylate synthase	NA	NA	-1.476497664	7.29E-05	NA	NA
LOC110378782	cilia- and flagella-associated protein 52	-1.533136361	0.014521017	-2.475719146	7.29E-05	NA	NA
LOC110369737	WD repeat-containing protein 78-like	-3.00524266	0.000194997	-3.640402642	7.29E-05	NA	NA
LOC110373031	uncharacterized protein LOC110373031	-2.413613907	0.003958666	-4.184756322	7.33E-05	NA	NA

LOC110374592	cilia- and flagella-associated protein 157-like	-3.207338384	0.000508139	-5.804926729	7.37E-05	NA	NA
LOC110376557	mitochondrial thiamine pyrophosphate carrier-like	-2.549066618	0.001043696	-3.640328583	7.43E-05	NA	NA
LOC110374118	proclotting enzyme-like	-1.807649986	0.010844458	-3.142510322	7.44E-05	NA	NA
LOC110378902	uncharacterized protein LOC110378902	-1.389554748	0.00854403	-2.119561392	7.53E-05	NA	NA
LOC110382802	cyclin-dependent kinase-like 1, partial	NA	NA	-4.895856981	7.75E-05	NA	NA
LOC110372467	NA	-3.294681106	0.001755093	-7.560732422	7.93E-05	NA	NA
LOC110374113	uncharacterized protein LOC110374113	NA	NA	-5.908112715	8.11E-05	NA	NA
LOC110384564	uncharacterized protein LOC110384564	-2.710536835	0.0010822	-4.803114975	8.11E-05	NA	NA
LOC110371976	LOW QUALITY PROTEIN: protein N-terminal asparagine amidohydrolase	NA	NA	1.007817727	8.16E-05	NA	NA
LOC110374101	small proline-rich protein 2H-like	-2.223600889	0.04823475	-7.575996094	8.16E-05	NA	NA
LOC110369724	leucine-rich repeat-containing protein 74B-like	-3.246196361	0.000572087	-5.816965692	8.16E-05	NA	NA
LOC110373730	NA	-3.168469166	3.42E-05	-7.269978863	8.21E-05	NA	NA
LOC110378391	uncharacterized protein LOC110378391 isoform X1	-3.315640294	0.002123047	-7.595529676	8.22E-05	NA	NA

LOC110378391	uncharacterized protein LOC110378391 isoform X2	-3.315640294	0.002123047	-7.595529676	8.22E-05	NA	NA
LOC110374740	coiled-coil domain-containing protein 65-like	-2.612755253	0.003862643	-5.699555812	8.27E-05	NA	NA
LOC110371271	probable beta-hexosaminidase fdl	-2.545831779	0.004383398	-4.320748778	8.45E-05	NA	NA
LOC110383067	uncharacterized protein LOC110383067	-2.964051343	0.00608641	-6.089030366	9.00E-05	NA	NA
LOC110374835	erythrocyte band 7 integral membrane protein-like isoform X2	-3.178639822	0.000959814	-5.838032392	9.00E-05	NA	NA
LOC110374835	band 7 protein AGAP004871-like isoform X1	-3.178639822	0.000959814	-5.838032392	9.00E-05	NA	NA
LOC110374835	mechanosensory protein 2-like isoform X3	-3.178639822	0.000959814	-5.838032392	9.00E-05	NA	NA
LOC110371125	uncharacterized protein LOC110371125 isoform X1	-2.587864667	0.00228803	-4.425044192	9.04E-05	NA	NA
LOC110371125	uncharacterized protein LOC110371125 isoform X2	-2.587864667	0.00228803	-4.425044192	9.04E-05	NA	NA
LOC110370799	myb-binding protein 1A-like protein	NA	NA	1.271158522	9.10E-05	NA	NA
LOC110376964	alpha-(1,3)-fucosyltransferase C-like	-2.897297643	0.007801674	-7.55100252	9.44E-05	NA	NA
LOC110381481	uncharacterized protein LOC110381481	-3.252192335	0.000945101	-5.185798205	9.52E-05	NA	NA

LOC110382328	alpha-1,6-mannosyl-glycoprotein 2-beta-N-acetylglucosaminyltransferase-like	-2.97051134	0.000336677	-5.478380115	9.64E-05	NA	NA
LOC110376018	lebercilin-like protein	-2.832918948	8.72E-06	-2.703569878	9.90E-05	NA	NA
LOC110376947	NADH-ubiquinone oxidoreductase 49 kDa subunit-like	-1.767574324	0.007580918	-2.755090022	0.00010079	NA	NA
LOC110377712	beta-1,4-galactosyltransferase 1-like	-1.496360563	0.001523922	-1.91317158	0.000101233	NA	NA
LOC110377173	NA	-1.455176434	0.001726834	-1.876855647	0.00010143	NA	NA
LOC110372001	probable calcium-binding protein CML14	-2.361922413	0.000712158	-7.076821452	0.000102629	NA	NA
LOC110372876	uncharacterized protein LOC110372876	-2.465475324	0.000572087	-7.104838257	0.000102629	NA	NA
LOC110376716	uncharacterized protein LOC110376716	-2.354244401	0.034827217	-7.480966289	0.000103339	NA	NA
LOC110376425	protein distal antenna-like	NA	NA	-1.39837819	0.000107588	NA	NA
LOC110379170	uncharacterized protein LOC110379170 isoform X3	-1.275311693	0.002655442	-1.670231345	0.000109629	NA	NA
LOC110379170	uncharacterized protein LOC110379170 isoform X5	-1.275311693	0.002655442	-1.670231345	0.000109629	NA	NA
LOC110379170	uncharacterized protein LOC110379170 isoform X1	-1.275311693	0.002655442	-1.670231345	0.000109629	NA	NA

LOC110379170	uncharacterized protein LOC110379170 isoform X2	-1.275311693	0.002655442	-1.670231345	0.000109629	NA	NA
LOC110379170	uncharacterized protein LOC110379170 isoform X6	-1.275311693	0.002655442	-1.670231345	0.000109629	NA	NA
LOC110379170	uncharacterized protein LOC110379170 isoform X4	-1.275311693	0.002655442	-1.670231345	0.000109629	NA	NA
LOC110379360	uncharacterized protein LOC110379360	-2.838226295	0.002027943	-5.660102139	0.000110052	NA	NA
LOC110369803	uncharacterized protein LOC110369803	-2.591913951	0.003286446	-3.684022861	0.000110258	NA	NA
LOC110376754	uncharacterized protein LOC110376754 isoform X1	-2.934639381	0.000698165	-5.523559025	0.000110258	NA	NA
LOC110376754	uncharacterized protein LOC110376754 isoform X2	-2.934639381	0.000698165	-5.523559025	0.000110258	NA	NA
LOC110374998	uncharacterized protein LOC110374998	-3.582272273	6.86E-06	-7.178751973	0.000110258	NA	NA
LOC110381494	uncharacterized protein LOC110381494 isoform X1	NA	NA	1.466838963	0.000111796	NA	NA
LOC110381494	uncharacterized protein LOC110381494 isoform X2	NA	NA	1.466838963	0.000111796	NA	NA
LOC110381554	uncharacterized protein LOC110381554	NA	NA	2.000626508	0.000111796	NA	NA

LOC110369705	armadillo repeat-containing protein gudu	-2.11237368	0.001373902	-2.792990771	0.000111998	NA	NA
LOC110371476	serologically defined colon cancer antigen 8 homolog	-1.214980431	0.025120243	-2.055902285	0.000112114	NA	NA
LOC110380287	uncharacterized protein LOC110380287 isoform X1	-2.092904809	0.022522383	-4.827133688	0.000113209	NA	NA
LOC110380287	uncharacterized protein LOC110380287 isoform X2	-2.092904809	0.022522383	-4.827133688	0.000113209	NA	NA
LOC110369618	aldo-keto reductase AKR2E4-like isoform X1	NA	NA	1.014310458	0.000113448	NA	NA
LOC110369618	aldo-keto reductase AKR2E4-like isoform X5	NA	NA	1.014310458	0.000113448	NA	NA
LOC110369618	aldo-keto reductase AKR2E4-like isoform X4	NA	NA	1.014310458	0.000113448	NA	NA
LOC110369618	aldo-keto reductase AKR2E4-like isoform X3	NA	NA	1.014310458	0.000113448	NA	NA
LOC110369618	aldo-keto reductase AKR2E4-like isoform X2	NA	NA	1.014310458	0.000113448	NA	NA
LOC110376850	UDP-glucuronosyltransferase 2B13-like	NA	NA	-1.956280577	0.000117017	NA	NA
LOC110371394	facilitated trehalose transporter Tret1-like isoform X2	NA	NA	-1.799303907	0.000117238	NA	NA

LOC110371394	facilitated trehalose transporter Tret1-like isoform X1	NA	NA	-1.799303907	0.000117238	NA	NA
LOC110370984	uncharacterized protein LOC110370984	-2.122305	0.000102587	-2.241574307	0.000118004	NA	NA
LOC110382776	putative fatty acyl-CoA reductase CG5065	NA	NA	1.753592771	0.000118665	NA	NA
LOC110379002	uncharacterized protein LOC110379002	-2.800826588	0.001458619	-4.816252239	0.000118833	NA	NA
LOC110384514	brachyurin-like	NA	NA	-1.432923933	0.000119863	NA	NA
LOC110379267	tubulin alpha-1 chain-like	NA	NA	-6.265361891	0.000120847	NA	NA
LOC110376366	uncharacterized protein LOC110376366	-3.423176676	2.47E-05	-7.17071579	0.000121396	NA	NA
LOC110382173	IQ domain-containing protein G-like isoform X2	-2.747771242	0.001875419	-3.831066623	0.000122693	NA	NA
LOC110382173	IQ domain-containing protein G-like isoform X1	-2.747771242	0.001875419	-3.831066623	0.000122693	NA	NA
LOC110378758	protein-glucosylgalactosylhydroxylysine glucosidase isoform X2	-1.756563674	0.012987801	-2.856150922	0.000123476	NA	NA
LOC110378758	protein-glucosylgalactosylhydroxylysine glucosidase isoform X1	-1.756563674	0.012987801	-2.856150922	0.000123476	NA	NA
LOC110378553	trypsin, alkaline B-like	NA	NA	-1.591271968	0.000127458	NA	NA
LOC110381716	leucine-rich repeat-containing G-protein coupled receptor 5	NA	NA	1.285533338	0.000127917	NA	NA

LOC110373673	NA	-2.800984867	0.001989777	-7.306592632	0.000128811	NA	NA
LOC110371086	uricase	3.012037731	3.24E-07	2.484734829	0.000129796	NA	NA
LOC110380974	uncharacterized protein LOC110380974 isoform X1	-2.819013766	0.000102587	-7.017534117	0.000129968	NA	NA
LOC110380974	uncharacterized protein LOC110380974 isoform X2	-2.819013766	0.000102587	-7.017534117	0.000129968	NA	NA
LOC110380077	TBC1 domain family member 31	-1.784652448	6.38E-05	-1.84471609	0.000131833	NA	NA
LOC110379964	pregnancy zone protein-like	NA	NA	-2.648789295	0.000133252	NA	NA
LOC110373974	piggyBac transposable element-derived protein 3-like	NA	NA	-7.628978817	0.000133712	NA	NA
LOC110369829	uncharacterized protein LOC110369829	NA	NA	-7.268635997	0.000150157	NA	NA
LOC110371615	uncharacterized protein LOC110371615 isoform X4	-1.07960473	0.000882746	-1.277913542	0.000152386	NA	NA
LOC110371615	uncharacterized protein LOC110371615 isoform X1	-1.07960473	0.000882746	-1.277913542	0.000152386	NA	NA
LOC110371615	uncharacterized protein LOC110371615 isoform X3	-1.07960473	0.000882746	-1.277913542	0.000152386	NA	NA
LOC110371615	uncharacterized protein LOC110371615 isoform X2	-1.07960473	0.000882746	-1.277913542	0.000152386	NA	NA

LOC110376952	zinc transporter ZIP10	NA	NA	-1.30351407	0.000152774	NA	NA
LOC110384652	coiled-coil domain-containing protein 13	-2.876492327	0.001991159	-5.564024555	0.000152923	NA	NA
LOC110376114	uncharacterized protein LOC110376114	-2.719691869	0.00026001	-6.977341536	0.000155196	NA	NA
LOC110370515	uncharacterized protein LOC110370515	-2.712015417	0.001383492	-7.132736647	0.000156533	NA	NA
LOC110382324	proline-rich protein 12-like isoform X3	NA	NA	-5.65950361	0.000156553	NA	NA
LOC110382324	proline-rich protein 12-like isoform X2	NA	NA	-5.65950361	0.000156553	NA	NA
LOC110382324	proline-rich protein 12-like isoform X1	NA	NA	-5.65950361	0.000156553	NA	NA
LOC110374420	uncharacterized protein LOC110374420	-3.243762076	0.000886549	-4.981274644	0.000156553	NA	NA
LOC110373872	uncharacterized protein LOC110373872	-3.087397555	0.000114057	-7.054617145	0.000156724	NA	NA
LOC110372669	axoneme-associated protein mst101(2)-like	NA	NA	-5.877721902	0.000162953	NA	NA
LOC110381365	E3 ubiquitin-protein ligase CBL-B-B isoform X1	NA	NA	1.718944031	0.000163693	NA	NA
LOC110381365	E3 ubiquitin-protein ligase CBL-B-B isoform X2	NA	NA	1.718944031	0.000163693	NA	NA
LOC110382492	secretory phospholipase A2 receptor-like	NA	NA	-2.380700724	0.000163722	NA	NA

LOC110380790	uncharacterized protein LOC110380790	NA	NA	1.460538023	0.000164618	NA	NA
LOC110383057	sideroflexin-3-like	-2.641931094	0.011697484	-5.096355741	0.000168373	NA	NA
LOC110374045	uncharacterized protein LOC110374045 isoform X1	-2.180726472	0.031563067	-4.549831861	0.000170339	NA	NA
LOC110374045	uncharacterized protein LOC110374045 isoform X2	-2.180726472	0.031563067	-4.549831861	0.000170339	NA	NA
LOC110374045	uncharacterized protein LOC110374045 isoform X3	-2.180726472	0.031563067	-4.549831861	0.000170339	NA	NA
LOC110377344	AMP deaminase 2-like	-2.400999399	0.001514806	-3.162587353	0.000174759	NA	NA
LOC110374048	NA	-2.656679472	0.000327654	-6.919279343	0.000174759	NA	NA
LOC110377585	enkurin	-3.802478815	0.000165488	-4.626321752	0.000174759	NA	NA
LOC110374201	forkhead box protein A2-B-like	-1.740758138	0.032011281	-3.245024927	0.000178232	NA	NA
LOC110371186	PRKCA-binding protein isoform X3	-1.483459358	0.007734381	-2.176240753	0.000179178	NA	NA
LOC110371186	PRKCA-binding protein isoform X2	-1.483459358	0.007734381	-2.176240753	0.000179178	NA	NA
LOC110371186	PRKCA-binding protein isoform X4	-1.483459358	0.007734381	-2.176240753	0.000179178	NA	NA
LOC110371186	PRKCA-binding protein isoform X1	-1.483459358	0.007734381	-2.176240753	0.000179178	NA	NA
LOC110377567	uncharacterized protein LOC110377567	NA	NA	-1.722258608	0.000180582	NA	NA
LOC110373962	toll-like receptor 3	-2.57814149	9.41E-05	-2.722001306	0.000182759	NA	NA

LOC110378794	E3 ubiquitin-protein ligase MARCH1-like isoform X1	-2.875914861	0.000818449	-7.084202507	0.000183508	NA	NA
LOC110378794	E3 ubiquitin-protein ligase MARCH1-like isoform X3	-2.875914861	0.000818449	-7.084202507	0.000183508	NA	NA
LOC110378794	E3 ubiquitin-protein ligase MARCH1-like isoform X2	-2.875914861	0.000818449	-7.084202507	0.000183508	NA	NA
LOC110378794	E3 ubiquitin-protein ligase MARCH1-like isoform X5	-2.875914861	0.000818449	-7.084202507	0.000183508	NA	NA
LOC110378794	E3 ubiquitin-protein ligase MARCH1-like isoform X4	-2.875914861	0.000818449	-7.084202507	0.000183508	NA	NA
LOC110371257	ATP-dependent DNA helicase 2 subunit 1	NA	NA	-1.423423797	0.000186564	NA	NA
LOC110379534	uncharacterized protein LOC110379534	-2.675657061	0.00050203	-6.933849614	0.000186822	NA	NA
LOC110376722	uncharacterized protein LOC110376722 isoform X1	NA	NA	-4.257907814	0.000186972	NA	NA
LOC110376722	uncharacterized protein LOC110376722 isoform X3	NA	NA	-4.257907814	0.000186972	NA	NA
LOC110376722	uncharacterized protein LOC110376722 isoform X2	NA	NA	-4.257907814	0.000186972	NA	NA
LOC110378110	carboxypeptidase B-like	-1.526431367	0.009701842	-2.303126685	0.000187651	NA	NA

LOC110377988	ommochrome-binding protein-like	-1.590330203	0.016110257	-2.437008834	0.000187653	NA	NA
LOC110379467	transmembrane channel-like protein 2 isoform X1	-2.676527578	0.007057568	-4.925690436	0.000194696	NA	NA
LOC110379467	transmembrane channel-like protein 3 isoform X2	-2.676527578	0.007057568	-4.925690436	0.000194696	NA	NA
LOC110380980	uncharacterized protein LOC110380980 isoform X3	-2.37425453	0.001012766	-6.82564578	0.000206008	NA	NA
LOC110380980	uncharacterized protein LOC110380980 isoform X2	-2.37425453	0.001012766	-6.82564578	0.000206008	NA	NA
LOC110380980	uncharacterized protein LOC110380980 isoform X1	-2.37425453	0.001012766	-6.82564578	0.000206008	NA	NA
LOC110377386	putative nuclease HARBI1	NA	NA	-4.072075103	0.000207858	NA	NA
LOC110379683	uncharacterized protein LOC110379683	NA	NA	-7.194941468	0.000208549	NA	NA
LOC110374028	uncharacterized protein LOC110374028	-2.92704835	0.000168823	-6.898416479	0.000209545	NA	NA
LOC110374297	uncharacterized protein LOC110374297	-2.716239425	0.000166941	-6.809596071	0.000212312	NA	NA
LOC110374300	uncharacterized protein LOC110374300	-2.470368794	0.005003601	-5.304391646	0.000216439	NA	NA
LOC110371823	uncharacterized protein LOC110371823	-2.688675135	0.007774836	-5.580136124	0.000218928	NA	NA
LOC110375117	cilia- and flagella-associated protein 57 isoform X1	-1.228521931	0.013044917	-1.832847905	0.000221943	NA	NA

LOC110375117	cilia- and flagella-associated protein 57 isoform X3	-1.228521931	0.013044917	-1.832847905	0.000221943	NA	NA
LOC110375117	cilia- and flagella-associated protein 57 isoform X2	-1.228521931	0.013044917	-1.832847905	0.000221943	NA	NA
LOC110376148	oocyte zinc finger protein XlCOF6-like	NA	NA	1.13640591	0.000222058	NA	NA
LOC110381364	uncharacterized protein LOC110381364	-2.468432521	0.013549563	-4.306805207	0.00022521	NA	NA
LOC110374130	uncharacterized protein LOC110374130 isoform X1	-2.354598089	0.016985424	-4.458464067	0.000225915	NA	NA
LOC110374130	uncharacterized protein LOC110374130 isoform X2	-2.354598089	0.016985424	-4.458464067	0.000225915	NA	NA
LOC110374103	NA	NA	NA	-5.916713859	0.000226962	NA	NA
LOC110370469	kinesin-like protein KIF18A isoform X2	-1.940535034	0.003064334	-2.587044602	0.000226962	NA	NA
LOC110370469	kinesin-like protein KIF18A isoform X1	-1.940535034	0.003064334	-2.587044602	0.000226962	NA	NA
LOC110370469	kinesin-like protein KIF18A isoform X3	-1.940535034	0.003064334	-2.587044602	0.000226962	NA	NA
LOC110370469	kinesin-like protein KIF18A isoform X4	-1.940535034	0.003064334	-2.587044602	0.000226962	NA	NA
LOC110370302	gamma-glutamylcyclotransferase-like	-2.094076516	0.022977266	-3.87448509	0.000227796	NA	NA

LOC110381143	uncharacterized protein LOC110381143 isoform X3	-3.105553189	7.88E-05	-6.864353394	0.00022824	NA	NA
LOC110381143	uncharacterized protein LOC110381143 isoform X1	-3.105553189	7.88E-05	-6.864353394	0.00022824	NA	NA
LOC110381143	uncharacterized protein LOC110381143 isoform X4	-3.105553189	7.88E-05	-6.864353394	0.00022824	NA	NA
LOC110381143	uncharacterized protein LOC110381143 isoform X2	-3.105553189	7.88E-05	-6.864353394	0.00022824	NA	NA
LOC110375010	calcyphosin-like protein isoform X2	-1.509326851	0.012142462	-2.243763033	0.000229638	NA	NA
LOC110375010	calcyphosin-like protein isoform X1	-1.509326851	0.012142462	-2.243763033	0.000229638	NA	NA
LOC110375010	calcyphosin-like protein isoform X3	-1.509326851	0.012142462	-2.243763033	0.000229638	NA	NA
LOC110375010	calcyphosin-like protein isoform X4	-1.509326851	0.012142462	-2.243763033	0.000229638	NA	NA
LOC110379533	uncharacterized protein LOC110379533 isoform X1	-1.869646768	0.018327289	-2.882620224	0.000230135	NA	NA
LOC110379533	uncharacterized protein LOC110379533 isoform X2	-1.869646768	0.018327289	-2.882620224	0.000230135	NA	NA
LOC110370328	LOW QUALITY PROTEIN: lipase member H-B-like	-2.021584524	0.038390372	-4.690329562	0.000233167	NA	NA

LOC110378865	uncharacterized protein LOC110378865	-3.271143953	0.013211468	-7.526776031	0.000236703	NA	NA
LOC110376807	tetratricopeptide repeat protein 25-like	-3.214499631	0.001181531	-5.532100018	0.000238244	NA	NA
LOC110379072	monocarboxylate transporter 14-like	NA	NA	1.039646813	0.000241218	NA	NA
LOC110376878	WD repeat-containing protein on Y chromosome-like	-2.032938886	0.008798319	-3.177081438	0.000245882	NA	NA
LOC110377862	organic cation transporter protein-like isoform X1	-3.10845467	0.001041283	-4.734911172	0.000250676	NA	NA
LOC110377862	organic cation transporter protein-like isoform X2	-3.10845467	0.001041283	-4.734911172	0.000250676	NA	NA
LOC110382489	secretory phospholipase A2 receptor-like, partial	NA	NA	-2.021608179	0.000251875	NA	NA
LOC110372853	uncharacterized protein LOC110372853	NA	NA	-7.490210646	0.00025529	NA	NA
LOC110384271	tubulin polyglutamylase TTLL13-like isoform X2	-1.372789251	0.000261753	-1.459804657	0.00026331	NA	NA
LOC110384271	tubulin polyglutamylase TTLL13-like isoform X1	-1.372789251	0.000261753	-1.459804657	0.00026331	NA	NA
LOC110372567	sulfotransferase 1C4-like	-1.923288454	0.000453794	-2.160412082	0.00026331	NA	NA
LOC110380807	tachykinin-like peptides receptor 86C	NA	NA	-5.52005817	0.000265372	NA	NA
LOC110379430	coiled-coil domain-containing protein 103-like	-2.297349624	0.001431806	-6.708414596	0.000267721	NA	NA

LOC110382935	E3 ubiquitin-protein ligase RNF123-like	-2.741135526	0.029250955	-7.287932982	0.000272775	NA	NA
LOC110378682	O-acyltransferase like protein-like	NA	NA	-7.284472483	0.00027427	NA	NA
LOC110370183	intraflagellar transport protein 172 homolog	NA	NA	-3.144196594	0.000283488	NA	NA
LOC110378528	solute carrier family 12 member 6 isoform X3	NA	NA	-1.765710702	0.000283488	NA	NA
LOC110378528	solute carrier family 12 member 6 isoform X1	NA	NA	-1.765710702	0.000283488	NA	NA
LOC110378528	solute carrier family 12 member 6 isoform X4	NA	NA	-1.765710702	0.000283488	NA	NA
LOC110378528	solute carrier family 12 member 6 isoform X2	NA	NA	-1.765710702	0.000283488	NA	NA
LOC110373444	uncharacterized protein LOC110373444	NA	NA	1.615029632	0.000283488	NA	NA
LOC110377312	beta-1,4-mannosyltransferase egh	NA	NA	1.690268548	0.000283488	NA	NA
LOC110375148	cilia- and flagella-associated protein 161-like	-2.688716991	0.02888763	-7.216060852	0.000283488	NA	NA
LOC110378379	uncharacterized protein LOC110378379	-1.802771161	0.042113702	-6.807585551	0.000284036	NA	NA
LOC110373537	uncharacterized protein LOC110373537 isoform X3	NA	NA	1.718958602	0.000290715	NA	NA

LOC110373537	uncharacterized protein LOC110373537 isoform X1	NA	NA	1.718958602	0.000290715	NA	NA
LOC110373537	uncharacterized protein LOC110373537 isoform X2	NA	NA	1.718958602	0.000290715	NA	NA
LOC110370886	protein ABHD11-like	-2.820731514	0.000225502	-6.718886388	0.00029955	NA	NA
LOC110373185	mitochondrial 2-oxoglutarate/malate carrier protein-like	-3.221569898	0.000116243	-6.817369699	0.00029955	NA	NA
LOC110381368	uncharacterized protein LOC110381368	-1.842530097	0.045871971	-3.718445885	0.000299867	NA	NA
LOC110372434	L-lactate dehydrogenase-like	-2.729125844	0.011697484	-4.990157869	0.000300814	NA	NA
LOC110373683	myc proto-oncogene protein	NA	NA	1.561264821	0.000304688	NA	NA
LOC110381500	uncharacterized protein LOC110381500	-2.413271743	0.010177731	-6.929337059	0.000310047	NA	NA
LOC110383834	protein scarlet-like	NA	NA	-2.051440048	0.000312428	NA	NA
LOC110384679	trypsin epsilon-like	NA	NA	-7.199919895	0.0003163	NA	NA
LOC110380816	IQ and ubiquitin-like domain-containing protein	-3.437737624	0.000896676	-5.510235173	0.0003163	NA	NA
LOC110372343	uncharacterized protein LOC110372343 isoform X1	NA	NA	-7.131759507	0.000317587	NA	NA
LOC110372343	uncharacterized protein LOC110372343 isoform X2	NA	NA	-7.131759507	0.000317587	NA	NA

LOC110370171	lipase 3-like	NA	NA	-1.693371904	0.000323823	NA	NA
LOC110372339	uncharacterized protein LOC110372339	-2.626252887	0.048460022	-7.287197753	0.000323823	NA	NA
LOC110381753	uncharacterized protein LOC110381753 isoform X2	-1.933999455	0.000491423	-2.145468471	0.000326566	NA	NA
LOC110381753	uncharacterized protein LOC110381753 isoform X1	-1.933999455	0.000491423	-2.145468471	0.000326566	NA	NA
LOC110384645	uncharacterized protein LOC110384645	-2.149355394	0.016843147	-3.559963758	0.000326566	NA	NA
LOC110377030	NA	-2.88785325	0.000545031	-6.779001279	0.000331773	NA	NA
LOC110374482	NA	NA	NA	2.375241791	0.000332675	NA	NA
LOC110372077	probable RNA-binding protein 46	-2.003368601	0.040545955	-3.756187441	0.000332675	NA	NA
LOC110378996	uncharacterized protein LOC110378996	-3.577271366	0.000399985	-5.389974769	0.000332675	NA	NA
LOC110379268	uncharacterized protein LOC110379268	-3.968326406	0.000998843	-5.926571831	0.000334397	NA	NA
LOC110380740	uncharacterized protein LOC110380740	NA	NA	-7.203301329	0.000351815	NA	NA
LOC110372342	uncharacterized protein LOC110372342	-4.069114024	0.003669423	-7.3519384	0.000373684	NA	NA
LOC110372478	protein ABHD11-like	NA	NA	-7.452374277	0.000373696	NA	NA
LOC110380515	uncharacterized protein LOC110380515	-2.976145851	0.024522778	-7.255987223	0.000373696	NA	NA
LOC110379029	uncharacterized protein LOC110379029	NA	NA	1.154302622	0.000375854	NA	NA

LOC110373217	uncharacterized protein LOC110373217	NA	NA	-1.446172252	0.000376129	NA	NA
LOC110371299	NA	-2.600046265	0.007214916	-5.274175721	0.000379481	NA	NA
LOC110383998	cytochrome b5-related protein-like	NA	NA	-1.15513805	0.000383314	NA	NA
LOC110377859	uncharacterized protein LOC110377859	NA	NA	-1.634004017	0.000385696	NA	NA
LOC110377994	uncharacterized protein LOC110377994	-2.63446159	0.022251817	-5.022296763	0.00039072	NA	NA
LOC110376582	uncharacterized protein LOC110376582	-1.940678092	0.016449545	-3.178347985	0.000391488	NA	NA
LOC110378554	protein rolling stone-like	NA	NA	1.792889842	0.000393102	NA	NA
LOC110379822	uncharacterized protein LOC110379822	-3.440764964	0.008347184	-7.186925515	0.000393102	NA	NA
LOC110373947	uncharacterized protein LOC110373947	-2.176439946	0.005493001	-6.590448403	0.000410143	NA	NA
LOC110373646	E3 ubiquitin-protein ligase siah2-like	NA	NA	1.095712273	0.000411437	NA	NA
LOC110382084	uncharacterized protein LOC110382084	NA	NA	-1.812697719	0.000412443	NA	NA
LOC110377813	phospholipid phosphatase 5-like	NA	NA	-7.207226262	0.000416218	NA	NA
LOC110375049	leucine-rich repeat-containing protein 71-like	-3.021192322	0.000375988	-6.693022623	0.000416218	NA	NA
LOC110382757	UDP-glucuronosyltransferase 2B19-like	NA	NA	1.260795965	0.000420862	NA	NA
LOC110379855	esterase FE4-like	NA	NA	-20.25480713	0.000421671	NA	NA

LOC110378266	scavenger receptor class B member 1-like	-4.189918785	9.60E-05	-4.193652344	0.00044786	NA	NA
LOC110375834	cytosolic carboxypeptidase 4-like isoform X4	-1.204645323	0.006096614	-1.545574184	0.000448444	NA	NA
LOC110375834	cytosolic carboxypeptidase 4-like isoform X7	-1.204645323	0.006096614	-1.545574184	0.000448444	NA	NA
LOC110375834	cytosolic carboxypeptidase 4-like isoform X2	-1.204645323	0.006096614	-1.545574184	0.000448444	NA	NA
LOC110375834	cytosolic carboxypeptidase 4-like isoform X3	-1.204645323	0.006096614	-1.545574184	0.000448444	NA	NA
LOC110375834	cytosolic carboxypeptidase 4-like isoform X6	-1.204645323	0.006096614	-1.545574184	0.000448444	NA	NA
LOC110375834	cytosolic carboxypeptidase 4-like isoform X1	-1.204645323	0.006096614	-1.545574184	0.000448444	NA	NA
LOC110375834	cytosolic carboxypeptidase 4-like isoform X5	-1.204645323	0.006096614	-1.545574184	0.000448444	NA	NA
LOC110375834	cytosolic carboxypeptidase 1-like isoform X8	-1.204645323	0.006096614	-1.545574184	0.000448444	NA	NA
LOC110374677	uncharacterized protein LOC110374677	NA	NA	-4.598987997	0.000449423	NA	NA
LOC110378992	peripherin-2-like	-2.208721883	0.009721077	-6.630281165	0.000455249	NA	NA

LOC110372848	uncharacterized protein LOC110372848	-3.057001461	0.023771319	-7.190553365	0.000462324	NA	NA
LOC110376569	uncharacterized protein LOC110376569	-1.965050214	0.03885833	-3.846375162	0.000462997	NA	NA
LOC110378230	uncharacterized protein LOC110378230 isoform X3	-2.043082926	0.004863334	-2.798996645	0.000462997	NA	NA
LOC110378230	sodium-dependent nutrient amino acid transporter 1-like isoform X1	-2.043082926	0.004863334	-2.798996645	0.000462997	NA	NA
LOC110378230	sodium-dependent nutrient amino acid transporter 1-like isoform X2	-2.043082926	0.004863334	-2.798996645	0.000462997	NA	NA
LOC110383811	SHC-transforming protein 1	NA	NA	1.036828732	0.000467064	NA	NA
LOC110382956	uncharacterized transporter slc-17.2-like	NA	NA	1.100112398	0.000472472	NA	NA
LOC110381762	uncharacterized protein LOC110381762, partial	NA	NA	-4.82865703	0.000473612	NA	NA
LOC110374325	NA	NA	NA	-2.283659755	0.000473612	NA	NA
LOC110376169	synaptic vesicle glycoprotein 2B-like isoform X2	NA	NA	1.103482982	0.000476216	NA	NA
LOC110376169	synaptic vesicle glycoprotein 2B-like isoform X1	NA	NA	1.103482982	0.000476216	NA	NA
LOC110377583	NA	-3.152255442	0.024956287	-7.275451623	0.000476454	NA	NA

LOC110370999	proton-coupled amino acid transporter-like protein pathetic	NA	NA	1.449458692	0.000478102	NA	NA
LOC110381035	proteasome subunit alpha type-7-1-like	-3.00348744	0.024660345	-7.131291009	0.000480911	NA	NA
LOC110376962	uncharacterized protein LOC110376962	-2.551535818	0.043914759	-6.955549228	0.000487124	NA	NA
LOC110379645	uncharacterized protein LOC110379645	NA	NA	-7.153433353	0.000490648	NA	NA
LOC110379685	adenylate kinase isoenzyme 1-like isoform X2	-3.363887458	0.001210494	-5.333356991	0.000492291	NA	NA
LOC110379685	adenylate kinase isoenzyme 1-like isoform X1	-3.363887458	0.001210494	-5.333356991	0.000492291	NA	NA
LOC110377269	facilitated trehalose transporter Tret1-like	-1.405704966	0.001043696	-1.562462871	0.000495374	NA	NA
LOC110371768	uncharacterized protein LOC110371768 isoform X1	-2.188007972	0.048176497	-5.325239984	0.000500763	NA	NA
LOC110371768	uncharacterized protein LOC110371768 isoform X2	-2.188007972	0.048176497	-5.325239984	0.000500763	NA	NA
LOC110377045	trichohyalin-like	-2.012254318	0.005792115	-2.745182348	0.000505849	NA	NA
LOC110370591	uncharacterized protein LOC110370591 isoform X3	NA	NA	-2.865191546	0.000507891	NA	NA

LOC110370591	uncharacterized protein LOC110370591 isoform X2	NA	NA	-2.865191546	0.000507891	NA	NA
LOC110370591	uncharacterized protein LOC110370591 isoform X1	NA	NA	-2.865191546	0.000507891	NA	NA
LOC110375991	embryonic polarity protein dorsal-like	NA	NA	1.49680572	0.000517516	NA	NA
LOC110371317	vegetative cell wall protein gp1-like	NA	NA	1.285547285	0.000518727	NA	NA
LOC110379219	uncharacterized protein LOC110379219 isoform X3	-2.82456337	0.000746703	-6.571459803	0.000518941	NA	NA
LOC110379219	uncharacterized protein LOC110379219 isoform X2	-2.82456337	0.000746703	-6.571459803	0.000518941	NA	NA
LOC110379219	uncharacterized protein LOC110379219 isoform X4	-2.82456337	0.000746703	-6.571459803	0.000518941	NA	NA
LOC110379219	uncharacterized protein LOC110379219 isoform X1	-2.82456337	0.000746703	-6.571459803	0.000518941	NA	NA
LOC110378352	uncharacterized protein LOC110378352	-3.02126611	0.00096616	-6.68615446	0.000526846	NA	NA
LOC110378116	carboxypeptidase B-like isoform X2	-2.995098263	0.000491423	-6.590125675	0.000531218	NA	NA
LOC110378116	carboxypeptidase B-like isoform X1	-2.995098263	0.000491423	-6.590125675	0.000531218	NA	NA

LOC110376851	uncharacterized protein LOC110376851	-3.196932147	0.004700741	-4.939277485	0.000539347	NA	NA
LOC110381280	dynein heavy chain 2, axonemal	-1.466297066	0.037111292	-2.400582045	0.000557358	NA	NA
LOC110374953	transmembrane protease serine 11E-like	-2.961183689	0.000486727	-6.549858724	0.0005591	NA	NA
LOC110377152	uncharacterized protein LOC110377152	1.127988611	0.019978462	1.586873119	0.000560162	NA	NA
LOC110371321	coiled-coil domain-containing protein 96	-2.844597365	0.007727758	-4.43739618	0.000560162	NA	NA
LOC110379373	ATP synthase subunit gamma, mitochondrial-like isoform X2	-2.307517295	0.016802499	-6.66424634	0.000571414	NA	NA
LOC110379373	ATP synthase subunit gamma, mitochondrial-like isoform X1	-2.307517295	0.016802499	-6.66424634	0.000571414	NA	NA
LOC110374256	uncharacterized protein LOC110374256	-2.789402581	0.001597536	-6.598835849	0.000571414	NA	NA
LOC110377515	uncharacterized protein LOC110377515	NA	NA	-1.822018322	0.000582005	NA	NA
LOC110370398	alpha-(1,3)-fucosyltransferase C-like	NA	NA	-6.917488381	0.000601726	NA	NA
LOC110380866	LOW QUALITY PROTEIN: RNA-binding protein Musashi homolog Rbp6-like	NA	NA	-2.472996685	0.00060264	NA	NA
LOC110376691	alpha-N-acetylgalactosaminidase isoform X1	-1.526795089	0.001012766	-1.688532297	0.000604293	NA	NA

LOC110376691	alpha-N-acetylgalactosaminidase isoform X2	-1.526795089	0.001012766	-1.688532297	0.000604293	NA	NA
LOC110379291	uncharacterized protein LOC110379291 isoform X2	-2.566241844	0.000638302	-2.809120242	0.000608743	NA	NA
LOC110379291	uncharacterized protein LOC110379291 isoform X1	-2.566241844	0.000638302	-2.809120242	0.000608743	NA	NA
LOC110384123	uncharacterized protein LOC110384123	-3.2506157	0.000273431	-6.561640731	0.000611814	NA	NA
LOC110378577	uncharacterized protein LOC110378577	-2.765689273	0.001274377	-6.525776797	0.000612819	NA	NA
LOC110378709	uncharacterized protein C4orf22 homolog	-3.376112401	0.001232806	-4.347318573	0.000618964	NA	NA
LOC110380166	uncharacterized protein LOC110380166	NA	NA	-6.946028242	0.000631425	NA	NA
LOC110377853	probable ATP-dependent RNA helicase DHX35	NA	NA	1.075636594	0.000631425	NA	NA
LOC110369746	cytochrome b5 domain-containing protein 1	-2.35648673	0.024369372	-5.181962445	0.000631425	NA	NA
LOC110380744	sodium- and chloride-dependent transporter XTRP3 isoform X3	-3.434449381	0.000777198	-4.079096136	0.000635468	NA	NA
LOC110380744	sodium- and chloride-dependent transporter XTRP3 isoform X1	-3.434449381	0.000777198	-4.079096136	0.000635468	NA	NA

LOC110380744	sodium- and chloride-dependent transporter XTRP3 isoform X2	-3.434449381	0.000777198	-4.079096136	0.000635468	NA	NA
LOC110374108	MICOS complex subunit MIC19-like	-2.067978302	0.043731732	-4.457196445	0.000637278	NA	NA
LOC110384397	parkin coregulated gene protein homolog	-2.626820703	0.017172336	-4.736900203	0.000637302	NA	NA
LOC110375425	uncharacterized protein LOC110375425	-2.958700117	0.035464583	-7.069318002	0.000638031	NA	NA
LOC110372927	iron-regulated transcriptional activator AFT2-like	NA	NA	1.345470725	0.000647133	NA	NA
LOC110382989	uncharacterized protein LOC110382989	-2.921786265	0.000858346	-6.520344865	0.000647157	NA	NA
LOC110370608	uncharacterized protein LOC110370608	NA	NA	-4.504484486	0.000656722	NA	NA
LOC110382503	H/ACA ribonucleoprotein complex subunit 4	NA	NA	1.067684959	0.000656722	NA	NA
LOC110377649	uncharacterized protein LOC110377649	NA	NA	1.048488716	0.000664397	NA	NA
LOC110378849	probable ATP-dependent RNA helicase YTHDC2	NA	NA	1.274558438	0.000676833	NA	NA
LOC110381134	ATP synthase-coupling factor 6, mitochondrial-like	-3.234397526	0.019237467	-7.003851786	0.000679784	NA	NA
LOC110378800	attacin-E-like	NA	NA	1.275586889	0.000684915	NA	NA
LOC110379443	uncharacterized protein LOC110379443	-3.03221815	0.004032735	-5.229720886	0.000685502	NA	NA

LOC110379340	uncharacterized protein LOC110379340	NA	NA	-6.990506358	0.000697184	NA	NA
LOC110372184	uncharacterized protein LOC110372184	NA	NA	-4.981012897	0.000697184	NA	NA
LOC110375849	uncharacterized protein LOC110375849	NA	NA	1.36278039	0.000700246	NA	NA
LOC110378620	actin-like isoform X2	-2.999882708	0.002802581	-6.672331288	0.00071435	NA	NA
LOC110378620	actin-85C-like isoform X1	-2.999882708	0.002802581	-6.672331288	0.00071435	NA	NA
LOC110379760	homeotic protein antennapedia	-1.708698935	0.000129783	-1.637520786	0.000718045	NA	NA
LOC110373092	uncharacterized protein LOC110373092	-2.367840596	0.022455134	-5.115152517	0.000718045	NA	NA
LOC110378730	uncharacterized protein LOC110378730	-1.521188157	0.003958666	-1.84699589	0.000720377	NA	NA
LOC110381427	long-chain fatty acid transport protein 4-like	NA	NA	-1.16208079	0.00073265	NA	NA
LOC110376046	LOW QUALITY PROTEIN: nucleolar protein 12-like	NA	NA	1.052864761	0.00073265	NA	NA
LOC110370392	leucine-rich repeat extensin-like protein 3	-2.54693468	0.005706763	-6.511083813	0.000736017	NA	NA
LOC110382761	UDP-glucuronosyltransferase 2B15-like	NA	NA	1.100167184	0.000738322	NA	NA
LOC110380709	LOW QUALITY PROTEIN: lachesin-like	-1.793961713	2.83E-07	-1.333602469	0.000743097	NA	NA
LOC110372648	uncharacterized protein LOC110372648	-3.545264017	0.000399715	-6.630346468	0.000743097	NA	NA
LOC110375346	microvitellogenin-like	NA	NA	1.843246173	0.000745971	NA	NA

LOC110384292	Krueppel-like factor 9	-1.94132662	0.01330112	-2.773404369	0.00074716	NA	NA
LOC110375339	uncharacterized protein LOC110375339	-2.709156312	0.008570988	-4.22066628	0.00075368	NA	NA
LOC110378377	uncharacterized protein LOC110378377	NA	NA	-6.865861475	0.000767876	NA	NA
LOC110377753	uncharacterized protein LOC110377753 isoform X3	-2.292866623	0.000811964	-2.479233994	0.000769267	NA	NA
LOC110377753	uncharacterized protein LOC110377753 isoform X6	-2.292866623	0.000811964	-2.479233994	0.000769267	NA	NA
LOC110377753	uncharacterized protein LOC110377753 isoform X4	-2.292866623	0.000811964	-2.479233994	0.000769267	NA	NA
LOC110377753	uncharacterized protein LOC110377753 isoform X5	-2.292866623	0.000811964	-2.479233994	0.000769267	NA	NA
LOC110377753	uncharacterized protein LOC110377753 isoform X2	-2.292866623	0.000811964	-2.479233994	0.000769267	NA	NA
LOC110377753	uncharacterized protein LOC110377753 isoform X1	-2.292866623	0.000811964	-2.479233994	0.000769267	NA	NA
LOC110376621	uncharacterized protein LOC110376621	-5.287823759	5.84E-05	-4.065687983	0.000769267	NA	NA
LOC110377314	uncharacterized protein LOC110377314	NA	NA	1.123819956	0.000773211	NA	NA
LOC110379440	protein takeout	-1.454190914	0.004737943	-1.791814372	0.000774795	NA	NA

LOC110374098	uncharacterized protein LOC110374098	-1.131823629	0.026176691	-1.6831487	0.000775441	NA	NA
LOC110379463	uncharacterized protein LOC110379463 isoform X2	-2.121443302	0.042283585	-4.430296561	0.000781429	NA	NA
LOC110379463	uncharacterized protein LOC110379463 isoform X1	-2.121443302	0.042283585	-4.430296561	0.000781429	NA	NA
LOC110379463	uncharacterized protein LOC110379463 isoform X3	-2.121443302	0.042283585	-4.430296561	0.000781429	NA	NA
LOC110373238	nucleoside diphosphate-linked moiety X motif 8-like	-2.664185089	0.012828969	-4.584304865	0.000800143	NA	NA
LOC110374696	uncharacterized protein LOC110374696	-2.865480311	0.003177616	-6.554101607	0.000800143	NA	NA
LOC110381340	uncharacterized protein LOC110381340 isoform X2	-3.353385046	0.001514034	-4.128659753	0.000803814	NA	NA
LOC110381340	uncharacterized protein LOC110381340 isoform X1	-3.353385046	0.001514034	-4.128659753	0.000803814	NA	NA
LOC110379104	uncharacterized protein LOC110379104 isoform X2	-2.783295784	0.010015571	-5.196786273	0.000804799	NA	NA
LOC110379104	uncharacterized protein LOC110379104 isoform X1	-2.783295784	0.010015571	-5.196786273	0.000804799	NA	NA

LOC110380099	uncharacterized protein LOC110380099 isoform X3	NA	NA	-6.946511577	0.000816056	NA	NA
LOC110380099	uncharacterized protein LOC110380099 isoform X2	NA	NA	-6.946511577	0.000816056	NA	NA
LOC110380099	uncharacterized protein LOC110380099 isoform X1	NA	NA	-6.946511577	0.000816056	NA	NA
LOC110372484	uncharacterized protein LOC110372484 isoform X2	NA	NA	1.324690637	0.000816717	NA	NA
LOC110372484	uncharacterized protein LOC110372484 isoform X1	NA	NA	1.324690637	0.000816717	NA	NA
LOC110381359	pre-mRNA 3\\' end processing protein WDR33-like	NA	NA	-6.293968683	0.000820219	NA	NA
LOC110378477	cytochrome P450 9e2-like	NA	NA	1.535166179	0.000830082	NA	NA
LOC110380333	multidrug resistance-associated protein 4-like	NA	NA	1.123904533	0.000831487	NA	NA
LOC110382811	zinc finger protein GLIS2-like	NA	NA	-1.49603699	0.000852849	NA	NA
LOC110373913	dual specificity protein phosphatase 1B-like	-2.753773249	0.010644468	-5.162956634	0.000854878	NA	NA
LOC110382326	uncharacterized protein LOC110382326 isoform X2	NA	NA	-6.54918934	0.00087717	NA	NA

LOC110382326	uncharacterized protein LOC110382326 isoform X1	NA	NA	-6.54918934	0.00087717	NA	NA
LOC110371421	uncharacterized protein LOC110371421	-2.167304574	0.011156814	-6.311206253	0.00087717	NA	NA
LOC110378275	proton-coupled amino acid transporter-like protein pathetic	-2.783769425	0.001720939	-6.385202521	0.00087717	NA	NA
LOC110384360	multidrug resistance-associated protein 1-like	NA	NA	1.347889827	0.000880273	NA	NA
LOC110370865	protein ABHD11-like	NA	NA	-6.579846861	0.000883152	NA	NA
LOC110371374	facilitated trehalose transporter Tret1-like isoform X1	NA	NA	1.619329938	0.000883152	NA	NA
LOC110371374	facilitated trehalose transporter Tret1-like isoform X2	NA	NA	1.619329938	0.000883152	NA	NA
LOC110381127	G2/mitotic-specific cyclin-B3 isoform X1	-1.430363227	0.007675192	-1.83240293	0.000883152	NA	NA
LOC110381127	G2/mitotic-specific cyclin-B3 isoform X2	-1.430363227	0.007675192	-1.83240293	0.000883152	NA	NA
LOC110374266	uncharacterized protein LOC110374266	-2.318521144	0.00834448	-6.34504421	0.000886527	NA	NA
LOC110370849	DNA repair and recombination protein RAD54-like	NA	NA	-1.312747065	0.000887766	NA	NA
LOC110371015	NA	NA	NA	-1.396311466	0.000890153	NA	NA
LOC110375592	luciferin 4-monooxygenase-like	NA	NA	-1.207980353	0.000890153	NA	NA

LOC110370904	dnaJ homolog subfamily B member 13-like	-2.149227035	0.024706972	-6.412329817	0.000890153	NA	NA
LOC110384677	trypsin inhibitor-like	NA	NA	-1.797906508	0.00089408	NA	NA
LOC110372270	uncharacterized protein LOC110372270	-2.013502809	0.046713355	-4.907085258	0.00089408	NA	NA
LOC110377518	uncharacterized protein LOC110377518	NA	NA	1.088889534	0.000932924	NA	NA
LOC110382234	nuclear factor NF-kappa-B p100 subunit-like isoform X3	-2.38539022	0.027297596	-5.075599354	0.000932924	NA	NA
LOC110382234	uncharacterized protein LOC110382234 isoform X1	-2.38539022	0.027297596	-5.075599354	0.000932924	NA	NA
LOC110382234	potassium channel KOR1-like isoform X2	-2.38539022	0.027297596	-5.075599354	0.000932924	NA	NA
LOC110382234	uncharacterized protein LOC110382234 isoform X4	-2.38539022	0.027297596	-5.075599354	0.000932924	NA	NA
LOC110380033	NA	-2.944229241	0.001070611	-6.365990426	0.000932924	NA	NA
LOC110377394	cytochrome P450 4d2-like	NA	NA	1.514089678	0.00093785	NA	NA
LOC110383941	uncharacterized protein LOC110383941	NA	NA	-1.83167706	0.000939705	NA	NA
LOC110381883	acetylcholinesterase-like	-2.298599162	0.000177762	-2.195474606	0.000940838	NA	NA
LOC110381898	paramyosin	NA	NA	-5.161715082	0.000956396	NA	NA
LOC110371462	WD repeat-containing protein 43	NA	NA	1.13772832	0.000965317	NA	NA
LOC110379478	dynein light chain roadblock-type 2-like	-2.949664182	0.002516639	-6.45832814	0.000979054	NA	NA

LOC110374370	uncharacterized protein LOC110374370	-2.303899833	0.040545955	-6.572270981	0.00098496	NA	NA
LOC110380074	renin receptor-like	-2.873305844	0.004731078	-4.379536795	0.000987404	NA	NA
LOC110378798	uncharacterized protein LOC110378798	-2.172478346	0.038628215	-3.862161628	0.000996264	NA	NA
LOC110382487	uncharacterized protein LOC110382487	-2.527736541	0.016985424	-5.032393675	0.000996264	NA	NA
LOC110379224	uncharacterized protein LOC110379224	NA	NA	-6.80191576	0.000996564	NA	NA
LOC110384394	androgen-dependent TFPI-regulating protein-like	NA	NA	-1.26236968	0.000996564	NA	NA
LOC110374682	methyltransferase-like protein 6	NA	NA	-1.793903718	0.001018751	NA	NA
LOC110384665	uncharacterized protein LOC110384665	NA	NA	-4.804677903	0.001029845	NA	NA
LOC110383084	tubulin beta chain	NA	NA	-6.404895708	0.001034254	NA	NA
LOC110379606	putative inorganic phosphate cotransporter	NA	NA	1.051781185	0.001034254	NA	NA
LOC110379720	putative inorganic phosphate cotransporter	-2.433631816	0.008528167	-6.331323833	0.001034254	NA	NA
LOC110373237	nucleoside diphosphate-linked moiety X motif 8-like	-2.73000351	0.005260711	-6.41943277	0.001034254	NA	NA
LOC110380439	RNA helicase Mov10l1-like	NA	NA	1.012874994	0.001052597	NA	NA
LOC110372422	delta(24)-sterol reductase-like	NA	NA	-1.074822814	0.001060807	NA	NA

LOC110370435	leucine-rich repeat-containing protein 74A-like	NA	NA	-7.054994059	0.001071177	NA	NA
LOC110380473	carbonic anhydrase 1	-1.695377654	1.84E-08	-1.128968282	0.001084279	NA	NA
LOC110371954	neuroguidin	NA	NA	1.332671698	0.001095969	NA	NA
LOC110374803	uncharacterized protein LOC110374803	-2.609588761	0.003427641	-3.273830052	0.001106762	NA	NA
LOC110384681	trypsin epsilon-like	-3.108225291	0.000847555	-6.314089257	0.001107823	NA	NA
LOC110382251	cilia- and flagella-associated protein 43	NA	NA	-3.914371612	0.001112631	NA	NA
LOC110384415	transmembrane protein 136-like	NA	NA	-4.156159853	0.001140079	NA	NA
LOC110375851	NADH dehydrogenase	-3.371945345	0.001991159	-5.048475275	0.00115288	NA	NA
LOC110381772	uncharacterized protein LOC110381772 isoform X2	NA	NA	-7.095276362	0.001159823	NA	NA
LOC110381772	uncharacterized protein LOC110381772 isoform X1	NA	NA	-7.095276362	0.001159823	NA	NA
LOC110380927	L-dopachrome tautomerase yellow-f2-like	NA	NA	-4.450643518	0.001161026	NA	NA
LOC110382855	protein mothers against dpp	NA	NA	1.217436629	0.001161026	NA	NA
LOC110373343	UDP-glucuronosyltransferase 2B1-like isoform X2	NA	NA	-1.576303116	0.001162299	NA	NA

LOC110373343	UDP-glucuronosyltransferase 2B1-like isoform X1	NA	NA	-1.576303116	0.001162299	NA	NA
LOC110376594	tubulin beta-4B chain-like	NA	NA	-5.962954894	0.001169936	NA	NA
LOC110382566	NA	-3.634532297	0.018157319	-6.890855935	0.00119191	NA	NA
LOC110378384	sodium- and chloride-dependent creatine transporter 1-like	-2.590386632	0.01656217	-4.42962801	0.001199279	NA	NA
LOC110378901	NA	-3.044147681	0.000941373	-6.252105652	0.001208846	NA	NA
LOC110372350	uncharacterized protein LOC110372350	-2.748344401	0.002829924	-6.25383721	0.001226495	NA	NA
LOC110369942	intraflagellar transport protein 20 homolog isoform X1	NA	NA	-3.629307525	0.00122757	NA	NA
LOC110369942	intraflagellar transport protein 20 homolog isoform X2	NA	NA	-3.629307525	0.00122757	NA	NA
LOC110378551	uncharacterized protein LOC110378551	NA	NA	-6.891151517	0.001267974	NA	NA
LOC110369620	uncharacterized protein LOC110369620 isoform X1	NA	NA	-5.492133942	0.001289499	NA	NA
LOC110369620	uncharacterized protein LOC110369620 isoform X2	NA	NA	-5.492133942	0.001289499	NA	NA
LOC110371798	monocarboxylate transporter 9-like	NA	NA	-1.025477987	0.001290908	NA	NA
LOC110377281	uncharacterized protein LOC110377281	-2.675708289	0.021794605	-4.589914022	0.001294227	NA	NA

LOC110373919	NA	-2.496075934	0.008230482	-6.245222116	0.001299748	NA	NA
LOC110380575	trypsin, alkaline C-like	1.327469695	0.001394423	1.403885096	0.001327208	NA	NA
LOC110378077	uncharacterized protein LOC110378077	NA	NA	-3.896570445	0.001327481	NA	NA
LOC110377218	NA	NA	NA	-6.686858664	0.001328433	NA	NA
LOC110379989	uncharacterized protein LOC110379989 isoform X2	-1.108906372	0.001685711	-1.191826134	0.001332256	NA	NA
LOC110379989	uncharacterized protein LOC110379989 isoform X1	-1.108906372	0.001685711	-1.191826134	0.001332256	NA	NA
LOC110376677	beta-1,3-galactosyltransferase 5-like	NA	NA	1.503819869	0.001332906	NA	NA
LOC110376259	uncharacterized protein LOC110376259	NA	NA	-6.911033242	0.001351369	NA	NA
LOC110384168	putative lipoyltransferase 2, mitochondrial	NA	NA	-1.350625465	0.001369789	NA	NA
LOC110375847	uncharacterized protein LOC110375847	-6.994667503	5.47E-05	-6.603707894	0.001380555	NA	NA
LOC110379353	NA	-2.602147035	0.021780725	-5.025025575	0.001403336	NA	NA
LOC110373009	probable NADH dehydrogenase	-2.470813687	0.026354876	-4.969205196	0.001404686	NA	NA
LOC110372733	uncharacterized protein LOC110372733	NA	NA	-6.745742317	0.001437904	NA	NA
LOC110383056	NA	-3.412846261	0.036896119	-6.965292713	0.001438436	NA	NA
LOC110370184	sperm-associated antigen 6-like	-2.421272583	0.008538208	-3.25401182	0.001447018	NA	NA
LOC110371283	NADH dehydrogenase	-2.620065318	0.006604742	-6.218129352	0.001455807	NA	NA

LOC110383800	lipoprotein lipase-like	1.873289526	0.000118233	1.690775984	0.001491273	NA	NA
LOC110379053	retinol dehydrogenase 12-like	NA	NA	-1.018969604	0.001497328	NA	NA
LOC110378072	protein penguin isoform X1	NA	NA	1.022119179	0.001503086	NA	NA
LOC110378072	protein penguin isoform X2	NA	NA	1.022119179	0.001503086	NA	NA
LOC110379293	mushroom body large-type Kenyon cell-specific protein 1	-2.388446312	0.00030587	-2.269903077	0.001523368	NA	NA
LOC110373706	F-box only protein 32 isoform X2	-1.248014897	0.013603282	-1.589763656	0.001527777	NA	NA
LOC110373706	F-box only protein 32 isoform X1	-1.248014897	0.013603282	-1.589763656	0.001527777	NA	NA
LOC110377204	probable ATP-dependent RNA helicase DDX43	NA	NA	1.128965019	0.001538103	NA	NA
LOC110382193	probable ATP synthase subunit g 1, mitochondrial	-2.803668703	0.004296499	-6.214615963	0.001554317	NA	NA
LOC110378195	NA	-3.457709232	0.025784188	-6.733727826	0.001554317	NA	NA
LOC110373936	uncharacterized protein LOC110373936	-2.543450289	0.004863334	-3.241531067	0.001579593	NA	NA
LOC110370223	NA	-2.567037045	0.0028306	-3.00661346	0.00158444	NA	NA
LOC110382817	proton-coupled amino acid transporter-like protein CG1139	NA	NA	-2.384400475	0.001584869	NA	NA
LOC110380903	apyrase-like	NA	NA	2.189887365	0.001584869	NA	NA
LOC110384448	uncharacterized protein LOC110384448	NA	NA	-4.743747888	0.001586404	NA	NA

LOC110379812	adhesive plaque matrix protein-like	-3.108881054	0.005080723	-4.943523126	0.001594949	NA	NA
LOC110370823	NA	-2.339275243	0.024042715	-6.232450244	0.001598797	NA	NA
LOC110372568	cilia- and flagella-associated protein 65-like	-2.893035451	0.006646842	-4.848327695	0.001598797	NA	NA
LOC110376338	uncharacterized protein LOC110376338	NA	NA	1.013315803	0.001606947	NA	NA
LOC110382875	E3 ubiquitin-protein ligase RFWD3-like	NA	NA	-1.94548953	0.00161499	NA	NA
LOC110375846	T-complex-associated testis-expressed protein 1-like	-2.842896315	0.015052683	-4.267728258	0.001619542	NA	NA
LOC110375688	uncharacterized protein LOC110375688	NA	NA	1.068439298	0.001642714	NA	NA
LOC110370081	uncharacterized protein LOC110370081	NA	NA	2.49872421	0.001665906	NA	NA
LOC110372768	uncharacterized protein LOC110372768 isoform X1	NA	NA	-1.40403588	0.001670884	NA	NA
LOC110372768	uncharacterized protein LOC110372768 isoform X2	NA	NA	-1.40403588	0.001670884	NA	NA
LOC110372865	uncharacterized protein LOC110372865	-3.223531709	0.044758765	-6.772861258	0.0017107	NA	NA
LOC110381147	uncharacterized protein LOC110381147	NA	NA	-3.91819619	0.001731535	NA	NA
LOC110375828	NA	NA	NA	2.12654445	0.001754691	NA	NA

LOC110381369	uncharacterized protein LOC110381369	-3.335924222	0.000882746	-6.159496107	0.001754691	NA	NA
LOC110370438	uncharacterized protein LOC110370438	-3.381993621	0.003292993	-4.205090568	0.001823656	NA	NA
LOC110377112	facilitated trehalose transporter Tret1-2 homolog	NA	NA	1.204758323	0.001837999	NA	NA
LOC110383241	uncharacterized protein LOC110383241	-1.065021598	0.025228609	-1.444371776	0.001850532	NA	NA
LOC110378784	sodium-independent sulfate anion transporter-like isoform X2	NA	NA	-1.176880806	0.001873755	NA	NA
LOC110378784	sodium-independent sulfate anion transporter-like isoform X1	NA	NA	-1.176880806	0.001873755	NA	NA
LOC110378276	proton-coupled amino acid transporter-like protein pathetic	NA	NA	-6.646183277	0.001880098	NA	NA
LOC110370712	myophilin	NA	NA	-1.051142905	0.00189696	NA	NA
LOC110372751	matrix metalloproteinase-25-like	-1.293937447	0.006646842	-1.535471511	0.00189696	NA	NA
LOC110381767	uncharacterized protein LOC110381767 isoform X1	NA	NA	-2.847459686	0.001911187	NA	NA
LOC110381767	uncharacterized protein LOC110381767 isoform X2	NA	NA	-2.847459686	0.001911187	NA	NA
LOC110381566	arylsulfatase I-like	NA	NA	1.73253158	0.001962541	NA	NA

LOC110379610	uncharacterized protein LOC110379610	-2.629489115	0.018096069	-4.837620039	0.001962842	NA	NA
LOC110374972	uncharacterized protein LOC110374972	NA	NA	1.123138937	0.001997279	NA	NA
LOC110374465	dynein light chain 1, axonemal	NA	NA	-6.710951827	0.00201185	NA	NA
LOC110384660	NA	NA	NA	-2.424024364	0.002015104	NA	NA
LOC110383174	protein argonaute-2-like	-2.707685904	4.25E-06	-1.976194566	0.002015104	NA	NA
LOC110374091	uncharacterized protein LOC110374091	-3.172708143	0.004104904	-6.258733516	0.00202737	NA	NA
LOC110373895	uncharacterized protein LOC110373895 isoform X2	NA	NA	-3.72339749	0.002065028	NA	NA
LOC110373895	sperm-associated antigen 17-like isoform X1	NA	NA	-3.72339749	0.002065028	NA	NA
LOC110384303	uncharacterized protein LOC110384303	NA	NA	-6.19034835	0.002073505	NA	NA
LOC110371670	acyl-CoA Delta(11) desaturase-like	-2.914186367	0.000408334	-2.750916182	0.002076376	NA	NA
LOC110378684	putative protein TPRXL	-3.32683088	0.013867743	-6.636232493	0.002076376	NA	NA
LOC110370504	uncharacterized protein LOC110370504	NA	NA	-8.890774977	0.002081233	NA	NA
LOC110372351	protein phosphatase 1 regulatory subunit 36-like	-2.543381386	0.045335553	-5.055042074	0.002099323	NA	NA
LOC110377897	uncharacterized protein LOC110377897	NA	NA	1.142404856	0.002133505	NA	NA
LOC110384106	zonadhesin-like isoform X2	-1.831050726	9.30E-07	-1.307336197	0.00214233	NA	NA

LOC110384106	zonadhesin-like isoform X1	-1.831050726	9.30E-07	-1.307336197	0.00214233	NA	NA
LOC110383053	uncharacterized protein LOC110383053	NA	NA	-2.732375021	0.002155507	NA	NA
LOC110380956	facilitated trehalose transporter Tret1-like	NA	NA	-1.117400053	0.002161644	NA	NA
LOC110376559	actin-85C-like isoform X1	-2.74105271	0.047734851	-5.266647071	0.002174009	NA	NA
LOC110376559	actin-like isoform X2	-2.74105271	0.047734851	-5.266647071	0.002174009	NA	NA
LOC110384291	centrosomal protein of 120 kDa-like	NA	NA	-1.881851688	0.002195039	NA	NA
LOC110380023	NA	NA	NA	1.064934725	0.002200977	NA	NA
LOC110372958	uncharacterized protein LOC110372958	NA	NA	-6.477480006	0.002220735	NA	NA
LOC110370279	organic cation transporter protein-like isoform X1	NA	NA	-1.353447323	0.002238207	NA	NA
LOC110370279	organic cation transporter protein-like isoform X2	NA	NA	-1.353447323	0.002238207	NA	NA
LOC110370981	organic cation transporter protein-like	-2.103355343	0.007356379	-2.536241478	0.002256181	NA	NA
LOC110370497	uncharacterized protein LOC110370497	-3.864056097	0.000811964	-3.950561647	0.002318252	NA	NA
LOC110376079	uncharacterized protein LOC110376079 isoform X2	NA	NA	-1.280902466	0.002320457	NA	NA
LOC110376079	uncharacterized protein LOC110376079 isoform X1	NA	NA	-1.280902466	0.002320457	NA	NA

LOC110379933	esterase FE4-like	NA	NA	-1.041149068	0.002343694	NA	NA
LOC110371303	adenylate cyclase type 10-like	-2.277457392	0.027687042	-6.017455664	0.002383708	NA	NA
LOC110370884	uncharacterized protein LOC110370884	-1.603060003	2.04E-07	-1.061300756	0.00249834	NA	NA
LOC110374038	cytochrome c-like	NA	NA	-3.831992647	0.002508804	NA	NA
LOC110378559	trypsin, alkaline C-like	4.749633262	0.036445354	6.432576572	0.002508804	NA	NA
LOC110378923	nucleoside diphosphate kinase 7-like	-2.97025518	0.000729019	-2.891796521	0.00256326	NA	NA
LOC110370596	dynein heavy chain 12, axonemal	-1.794237577	0.04125729	-2.694233433	0.002589128	NA	NA
LOC110381846	proteasome subunit beta type-2-like	-3.828625614	0.003634446	-5.136102355	0.002606625	NA	NA
LOC110373979	ATP-binding cassette sub-family A member 3-like	-1.840993724	0.016843147	-2.361641341	0.002736958	NA	NA
LOC110372059	innexin inx2-like	NA	NA	-2.697350757	0.002795888	NA	NA
LOC110372557	phospholipid hydroperoxide glutathione peroxidase, mitochondrial-like	NA	NA	-4.206052192	0.002828727	NA	NA
LOC110382242	phosphatidylinositol 4-phosphate 5-kinase 2-like	-2.542700293	0.014883945	-5.954861682	0.002907703	NA	NA
LOC110373321	oleosin-B3-like	NA	NA	1.048411026	0.002912233	NA	NA
LOC110383681	IQ domain-containing protein D-like	-2.198195978	0.043711431	-3.411039321	0.002918634	NA	NA
LOC110374050	venom allergen 5.01-like	NA	NA	1.144557325	0.003067517	NA	NA
LOC110370901	uncharacterized protein LOC110370901	NA	NA	1.365524929	0.003082423	NA	NA

LOC110370256	DNA fragmentation factor subunit alpha	NA	NA	-1.312246634	0.003083003	NA	NA
LOC110381457	uncharacterized protein LOC110381457	NA	NA	-3.04678789	0.003163657	NA	NA
LOC110369753	macrophage mannose receptor 1-like	-2.389147751	2.02E-06	-1.692934654	0.003168588	NA	NA
LOC110377000	uncharacterized protein LOC110377000	-2.175973222	0.016782892	-2.857926562	0.003210902	NA	NA
LOC110370187	uncharacterized protein LOC110370187	-2.942869512	0.009678097	-6.04508877	0.003212697	NA	NA
LOC110380459	phagocyte signaling-impaired protein	NA	NA	1.118959769	0.003274402	NA	NA
LOC110377409	uncharacterized protein LOC110377409 isoform X1	NA	NA	-6.349615555	0.003323259	NA	NA
LOC110377409	uncharacterized protein LOC110377409 isoform X2	NA	NA	-6.349615555	0.003323259	NA	NA
LOC110374725	replication protein A 70 kDa DNA-binding subunit	NA	NA	-1.003543163	0.003332827	NA	NA
LOC110376590	adenylate cyclase type 8-like	NA	NA	-1.501216142	0.003382771	NA	NA
LOC110374257	sodium/potassium/calcium exchanger 3 isoform X2	-2.423559962	0.007057568	-2.861004378	0.003392507	NA	NA
LOC110374257	sodium/potassium/calcium exchanger 3 isoform X3	-2.423559962	0.007057568	-2.861004378	0.003392507	NA	NA

LOC110374257	sodium/potassium/calcium exchanger 4 isoform X5	-2.423559962	0.007057568	-2.861004378	0.003392507	NA	NA
LOC110374257	sodium/potassium/calcium exchanger 3 isoform X4	-2.423559962	0.007057568	-2.861004378	0.003392507	NA	NA
LOC110374257	sodium/potassium/calcium exchanger 3 isoform X1	-2.423559962	0.007057568	-2.861004378	0.003392507	NA	NA
LOC110374792	N-terminal Xaa-Pro-Lys N-methyltransferase 1-like	-2.691174157	0.010716054	-5.891279345	0.003392507	NA	NA
LOC110374942	FUN14 domain-containing protein 2-like	NA	NA	-6.532170391	0.00340524	NA	NA
LOC110378527	cysteine and glycine-rich protein 1-like isoform X2	NA	NA	-6.313296345	0.003514583	NA	NA
LOC110378527	uncharacterized protein LOC110378527 isoform X1	NA	NA	-6.313296345	0.003514583	NA	NA
LOC110378527	cysteine and glycine-rich protein 1-like isoform X3	NA	NA	-6.313296345	0.003514583	NA	NA
LOC110374458	leucine-rich repeat-containing protein 57 isoform X2	-1.245543725	0.001421245	-1.225282571	0.003557598	NA	NA
LOC110374458	leucine-rich repeat-containing protein 40 isoform X1	-1.245543725	0.001421245	-1.225282571	0.003557598	NA	NA
LOC110381497	uncharacterized protein LOC110381497	NA	NA	-4.730713002	0.003600972	NA	NA
LOC110373534	NA	NA	NA	1.776249781	0.003619979	NA	NA

LOC110382061	zinc finger MYND domain-containing protein 10	-2.795662838	0.009426424	-5.88675124	0.003619979	NA	NA
LOC110380674	outer dense fiber protein 3-like	-2.009637232	0.006303698	-2.284044237	0.003678852	NA	NA
LOC110380554	afadin	-1.195421876	0.000376591	-1.073789884	0.003686393	NA	NA
LOC110377063	retinol dehydrogenase 14	-1.581261428	4.07E-05	-1.25347351	0.003709986	NA	NA
LOC110378713	RING finger protein 5-like	-2.667421932	0.020863225	-3.872289555	0.003773853	NA	NA
LOC110379918	coiled-coil domain-containing protein 113-like	NA	NA	-6.342340316	0.00379464	NA	NA
LOC110380755	acrosin-like	-3.100867767	0.010822455	-6.064805104	0.003799319	NA	NA
LOC110381735	uncharacterized protein LOC110381735	NA	NA	1.407790512	0.003864784	NA	NA
LOC110371777	cytochrome P450 4g15-like	NA	NA	-2.606974905	0.003906355	NA	NA
LOC110373820	alpha-tocopherol transfer protein-like	NA	NA	1.195078345	0.004092961	NA	NA
LOC110371267	NA	NA	NA	-2.668177776	0.004136515	NA	NA
LOC110377212	uncharacterized protein LOC110377212	NA	NA	1.47883898	0.00413745	NA	NA
LOC110375197	uncharacterized protein LOC110375197 isoform X2	NA	NA	-2.984129291	0.004148772	NA	NA
LOC110375197	uncharacterized protein LOC110375197 isoform X1	NA	NA	-2.984129291	0.004148772	NA	NA
LOC110375197	vicilin-like seed storage protein At2g18540 isoform X3	NA	NA	-2.984129291	0.004148772	NA	NA

LOC110380404	uncharacterized protein LOC110380404	NA	NA	-3.903396863	0.004170442	NA	NA
LOC110370729	uncharacterized protein LOC110370729	-2.821056504	0.019843708	-4.195935428	0.00423004	NA	NA
LOC110375907	exonuclease GOR-like isoform X1	-1.384538646	3.38E-05	-1.083818328	0.004232491	NA	NA
LOC110375907	transducin beta-like protein 2 isoform X2	-1.384538646	3.38E-05	-1.083818328	0.004232491	NA	NA
LOC110371275	proton-coupled amino acid transporter-like protein CG1139	-2.501368808	0.04823475	-4.241092711	0.004232491	NA	NA
LOC110379215	E3 ubiquitin-protein ligase UBR3-like	NA	NA	1.120926655	0.004266875	NA	NA
LOC110377387	uncharacterized protein LOC110377387	NA	NA	-5.590637118	0.004294513	NA	NA
LOC110374521	uncharacterized protein LOC110374521	-4.190914334	0.034904833	-6.715483645	0.004304379	NA	NA
LOC110372098	mite allergen Der p 3-like	-3.548525925	0.002230591	-5.864691018	0.004359954	NA	NA
LOC110375650	uncharacterized protein LOC110375650	-2.521319167	0.036950889	-4.605024319	0.004399706	NA	NA
LOC110369619	uncharacterized protein LOC110369619	-3.145733939	0.005003601	-5.824965664	0.004399706	NA	NA
LOC110384309	alcohol dehydrogenase-like	NA	NA	1.448635726	0.004437548	NA	NA
LOC110378792	cytochrome b-c1 complex subunit 1, mitochondrial-like	-2.651937749	0.031718602	-5.950327043	0.004471069	NA	NA

LOC110374869	uncharacterized protein LOC110374869	NA	NA	-5.223093265	0.004475848	NA	NA
LOC110375883	uncharacterized protein LOC110375883	NA	NA	-6.391761252	0.004483828	NA	NA
LOC110381389	IQ and AAA domain-containing protein 1-like	NA	NA	-6.620124717	0.004517681	NA	NA
LOC110380527	sperm flagellar protein 2-like	-3.174362305	0.010909443	-6.003475387	0.004517681	NA	NA
LOC110383191	mucin-2-like	NA	NA	-4.667918893	0.004532522	NA	NA
LOC110382971	4-coumarate--CoA ligase 1-like	NA	NA	-1.248207056	0.004533066	NA	NA
LOC110383029	fork head domain transcription factor slp2-like	NA	NA	1.296598013	0.004533066	NA	NA
LOC110378164	nucleoside diphosphate kinase homolog 5-like isoform X1	-2.954768301	0.00817885	-3.595884495	0.004562632	NA	NA
LOC110378164	nucleoside diphosphate kinase homolog 5-like isoform X2	-2.954768301	0.00817885	-3.595884495	0.004562632	NA	NA
LOC110378051	stress response protein NST1-like	NA	NA	-4.641191722	0.004623057	NA	NA
LOC110384305	uncharacterized protein LOC110384305	NA	NA	-2.553564785	0.004683095	NA	NA
LOC110378598	1,5-anhydro-D-fructose reductase-like	NA	NA	-1.866214966	0.004703727	NA	NA
LOC110380310	protein lethal(2)essential for life-like	NA	NA	-1.808941034	0.004706368	NA	NA

LOC110374847	uncharacterized protein LOC110374847	-2.687941003	0.016519226	-5.781782318	0.004706368	NA	NA
LOC110381908	radial spoke head protein 9 homolog	NA	NA	-6.408064123	0.00471329	NA	NA
LOC110370708	ribonuclease H1-like	NA	NA	-1.984626327	0.004714072	NA	NA
LOC110384352	uncharacterized protein CG5902	NA	NA	-1.089539095	0.004714072	NA	NA
LOC110373815	NA	NA	NA	1.289462267	0.004781241	NA	NA
LOC110383462	NA	NA	NA	1.265677831	0.004803277	NA	NA
LOC110379805	uncharacterized protein LOC110379805	NA	NA	-6.632312066	0.004814616	NA	NA
LOC110369741	solute carrier family 2, facilitated glucose transporter member 3-like isoform X3	-2.113352032	0.001290752	-2.01633288	0.004823429	NA	NA
LOC110369741	solute carrier family 2, facilitated glucose transporter member 3-like isoform X5	-2.113352032	0.001290752	-2.01633288	0.004823429	NA	NA
LOC110369741	solute carrier family 2, facilitated glucose transporter member 3-like isoform X1	-2.113352032	0.001290752	-2.01633288	0.004823429	NA	NA
LOC110369741	solute carrier family 2, facilitated glucose transporter member 3-like isoform X2	-2.113352032	0.001290752	-2.01633288	0.004823429	NA	NA

LOC110369741	solute carrier family 2, facilitated glucose transporter member 3-like isoform X4	-2.113352032	0.001290752	-2.01633288	0.004823429	NA	NA
LOC110383253	pre-rRNA-processing protein TSR1 homolog	NA	NA	1.016565437	0.004862023	NA	NA
LOC110373995	androgen-dependent TFPI-regulating protein-like	NA	NA	1.221659552	0.004876604	NA	NA
LOC110378688	protein GUCD1	NA	NA	1.130019733	0.004897819	NA	NA
LOC110379192	carbonic anhydrase 1-like	NA	NA	-6.582713028	0.004914466	NA	NA
LOC110373497	ATP synthase subunit d, mitochondrial-like	NA	NA	-5.080834949	0.004914466	NA	NA
LOC110374348	WD repeat-containing protein 63	NA	NA	-4.446008835	0.004960439	NA	NA
LOC110378190	LOW QUALITY PROTEIN: uncharacterized protein LOC110378190	NA	NA	-1.902586574	0.004973663	NA	NA
LOC110378589	ankycorbin	NA	NA	-1.844401832	0.004981309	NA	NA
LOC110380811	protein D3-like	-2.772414155	0.022455134	-3.766064634	0.004985555	NA	NA
LOC110377302	cytochrome P450 4g15	NA	NA	-1.470057571	0.005042512	NA	NA
LOC110374868	dynein assembly factor 3, axonemal homolog	NA	NA	-2.408282422	0.005048972	NA	NA
LOC110383035	L-dopachrome tautomerase yellow-f2-like	NA	NA	-2.050403222	0.005055639	NA	NA
LOC110382484	ceramide glucosyltransferase	NA	NA	1.33259666	0.00509325	NA	NA
LOC110372854	dual oxidase isoform X2	NA	NA	1.709772432	0.00509325	NA	NA

LOC110372854	dual oxidase isoform X1	NA	NA	1.709772432	0.00509325	NA	NA
LOC110381430	sialin	NA	NA	1.191552677	0.005112297	NA	NA
LOC110371619	LOW QUALITY PROTEIN: uncharacterized protein LOC110371619	NA	NA	-5.834904169	0.005112521	NA	NA
LOC110373826	elongation factor-like GTPase 1	NA	NA	1.211329974	0.005191579	NA	NA
LOC110371272	NA	NA	NA	-4.597253898	0.005201114	NA	NA
LOC110380859	uncharacterized protein LOC110380859	-2.544778342	0.028913802	-5.752279451	0.005248436	NA	NA
LOC110384492	brachyurin-like	NA	NA	2.894892329	0.005293388	NA	NA
LOC110373305	uncharacterized protein LOC110373305	-3.135946619	0.013694433	-4.654055487	0.005390989	NA	NA
LOC110376997	mucin-2-like	NA	NA	-4.618647567	0.005403951	NA	NA
LOC110370837	uncharacterized protein LOC110370837	-3.588855537	0.009423019	-4.875742018	0.005420547	NA	NA
LOC110371736	cytochrome P450 6B5-like	NA	NA	-2.254854184	0.005449074	NA	NA
LOC110371080	uncharacterized protein LOC110371080 isoform X2	NA	NA	-3.524462364	0.005525838	NA	NA
LOC110371080	uncharacterized protein LOC110371080 isoform X1	NA	NA	-3.524462364	0.005525838	NA	NA
LOC110371244	NA	NA	NA	-1.355059682	0.005535225	NA	NA
LOC110383619	fork head domain transcription factor slp2-like	NA	NA	1.275146218	0.005552022	NA	NA
LOC110380523	NA	NA	NA	-5.720071376	0.005580514	NA	NA

LOC110380813	uncharacterized protein LOC110380813	NA	NA	-1.0651688	0.005580514	NA	NA
LOC110381811	anillin-like	NA	NA	1.287104664	0.005580514	NA	NA
LOC110370643	uncharacterized protein LOC110370643	NA	NA	1.167241243	0.005580984	NA	NA
LOC110371545	tctex1 domain-containing protein 1-like	NA	NA	-2.826808879	0.005592067	NA	NA
LOC110371635	uncharacterized transmembrane protein DDB_G0289901-like	-3.949655883	0.047221855	-6.463464671	0.005623265	NA	NA
LOC110375713	cytochrome P450 18a1-like	NA	NA	-1.437796784	0.005645981	NA	NA
LOC110373603	uncharacterized protein LOC110373603 isoform X1	-2.013242849	0.038142498	-2.819183602	0.005646633	NA	NA
LOC110373603	uncharacterized protein LOC110373603 isoform X2	-2.013242849	0.038142498	-2.819183602	0.005646633	NA	NA
LOC110375098	mitochondrial pyruvate carrier 1-like	NA	NA	1.067082011	0.00583519	NA	NA
LOC110373351	nose resistant to fluoxetine protein 6-like	NA	NA	2.385277303	0.005858959	NA	NA
LOC110382090	F-box/WD repeat-containing protein 11, partial	NA	NA	1.195934359	0.005905643	NA	NA
LOC110373184	mitochondrial 2-oxoglutarate/malate carrier protein-like	-2.660447238	0.030745862	-5.753486813	0.006017202	NA	NA

LOC110382181	uncharacterized protein LOC110382181	-2.928966247	0.015561129	-5.744808744	0.006023225	NA	NA
LOC110373870	uncharacterized protein LOC110373870	NA	NA	-4.514494875	0.006028428	NA	NA
LOC110378194	NA	-2.314688352	0.047016825	-5.626985296	0.006124823	NA	NA
LOC110372287	alpha-tocopherol transfer protein-like	NA	NA	-1.245261479	0.00614528	NA	NA
LOC110381124	NA	NA	NA	-6.264512814	0.006250832	NA	NA
LOC110383137	E3 ubiquitin-protein ligase RNF123-like, partial	-3.592987213	0.007821621	-4.693623639	0.006305856	NA	NA
LOC110378928	uncharacterized protein LOC110378928 isoform X1	NA	NA	-5.591105684	0.006349378	NA	NA
LOC110378928	uncharacterized protein LOC110378928 isoform X4	NA	NA	-5.591105684	0.006349378	NA	NA
LOC110378928	uncharacterized protein LOC110378928 isoform X2	NA	NA	-5.591105684	0.006349378	NA	NA
LOC110378928	uncharacterized protein LOC110378928 isoform X3	NA	NA	-5.591105684	0.006349378	NA	NA
LOC110375138	uncharacterized protein LOC110375138	-3.042441882	0.012279788	-5.718734579	0.006349378	NA	NA
LOC110378018	uncharacterized protein LOC110378018	NA	NA	-2.009149266	0.006387965	NA	NA

LOC110384046	uncharacterized protein MAL13P1.336-like isoform X2	NA	NA	-4.183153749	0.006465575	NA	NA
LOC110384046	uncharacterized protein LOC110384046 isoform X1	NA	NA	-4.183153749	0.006465575	NA	NA
LOC110379304	probable chitinase 2	NA	NA	-1.493373851	0.006535538	NA	NA
LOC110371959	radial spoke head 10 homolog B-like isoform X2	-3.3684838	0.010820013	-5.856224938	0.006543937	NA	NA
LOC110371959	radial spoke head 10 homolog B-like isoform X1	-3.3684838	0.010820013	-5.856224938	0.006543937	NA	NA
LOC110371959	radial spoke head 10 homolog B-like isoform X4	-3.3684838	0.010820013	-5.856224938	0.006543937	NA	NA
LOC110371959	radial spoke head 10 homolog B-like isoform X3	-3.3684838	0.010820013	-5.856224938	0.006543937	NA	NA
LOC110380525	NA	NA	NA	-6.543649111	0.006587372	NA	NA
LOC110374051	MICOS complex subunit Mic10-like	-3.553594723	0.00834448	-4.654108591	0.006647808	NA	NA
LOC110377771	putative cystathionine gamma-lyase 2	-2.581982761	0.012828969	-3.091951173	0.006682353	NA	NA
LOC110370667	uncharacterized protein LOC110370667	NA	NA	-4.548447402	0.006748379	NA	NA
LOC110375770	protein scabrous	NA	NA	1.588715913	0.006813604	NA	NA
LOC110371892	uncharacterized protein LOC110371892	NA	NA	1.040203397	0.006839612	NA	NA

LOC110372521	uncharacterized protein LOC110372521 isoform X1	NA	NA	-1.759466116	0.007032621	NA	NA
LOC110372521	uncharacterized protein LOC110372521 isoform X2	NA	NA	-1.759466116	0.007032621	NA	NA
LOC110371037	tRNA-dihydrouridine(47) synthase	NA	NA	1.08522829	0.007062869	NA	NA
LOC110375220	protein aurora borealis	-2.009398101	0.015272403	-2.329447388	0.007133503	NA	NA
LOC110369809	acid phosphatase type 7-like	NA	NA	-1.583802515	0.007149569	NA	NA
LOC110384625	chorion protein E1-like, partial	NA	NA	2.417338649	0.007164715	NA	NA
LOC110378209	dynein heavy chain 3, axonemal	NA	NA	-2.04283495	0.007174467	NA	NA
LOC110376904	uncharacterized protein LOC110376904 isoform X2	NA	NA	1.738763545	0.007252876	NA	NA
LOC110376904	uncharacterized protein LOC110376904 isoform X3	NA	NA	1.738763545	0.007252876	NA	NA
LOC110376904	uncharacterized protein LOC110376904 isoform X1	NA	NA	1.738763545	0.007252876	NA	NA
LOC110373366	O-acyltransferase like protein-like	NA	NA	2.344140728	0.007252876	NA	NA
LOC110379846	organic cation transporter protein-like, partial	NA	NA	-1.715519409	0.007270017	NA	NA

LOC110372985	MORN repeat-containing protein 5-like	-3.377495007	0.022977266	-4.909092049	0.007285084	NA	NA
LOC110371137	uncharacterized protein LOC110371137	NA	NA	1.326060863	0.007326619	NA	NA
LOC110372096	NA	NA	NA	1.391327322	0.007341206	NA	NA
LOC110374145	uncharacterized protein LOC110374145	NA	NA	1.192600992	0.007428052	NA	NA
LOC110374552	interferon-related developmental regulator 2	NA	NA	1.333193354	0.007428052	NA	NA
LOC110382934	uncharacterized protein LOC110382934	-2.819833811	0.001150412	-2.524013322	0.007461537	NA	NA
LOC110376493	uncharacterized protein LOC110376493 isoform X1	NA	NA	-5.558363062	0.007495007	NA	NA
LOC110376493	uncharacterized protein LOC110376493 isoform X2	NA	NA	-5.558363062	0.007495007	NA	NA
LOC110379701	uncharacterized protein LOC110379701	NA	NA	-1.817438475	0.007511345	NA	NA
LOC110378241	uncharacterized protein LOC110378241	-2.802642537	0.018211134	-3.634009155	0.007544369	NA	NA
LOC110379715	putative inorganic phosphate cotransporter	NA	NA	-1.679345366	0.007650359	NA	NA
LOC110369979	sex peptide receptor-like	NA	NA	1.16780931	0.007787278	NA	NA
LOC110373440	uncharacterized protein LOC110373440	NA	NA	1.450137733	0.007821131	NA	NA

LOC110376844	cytochrome c oxidase subunit 5B, mitochondrial-like	NA	NA	-4.104676917	0.00782339	NA	NA
LOC110380085	uncharacterized protein LOC110380085	NA	NA	2.050643644	0.007831715	NA	NA
LOC110379751	alpha-crystallin A chain-like	-2.647504924	0.035166294	-5.604742183	0.007906019	NA	NA
LOC110379624	NA	NA	NA	-1.372982979	0.007938532	NA	NA
LOC110380698	LOW QUALITY PROTEIN: hemolin-like	NA	NA	1.206593074	0.008066481	NA	NA
LOC110378833	WD repeat-containing protein 36	NA	NA	1.110909815	0.008192795	NA	NA
LOC110373608	mpv17-like protein	-3.314308102	0.019843708	-5.848437082	0.008218431	NA	NA
LOC110371843	testis-specific serine/threonine-protein kinase 1-like isoform X2	-1.779877345	0.045334865	-2.37863033	0.00822813	NA	NA
LOC110371843	testis-specific serine/threonine-protein kinase 1-like isoform X1	-1.779877345	0.045334865	-2.37863033	0.00822813	NA	NA
LOC110371843	testis-specific serine/threonine-protein kinase 1-like isoform X3	-1.779877345	0.045334865	-2.37863033	0.00822813	NA	NA
LOC110377364	uncharacterized protein LOC110377364	-3.241476445	0.000450928	-2.665038519	0.008262013	NA	NA
LOC110371300	cilia- and flagella-associated protein 20-like	NA	NA	-6.216107303	0.008428687	NA	NA
LOC110381845	protein fantom-like isoform X2	-3.959939177	0.007764134	-4.768804279	0.008430917	NA	NA

LOC110381845	protein fantom-like isoform X1	-3.959939177	0.007764134	-4.768804279	0.008430917	NA	NA
LOC110379307	myrosinase 1-like, partial	NA	NA	1.516799661	0.008481239	NA	NA
LOC110369617	actin-related protein 6	NA	NA	1.40719941	0.008495603	NA	NA
LOC110372537	leucine-rich repeat-containing protein 74A-like	-2.011789988	0.002185496	-1.884648991	0.008495603	NA	NA
LOC110381661	NA	-2.954681881	0.039030664	-5.80109016	0.008517713	NA	NA
LOC110383973	myrosinase 1-like	NA	NA	-5.559854954	0.008624819	NA	NA
LOC110372508	uncharacterized protein LOC110372508	-3.309519238	0.010822455	-5.613297335	0.008740461	NA	NA
LOC110379025	trypsin CFT-1-like, partial	1.897911553	0.000124898	1.479005663	0.008742102	NA	NA
LOC110379599	calmodulin-like isoform X1	-2.932989021	0.022719331	-5.600018991	0.008742102	NA	NA
LOC110379599	calmodulin-like isoform X2	-2.932989021	0.022719331	-5.600018991	0.008742102	NA	NA
LOC110379599	calmodulin-2-like isoform X3	-2.932989021	0.022719331	-5.600018991	0.008742102	NA	NA
LOC110375835	prostaglandin reductase 1-like isoform X1	NA	NA	1.123229625	0.008773234	NA	NA
LOC110375835	prostaglandin reductase 1-like isoform X2	NA	NA	1.123229625	0.008773234	NA	NA
LOC110382381	protein enabled-like	NA	NA	1.400579931	0.008883257	NA	NA
LOC110371721	uncharacterized protein LOC110371721	-3.11380356	0.016897034	-5.613054006	0.008883257	NA	NA
LOC110382490	uncharacterized protein LOC110382490 isoform X2	NA	NA	-3.953562632	0.00906227	NA	NA

LOC110382490	uncharacterized protein LOC110382490 isoform X1	NA	NA	-3.953562632	0.00906227	NA	NA
LOC110380241	nucleolar pre-ribosomal-associated protein 1	NA	NA	1.126653926	0.009157464	NA	NA
LOC110380950	acetylcholinesterase 1-like	NA	NA	-1.105519315	0.009173592	NA	NA
LOC110380780	spindle assembly checkpoint component MAD1-like	NA	NA	-5.99816962	0.009242978	NA	NA
LOC110372213	uncharacterized protein LOC110372213	NA	NA	-6.039614018	0.009291197	NA	NA
LOC110384374	radial spoke head 1 homolog	-3.1350655	0.005621962	-3.232715697	0.009383113	NA	NA
LOC110370372	arginase, hepatic isoform X2	NA	NA	-1.712589636	0.009408316	NA	NA
LOC110370372	arginase, hepatic isoform X1	NA	NA	-1.712589636	0.009408316	NA	NA
LOC110378355	NA	NA	NA	5.83356127	0.009457365	NA	NA
LOC110376752	uncharacterized protein LOC110376752	NA	NA	1.124527021	0.00948871	NA	NA
LOC110378216	uncharacterized protein LOC110378216	NA	NA	-3.433326988	0.009725489	NA	NA
LOC110373413	uncharacterized protein K02A2.6-like	NA	NA	2.246939017	0.009733375	NA	NA
LOC110381637	uncharacterized protein LOC110381637 isoform X2	NA	NA	-3.582740107	0.009794829	NA	NA

LOC110381637	uncharacterized protein LOC110381637 isoform X1	NA	NA	-3.582740107	0.009794829	NA	NA
LOC110381637	uncharacterized protein LOC110381637 isoform X3	NA	NA	-3.582740107	0.009794829	NA	NA
LOC110372183	uncharacterized protein LOC110372183	NA	NA	-4.336022395	0.009807036	NA	NA
LOC110382775	uncharacterized protein LOC110382775	-2.322082503	0.03459489	-3.023601057	0.009842156	NA	NA
LOC110379810	cilia- and flagella-associated protein 61-like	-3.100785008	0.036385571	-5.768443456	0.009991799	NA	NA
LOC110369703	uncharacterized protein LOC110369703 isoform X1	-2.454047096	0.038628215	-3.337380602	0.010040705	NA	NA
LOC110369703	uncharacterized protein LOC110369703 isoform X2	-2.454047096	0.038628215	-3.337380602	0.010040705	NA	NA
LOC110370815	cytosolic 10-formyltetrahydrofolate dehydrogenase	NA	NA	-1.799004743	0.010082076	NA	NA
LOC110373040	otoferlin-like	NA	NA	-6.256145768	0.010112484	NA	NA
LOC110377863	tubulin alpha-1 chain-like	NA	NA	-5.594293472	0.010154298	NA	NA
LOC110378714	nitric oxide synthase-like protein isoform X2	NA	NA	1.687879766	0.010166468	NA	NA
LOC110378714	nitric oxide synthase-like protein isoform X1	NA	NA	1.687879766	0.010166468	NA	NA

LOC110384319	uncharacterized protein LOC110384319 isoform X3	-1.103331056	0.028630108	-1.29669639	0.010275703	NA	NA
LOC110384319	uncharacterized protein LOC110384319 isoform X4	-1.103331056	0.028630108	-1.29669639	0.010275703	NA	NA
LOC110384319	uncharacterized protein LOC110384319 isoform X2	-1.103331056	0.028630108	-1.29669639	0.010275703	NA	NA
LOC110384319	uncharacterized protein LOC110384319 isoform X1	-1.103331056	0.028630108	-1.29669639	0.010275703	NA	NA
LOC110370516	UPF0728 protein-like	NA	NA	-3.782170557	0.010344671	NA	NA
LOC110374102	sperm mitochondrial-associated cysteine-rich protein-like	NA	NA	-1.201807588	0.010374784	NA	NA
LOC110381456	LOW QUALITY PROTEIN: uncharacterized protein LOC110381456	NA	NA	-2.00259713	0.010404086	NA	NA
LOC110378868	uncharacterized protein LOC110378868	NA	NA	-5.800169707	0.010492514	NA	NA
LOC110382501	uncharacterized protein LOC110382501, partial	NA	NA	1.29725817	0.010503922	NA	NA
LOC110377628	uncharacterized protein LOC110377628 isoform X1	-3.252824346	0.016479542	-5.561738925	0.010503922	NA	NA
LOC110377628	uncharacterized protein LOC110377628 isoform X2	-3.252824346	0.016479542	-5.561738925	0.010503922	NA	NA

LOC110370052	mismatch repair endonuclease PMS2	NA	NA	-1.320059973	0.010724296	NA	NA
LOC110374703	pyruvate kinase-like	NA	NA	-5.679351577	0.010784693	NA	NA
LOC110379100	thrombospondin type-1 domain-containing protein 7A-like	NA	NA	2.037093033	0.010905094	NA	NA
LOC110370906	uncharacterized protein LOC110370906	NA	NA	-3.918559099	0.01091424	NA	NA
LOC110370508	zinc finger and BTB domain-containing protein 17 isoform X1	NA	NA	-2.973165673	0.01093653	NA	NA
LOC110370508	zinc finger and BTB domain-containing protein 17 isoform X2	NA	NA	-2.973165673	0.01093653	NA	NA
LOC110379638	uncharacterized protein LOC110379638	NA	NA	-5.454304374	0.011076477	NA	NA
LOC110379187	lisH domain-containing protein FOPNL-like	-3.142399025	0.025563382	-4.469226645	0.011185593	NA	NA
LOC110374299	uncharacterized protein LOC110374299 isoform X2	NA	NA	-6.631001121	0.011251742	NA	NA
LOC110374299	uncharacterized protein LOC110374299 isoform X1	NA	NA	-6.631001121	0.011251742	NA	NA
LOC110374327	EF-hand domain-containing protein 1-like	-1.671039459	0.022267194	-1.896006666	0.011354996	NA	NA
LOC110372903	uncharacterized transmembrane protein DDB_G0289901-like	-2.25644149	0.022267194	-2.676282165	0.011463008	NA	NA

LOC110372133	probable aldehyde oxidase 2	2.031807134	7.65E-05	1.499875923	0.011603905	NA	NA
LOC110379264	uncharacterized protein LOC110379264	-2.715239255	0.035057499	-3.800827726	0.011663155	NA	NA
LOC110371729	coiled-coil domain-containing protein 42 homolog	-2.500680601	0.036079397	-3.222244364	0.011670587	NA	NA
LOC110374507	NA	-3.230988889	0.019897426	-5.528515503	0.011763549	NA	NA
LOC110377214	uncharacterized protein LOC110377214	NA	NA	-1.857376674	0.011786292	NA	NA
LOC110378502	uncharacterized protein LOC110378502	NA	NA	-4.286735203	0.01190626	NA	NA
LOC110376435	myosin-I heavy chain	-1.451431307	0.000451151	-1.174034958	0.012068147	NA	NA
LOC110379702	putative inorganic phosphate cotransporter	NA	NA	-1.646189679	0.012213647	NA	NA
LOC110369628	aldo-keto reductase AKR2E4-like	-1.668644843	0.034869387	-1.959069561	0.012213647	NA	NA
LOC110374026	mast cell protease 1-like	NA	NA	-2.456622339	0.012221856	NA	NA
LOC110378467	organic cation transporter protein-like	NA	NA	-1.411191517	0.012329564	NA	NA
LOC110372334	uncharacterized protein LOC110372334	NA	NA	-1.038770398	0.012726239	NA	NA
LOC110384166	luciferin 4-monooxygenase	NA	NA	1.057705427	0.013033651	NA	NA
LOC110375384	NA	NA	NA	-1.045680655	0.013086265	NA	NA
LOC110369686	MORN repeat-containing protein 3-like	NA	NA	-6.148250989	0.013113167	NA	NA
LOC110381698	carbonic anhydrase 7	-1.078372491	0.04823475	-1.322371816	0.013154725	NA	NA

LOC110376684	multidrug resistance protein homolog 49-like	-2.263980545	0.005296552	-2.177855073	0.013158474	NA	NA
LOC110372349	SUN domain-containing protein 2-like	NA	NA	-4.262729131	0.013271794	NA	NA
LOC110375879	NADH dehydrogenase	NA	NA	-3.332987271	0.013415701	NA	NA
LOC110377402	heparan-alpha-glucosaminide N-acetyltransferase, partial	-1.098326906	0.018987303	-1.198664039	0.01348083	NA	NA
LOC110371648	NA	NA	NA	-1.57370127	0.013557242	NA	NA
LOC110377829	uncharacterized protein LOC110377829 isoform X4	NA	NA	1.136032694	0.013830367	NA	NA
LOC110377829	uncharacterized protein LOC110377829 isoform X2	NA	NA	1.136032694	0.013830367	NA	NA
LOC110377829	uncharacterized protein LOC110377829 isoform X1	NA	NA	1.136032694	0.013830367	NA	NA
LOC110377829	uncharacterized protein LOC110377829 isoform X3	NA	NA	1.136032694	0.013830367	NA	NA
LOC110374000	uncharacterized protein LOC110374000	-2.90143102	0.045357633	-4.391239783	0.013832442	NA	NA
LOC110374756	TP53-regulated inhibitor of apoptosis 1-like	NA	NA	1.143896368	0.013907332	NA	NA
LOC110383126	uncharacterized protein LOC110383126	NA	NA	1.146028557	0.014045309	NA	NA
LOC110383208	proteoglycan Cow	NA	NA	-1.913560293	0.01409259	NA	NA

LOC110382792	uncharacterized protein LOC110382792	NA	NA	-3.663615955	0.014153183	NA	NA
LOC110379323	protein lethal(2)essential for life-like	NA	NA	-2.095862053	0.014330497	NA	NA
LOC110380496	NA	NA	NA	-3.717477501	0.014490943	NA	NA
LOC110372882	uncharacterized protein LOC110372882 isoform X1	NA	NA	-4.219603483	0.014652473	NA	NA
LOC110372882	MORN repeat-containing protein 5-like isoform X2	NA	NA	-4.219603483	0.014652473	NA	NA
LOC110383825	NA	NA	NA	1.149550613	0.014770414	NA	NA
LOC110383773	facilitated trehalose transporter Tret1-like	NA	NA	-1.010277362	0.01506215	NA	NA
LOC110372228	NA	NA	NA	-1.420259007	0.015139955	NA	NA
LOC110375671	72 kDa inositol polyphosphate 5-phosphatase isoform X1	NA	NA	1.116465371	0.015348126	NA	NA
LOC110375671	72 kDa inositol polyphosphate 5-phosphatase isoform X4	NA	NA	1.116465371	0.015348126	NA	NA
LOC110375671	72 kDa inositol polyphosphate 5-phosphatase isoform X2	NA	NA	1.116465371	0.015348126	NA	NA
LOC110375671	72 kDa inositol polyphosphate 5-phosphatase isoform X3	NA	NA	1.116465371	0.015348126	NA	NA
LOC110374273	uncharacterized protein LOC110374273	NA	NA	-6.122577289	0.015732653	NA	NA
LOC110379193	carbonic anhydrase 6-like	NA	NA	-4.537732046	0.015850259	NA	NA

LOC110379462	NA	NA	NA	-1.727846897	0.015850259	NA	NA
LOC110372916	oxysterol-binding protein-related protein 1 isoform X1	NA	NA	2.740987279	0.015916201	NA	NA
LOC110372916	oxysterol-binding protein-related protein 1 isoform X3	NA	NA	2.740987279	0.015916201	NA	NA
LOC110372916	oxysterol-binding protein-related protein 1 isoform X2	NA	NA	2.740987279	0.015916201	NA	NA
LOC110381951	uncharacterized protein LOC110381951	NA	NA	-1.192594777	0.015939313	NA	NA
LOC110381371	uncharacterized protein LOC110381371	NA	NA	-1.425963943	0.015984219	NA	NA
LOC110374854	histidine-rich glycoprotein-like isoform X1	-2.685771442	0.048756094	-3.76277797	0.01606509	NA	NA
LOC110374854	histidine-rich glycoprotein-like isoform X2	-2.685771442	0.048756094	-3.76277797	0.01606509	NA	NA
LOC110381173	uncharacterized protein LOC110381173	NA	NA	-2.747247966	0.016530444	NA	NA
LOC110383042	NA	NA	NA	-1.718367283	0.016903339	NA	NA
LOC110374200	carbohydrate sulfotransferase 4-like	NA	NA	-2.749754215	0.017251106	NA	NA
LOC110376665	beta-1,3-galactosyltransferase 1-like isoform X1	NA	NA	-5.303655284	0.017263475	NA	NA

LOC110376665	beta-1,3-galactosyltransferase 1-like isoform X2	NA	NA	-5.303655284	0.017263475	NA	NA
LOC110371845	testis-specific serine/threonine-protein kinase 1-like	NA	NA	-2.483243838	0.017441204	NA	NA
LOC110380449	carboxypeptidase inhibitor	NA	NA	-6.024404889	0.017628185	NA	NA
LOC110376359	uncharacterized protein LOC110376359	NA	NA	-3.780170602	0.017644985	NA	NA
LOC110372576	zinc finger CW-type PWWP domain protein 1-like isoform X2	NA	NA	-6.190420195	0.018476684	NA	NA
LOC110372576	zinc finger CW-type PWWP domain protein 1-like isoform X1	NA	NA	-6.190420195	0.018476684	NA	NA
LOC110370112	LOW QUALITY PROTEIN: sodium-coupled monocarboxylate transporter 2-like	NA	NA	-1.125163041	0.018522364	NA	NA
LOC110381855	uncharacterized protein LOC110381855, partial	-3.41767277	0.032319578	-4.488549741	0.018522364	NA	NA
LOC110378114	enhancer of split mbeta protein-like	NA	NA	-1.033468065	0.01860337	NA	NA
LOC110370247	uncharacterized protein LOC110370247	NA	NA	-6.20690012	0.018742607	NA	NA
LOC110370602	uncharacterized protein LOC110370602	NA	NA	-3.996566359	0.018742607	NA	NA

LOC110374304	uncharacterized protein LOC110374304	NA	NA	-4.319880636	0.018902591	NA	NA
LOC110372033	NA	NA	NA	2.164710638	0.018902591	NA	NA
LOC110376937	uncharacterized protein LOC110376937	NA	NA	-2.57268209	0.019045722	NA	NA
LOC110373299	glutathione S-transferase-like	-3.471725128	0.005990765	-3.22235476	0.019057328	NA	NA
LOC110372564	leucine-rich repeat neuronal protein 2	NA	NA	1.015061817	0.019117319	NA	NA
LOC110379651	uncharacterized protein LOC110379651	-2.925634321	0.043113757	-5.23299075	0.019117319	NA	NA
LOC110380447	uncharacterized protein LOC110380447	NA	NA	-3.555607935	0.019391585	NA	NA
LOC110383665	trypsin, alkaline C-like isoform X1	NA	NA	-6.032348105	0.019409171	NA	NA
LOC110383665	trypsin, alkaline C-like isoform X2	NA	NA	-6.032348105	0.019409171	NA	NA
LOC110376144	synaptic vesicle glycoprotein 2B-like	-1.158752978	0.018158379	-1.193946306	0.019683366	NA	NA
LOC110379137	cGMP-dependent 3\\',5\\'-cyclic phosphodiesterase-like	NA	NA	-6.069587952	0.019964482	NA	NA
LOC110372944	uncharacterized protein LOC110372944	-3.498423065	0.012302693	-3.571798333	0.020187176	NA	NA
LOC110380506	NA	NA	NA	-1.887797379	0.020191326	NA	NA
LOC110381556	glutamate-rich protein 2	NA	NA	-5.965068349	0.020449963	NA	NA
LOC110378767	maltase 2-like	NA	NA	1.289709314	0.020488962	NA	NA

LOC110377224	adenylate cyclase type 10-like	NA	NA	-5.267935547	0.020781468	NA	NA
LOC110381991	4-coumarate--CoA ligase 1-like	-1.516990632	0.014820366	-1.534455943	0.020781468	NA	NA
LOC110381891	zonadhesin-like, partial	-1.476702369	0.01111555	-1.436036117	0.021048315	NA	NA
LOC110374639	pyruvate kinase-like	-1.905196571	0.000655338	-1.463048923	0.021388	NA	NA
LOC110375860	uncharacterized protein LOC110375860	NA	NA	1.949582318	0.022018245	NA	NA
LOC110377162	NA	NA	NA	-5.975525383	0.022328996	NA	NA
LOC110369882	MD-2-related lipid-recognition protein-like	NA	NA	-1.357793942	0.022426319	NA	NA
LOC110371013	defense protein 6-like	NA	NA	3.451009194	0.022785966	NA	NA
LOC110375002	maltase A1-like	NA	NA	-2.411458937	0.022923466	NA	NA
LOC110379986	glutamine synthetase-like	NA	NA	-6.040959949	0.023498688	NA	NA
LOC110377126	NA	-3.757191879	0.038329544	-5.588960451	0.023498688	NA	NA
LOC110371075	homeobox protein aristaless-like	NA	NA	1.268181499	0.023593373	NA	NA
LOC110371901	protein PIH1D3	NA	NA	-3.03164902	0.023642361	NA	NA
LOC110372404	HMG box-containing protein C19G7.04-like	NA	NA	-1.748279286	0.023642361	NA	NA
LOC110373297	uncharacterized protein LOC110373297 isoform X1	NA	NA	1.890031472	0.023712053	NA	NA
LOC110373297	uncharacterized protein LOC110373297 isoform X2	NA	NA	1.890031472	0.023712053	NA	NA
LOC110381398	uncharacterized protein LOC110381398	NA	NA	-5.724731022	0.024166826	NA	NA

LOC110384647	putative ATP-dependent RNA helicase TDRD12	NA	NA	1.197334256	0.024166826	NA	NA
LOC110370599	sarcoplasmic reticulum histidine-rich calcium-binding protein-like	NA	NA	1.357656862	0.024211113	NA	NA
LOC110379263	uncharacterized protein LOC110379263	-2.179436735	0.005016033	-1.932243994	0.024211113	NA	NA
LOC110379946	facilitated trehalose transporter Tret1-like	NA	NA	-2.81807654	0.024262557	NA	NA
LOC110370116	NA	NA	NA	-1.924206425	0.024370579	NA	NA
LOC110374037	growth arrest-specific protein 8	NA	NA	-3.514082785	0.024380626	NA	NA
LOC110380407	probable serine/threonine-protein kinase clkA	NA	NA	-5.553741877	0.024552338	NA	NA
LOC110378770	uncharacterized protein LOC110378770	NA	NA	-3.059289509	0.024583586	NA	NA
LOC110379161	sorting nexin-8-like	NA	NA	1.223868199	0.02461007	NA	NA
LOC110378793	gloverin-like	NA	NA	2.659421673	0.024626755	NA	NA
LOC110378803	gloverin-like	1.372480427	0.034959051	1.472095392	0.024908123	NA	NA
LOC110371455	uncharacterized protein LOC110371455	NA	NA	1.31582804	0.025041253	NA	NA
LOC110374898	DNA oxidative demethylase ALKBH2-like	NA	NA	1.762855759	0.025190481	NA	NA
LOC110371363	uncharacterized protein LOC110371363 isoform X2	NA	NA	-2.410439794	0.025499377	NA	NA

LOC110371363	uncharacterized protein LOC110371363 isoform X1	NA	NA	-2.410439794	0.025499377	NA	NA
LOC110374727	uncharacterized protein LOC110374727	NA	NA	-3.85825276	0.026034458	NA	NA
LOC110383908	NA	NA	NA	-1.632611994	0.026426487	NA	NA
LOC110378203	uncharacterized protein LOC110378203	NA	NA	1.080892603	0.026877853	NA	NA
LOC110380961	major heat shock 70 kDa protein Ab-like	NA	NA	-1.171698341	0.027015436	NA	NA
LOC110370453	elongation of very long chain fatty acids protein AAEL008004-like	NA	NA	-2.960150022	0.027115205	NA	NA
LOC110375987	frizzled-2-like	NA	NA	-1.026902761	0.027518804	NA	NA
LOC110378310	uncharacterized protein LOC110378310	NA	NA	2.428026939	0.028467891	NA	NA
LOC110383189	uncharacterized protein LOC110383189	NA	NA	1.544273059	0.028728971	NA	NA
LOC110373880	uncharacterized protein LOC110373880	-3.704914507	0.028194639	-5.19640223	0.02878742	NA	NA
LOC110382370	uncharacterized protein LOC110382370	NA	NA	1.029136465	0.029009427	NA	NA
LOC110383332	putative fatty acyl-CoA reductase CG5065 isoform X6	NA	NA	-2.938604515	0.029124272	NA	NA
LOC110383332	putative fatty acyl-CoA reductase CG5065 isoform X4	NA	NA	-2.938604515	0.029124272	NA	NA

LOC110383332	putative fatty acyl-CoA reductase CG5065 isoform X2	NA	NA	-2.938604515	0.029124272	NA	NA
LOC110383332	putative fatty acyl-CoA reductase CG5065 isoform X5	NA	NA	-2.938604515	0.029124272	NA	NA
LOC110383332	putative fatty acyl-CoA reductase CG5065 isoform X1	NA	NA	-2.938604515	0.029124272	NA	NA
LOC110383332	putative fatty acyl-CoA reductase CG5065 isoform X3	NA	NA	-2.938604515	0.029124272	NA	NA
LOC110374503	uncharacterized protein LOC110374503	NA	NA	-6.11302164	0.02966021	NA	NA
LOC110380874	GILT-like protein 1	NA	NA	-1.05575826	0.029694612	NA	NA
LOC110370501	probable rRNA-processing protein EBP2 homolog	NA	NA	1.009358452	0.02971518	NA	NA
LOC110372708	NA	NA	NA	-1.28492186	0.029765847	NA	NA
LOC110371019	uncharacterized protein LOC110371019	NA	NA	4.353524851	0.029938905	NA	NA
LOC110378962	glucose dehydrogenase	NA	NA	-1.026034154	0.030034584	NA	NA
LOC110375056	uncharacterized protein LOC110375056	-3.255623726	0.045018278	-5.089147815	0.030034584	NA	NA
LOC110378515	uncharacterized protein LOC110378515	NA	NA	-5.068424238	0.030314698	NA	NA
LOC110378735	LOW QUALITY PROTEIN: uncharacterized protein LOC110378735	NA	NA	1.026290748	0.030367466	NA	NA

LOC110377947	histone H2A, sperm-like	NA	NA	-3.427872078	0.03103005	NA	NA
LOC110371730	peptidyl-tRNA hydrolase 2, mitochondrial-like	NA	NA	1.000224842	0.031060076	NA	NA
LOC110381044	cytochrome b-c1 complex subunit 2, mitochondrial-like	NA	NA	-1.589472824	0.03172337	NA	NA
LOC110375149	cilia- and flagella-associated protein 161-like	NA	NA	-5.055786013	0.0319218	NA	NA
LOC110377580	uncharacterized protein LOC110377580	NA	NA	1.152450409	0.032271967	NA	NA
LOC110370826	glucose dehydrogenase	NA	NA	1.167011402	0.032334226	NA	NA
LOC110371343	glutathione S-transferase 1-like	NA	NA	1.164688891	0.032809674	NA	NA
LOC110374940	CCAAT/enhancer-binding protein-like	-2.855996957	2.74E-08	-1.362416086	0.033539902	NA	NA
LOC110381225	neuroglian-like	NA	NA	-1.14395973	0.03383724	NA	NA
LOC110384441	uncharacterized protein LOC110384441	NA	NA	-3.809649545	0.033868263	NA	NA
LOC110381619	UDP-glucuronosyltransferase 2B17-like	NA	NA	1.288159117	0.034211199	NA	NA
LOC110375911	LOW QUALITY PROTEIN: uncharacterized protein LOC110375911	NA	NA	1.12344049	0.034286475	NA	NA
LOC110371628	synaptic vesicle glycoprotein 2C-like	NA	NA	-2.272213445	0.034624506	NA	NA

LOC110375762	gastrula zinc finger protein XlCGF26.1-like isoform X2	NA	NA	1.298161297	0.034802612	NA	NA
LOC110375762	gastrula zinc finger protein XlCGF26.1-like isoform X1	NA	NA	1.298161297	0.034802612	NA	NA
LOC110380819	caltractin-like isoform X2	NA	NA	-3.966343311	0.034845228	NA	NA
LOC110380819	caltractin-like isoform X1	NA	NA	-3.966343311	0.034845228	NA	NA
LOC110370562	uncharacterized protein LOC110370562	NA	NA	-1.006097162	0.035429011	NA	NA
LOC110370007	macrophage mannose receptor 1-like	NA	NA	1.509109656	0.035429011	NA	NA
LOC110376604	facilitated trehalose transporter Tret1-like	NA	NA	1.0037647	0.035494919	NA	NA
LOC110381455	alpha-1,6-mannosyl-glycoprotein 2-beta-N-acetylglucosaminyltransferase-like	NA	NA	-5.885564566	0.035815217	NA	NA
LOC110382348	microtubule-associated protein futsch-like, partial	NA	NA	1.735049529	0.035936961	NA	NA
LOC110370696	three prime repair exonuclease 2-like	NA	NA	1.052620553	0.03620106	NA	NA
LOC110383345	uncharacterized protein LOC110383345 isoform X2	NA	NA	3.350038739	0.036253152	NA	NA
LOC110383345	uncharacterized protein LOC110383345 isoform X1	NA	NA	3.350038739	0.036253152	NA	NA

LOC110383345	uncharacterized protein LOC110383345 isoform X3	NA	NA	3.350038739	0.036253152	NA	NA
LOC110383345	uncharacterized protein LOC110383345 isoform X4	NA	NA	3.350038739	0.036253152	NA	NA
LOC110374331	uncharacterized protein LOC110374331	NA	NA	-1.933591398	0.036369315	NA	NA
LOC110375137	uncharacterized protein LOC110375137	NA	NA	-5.576807879	0.036473539	NA	NA
LOC110372742	apolipoprotein D-like	NA	NA	-1.707038481	0.036642983	NA	NA
LOC110375653	serine-rich adhesin for platelets-like	NA	NA	-2.721738062	0.036784638	NA	NA
LOC110378273	synaptic vesicle glycoprotein 2C-like, partial	NA	NA	-1.010469936	0.03706247	NA	NA
LOC110384028	short transient receptor potential channel 4-like	NA	NA	-3.116295699	0.037248131	NA	NA
LOC110381738	anionic antimicrobial peptide 2-like	NA	NA	-1.881303658	0.037858389	NA	NA
LOC110378875	uncharacterized protein LOC110378875	NA	NA	-1.295114634	0.037903977	NA	NA
LOC110380134	uncharacterized protein LOC110380134	NA	NA	-5.564542715	0.037965117	NA	NA
LOC110378212	uncharacterized protein LOC110378212	NA	NA	-4.861815649	0.037965117	NA	NA
LOC110379260	titin-like isoform X3	-2.902713076	0.000169463	-1.917832243	0.037965117	NA	NA

LOC110379260	uncharacterized protein LOC110379260 isoform X2	-2.902713076	0.000169463	-1.917832243	0.037965117	NA	NA
LOC110379260	uncharacterized protein LOC110379260 isoform X1	-2.902713076	0.000169463	-1.917832243	0.037965117	NA	NA
LOC110378861	cytochrome P450 4C1-like	-2.179962343	0.038201025	-2.249319469	0.038063347	NA	NA
LOC110374009	uncharacterized protein LOC110374009	NA	NA	-5.115022884	0.038359583	NA	NA
LOC110376358	uncharacterized protein LOC110376358	NA	NA	1.818543522	0.038404026	NA	NA
LOC110370273	C-Myc-binding protein	NA	NA	-1.158474496	0.038779354	NA	NA
LOC110379038	inhibin beta B chain	-1.371397646	0.018096069	-1.293950553	0.038899407	NA	NA
LOC110376551	potassium/sodium hyperpolarization-activated cyclic nucleotide-gated channel 1-like	NA	NA	-5.107808583	0.039107457	NA	NA
LOC110379919	coiled-coil domain-containing protein 113-like	-3.708628024	0.035556347	-3.970678553	0.039344441	NA	NA
LOC110380346	monocarboxylate transporter 9-like	NA	NA	-3.718154197	0.039541008	NA	NA
LOC110379350	uncharacterized protein LOC110379350	NA	NA	2.529830019	0.039602098	NA	NA
LOC110377012	golgin subfamily A member 3-like	NA	NA	-5.199168502	0.039728434	NA	NA

LOC110371410	Down syndrome cell adhesion molecule-like protein Dscam2	NA	NA	-3.017663167	0.039819541	NA	NA
LOC110377768	uncharacterized protein LOC110377768 isoform X1	NA	NA	-1.680466124	0.03997493	NA	NA
LOC110377768	uncharacterized protein LOC110377768 isoform X2	NA	NA	-1.680466124	0.03997493	NA	NA
LOC110377768	uncharacterized protein LOC110377768 isoform X3	NA	NA	-1.680466124	0.03997493	NA	NA
LOC110377768	uncharacterized protein LOC110377768 isoform X4	NA	NA	-1.680466124	0.03997493	NA	NA
LOC110378109	suppressor of cytokine signaling 2-like isoform X1	NA	NA	1.031661047	0.03999492	NA	NA
LOC110378109	suppressor of cytokine signaling 2-like isoform X2	NA	NA	1.031661047	0.03999492	NA	NA
LOC110382966	NA	-1.983978169	0.031948394	-2.011490281	0.040007186	NA	NA
LOC110384494	NA	1.128583687	0.021739365	1.079179879	0.040294334	NA	NA
LOC110370784	centromere protein J	NA	NA	-1.352424376	0.040596664	NA	NA
LOC110374094	G2/mitotic-specific cyclin-B isoform X1	NA	NA	-1.652189964	0.041272668	NA	NA
LOC110374094	G2/mitotic-specific cyclin-B isoform X3	NA	NA	-1.652189964	0.041272668	NA	NA

LOC110374094	G2/mitotic-specific cyclin-B isoform X2	NA	NA	-1.652189964	0.041272668	NA	NA
LOC110376643	uncharacterized protein LOC110376643	-1.838631032	0.046625063	-1.949527584	0.041579439	NA	NA
LOC110382711	uncharacterized protein LOC110382711	NA	NA	-2.122763659	0.041932322	NA	NA
LOC110376738	rhomboid-related protein 2 isoform X1	NA	NA	2.796651438	0.042228694	NA	NA
LOC110376738	rhomboid-related protein 3 isoform X2	NA	NA	2.796651438	0.042228694	NA	NA
LOC110383305	axonemal 84 kDa protein-like	-1.552801492	0.017594595	-1.444527007	0.042470966	NA	NA
LOC110379213	uncharacterized protein LOC110379213	NA	NA	-1.54822093	0.04252758	NA	NA
LOC110379584	cilia- and flagella-associated protein 36	NA	NA	-1.923772747	0.043245686	NA	NA
LOC110372863	uncharacterized protein LOC110372863	NA	NA	-3.641842477	0.043249464	NA	NA
LOC110373563	dynein light chain roadblock-type 2	NA	NA	-4.070233837	0.043698387	NA	NA
LOC110370334	uncharacterized protein LOC110370334	NA	NA	-1.00798062	0.044158172	NA	NA
LOC110378318	uncharacterized protein LOC110378318	NA	NA	-1.150788808	0.044190116	NA	NA
LOC110369818	MD-2-related lipid-recognition protein-like	NA	NA	-1.766209986	0.044290128	NA	NA
LOC110372504	NA	NA	NA	-3.465164477	0.044366314	NA	NA
LOC110381926	NA	-3.814785552	0.026476218	-3.750221137	0.044861811	NA	NA

LOC110380498	NA	NA	NA	-2.29355849	0.045259719	NA	NA
LOC110382395	clusterin-associated protein 1	NA	NA	-5.061704773	0.045528639	NA	NA
LOC110377670	peroxidase-like	NA	NA	-3.475733272	0.04555461	NA	NA
LOC110378202	alpha-tocopherol transfer protein-like	NA	NA	2.01535013	0.045813287	NA	NA
LOC110381683	zonadhesin-like, partial	-1.677823387	0.006381038	-1.392844865	0.046113809	NA	NA
LOC110383424	LOW QUALITY PROTEIN: S-adenosylmethionine decarboxylase proenzyme, partial	NA	NA	1.140387074	0.046132102	NA	NA
LOC110380998	protein abrupt isoform X1	NA	NA	1.220525647	0.046166904	NA	NA
LOC110380998	protein abrupt isoform X2	NA	NA	1.220525647	0.046166904	NA	NA
LOC110380998	protein abrupt isoform X3	NA	NA	1.220525647	0.046166904	NA	NA
LOC110373766	uncharacterized protein LOC110373766	NA	NA	-3.840098768	0.046286153	NA	NA
LOC110381115	serine/threonine-protein kinase MAK	NA	NA	-3.437038286	0.046675722	NA	NA
LOC110370646	NA	NA	NA	1.378008464	0.047278581	NA	NA
LOC110377467	chromosome-associated kinesin KIF4-like	NA	NA	-3.298365587	0.047570672	NA	NA
LOC110378187	proton-coupled amino acid transporter-like protein CG1139	NA	NA	-1.18656822	0.047633385	NA	NA
LOC110376494	NA	NA	NA	-1.058363599	0.047906813	NA	NA

LOC110378941	glutaminase liver isoform, mitochondrial-like isoform X1	NA	NA	-2.038671377	0.047951231	NA	NA
LOC110378941	glutaminase liver isoform, mitochondrial-like isoform X3	NA	NA	-2.038671377	0.047951231	NA	NA
LOC110378941	glutaminase liver isoform, mitochondrial-like isoform X2	NA	NA	-2.038671377	0.047951231	NA	NA
LOC110370864	protein ABHD11-like isoform X1	NA	NA	-5.710173168	0.047953351	NA	NA
LOC110370864	protein ABHD11-like isoform X2	NA	NA	-5.710173168	0.047953351	NA	NA
LOC110373654	crossover junction endonuclease EME1	NA	NA	-1.192269679	0.049215196	NA	NA
LOC110384574	uncharacterized protein LOC110384574	NA	NA	-7.693230467	0.049327778	NA	NA
LOC110376009	netrin receptor UNC5C-like	-1.317533922	0.006739175	-1.080383433	0.049602738	NA	NA
LOC110371322	esterase FE4-like	NA	NA	NA	NA	-21.45508743	8.27E-05
LOC110371485	uncharacterized protein LOC110378868	NA	NA	NA	NA	-7.193662447	0.001041371
LOC110377986	craniofacial development protein 2-like	NA	NA	NA	NA	-7.035152409	5.68E-61
LOC110371804	tektin-4-like	NA	NA	NA	NA	-6.532934654	0.000950836
LOC110371804	probable tubulin polyglutamylase TTLL1	NA	NA	NA	NA	-6.404272006	0.001208758
LOC110371804	trypsin CFT-1-like	NA	NA	NA	NA	-6.389811732	0.004014573

LOC110376297	LOW QUALITY PROTEIN: uncharacterized protein LOC110371619	NA	NA	NA	NA	-6.330713667	0.002892288
LOC110377366	uncharacterized protein LOC110380814 isoform X1	NA	NA	NA	NA	-6.310465719	0.001552271
LOC110377366	uncharacterized protein LOC110380814 isoform X2	NA	NA	NA	NA	-6.310465719	0.001552271
LOC110380264	NA	NA	NA	NA	NA	-6.151135654	0.002683447
LOC110380264	uncharacterized protein LOC110373622	NA	NA	NA	NA	-6.12558641	4.78E-12
LOC110380264	myrosinase 1-like	NA	NA	NA	NA	-6.083172518	0.004817686
LOC110380264	pre-mRNA 3\\' end processing protein WDR33-like	NA	NA	NA	NA	-6.032220281	0.002745697
LOC110380264	uncharacterized protein LOC110378029	NA	NA	NA	NA	-6.030030014	0.003635693
LOC110380264	sodium/potassium-transporting ATPase subunit alpha-like	NA	NA	NA	NA	-5.911792	0.004781416
LOC110380264	uncharacterized protein LOC110374758	NA	NA	NA	NA	-5.863305411	0.002662918
LOC110380264	uncharacterized protein LOC110381762, partial	NA	NA	NA	NA	-5.729295372	3.13E-05
LOC110380305	O-acyltransferase like protein-like	NA	NA	NA	NA	-5.307511642	3.19E-08
LOC110380305	tachykinin-like peptides receptor 86C	NA	NA	NA	NA	-5.18139544	0.001079319

LOC110380305	uncharacterized protein LOC110383969	NA	NA	NA	NA	-5.155022778	0.000301203
LOC110383737	uncharacterized protein LOC110377222	NA	NA	NA	NA	-5.126005708	0.000385054
LOC110375807	testis-specific serine/threonine-protein kinase 2	NA	NA	NA	NA	-4.857911348	2.60E-05
LOC110370522	uncharacterized protein C1orf194	NA	NA	NA	NA	-4.853597308	0.000109024
LOC110383952	uncharacterized protein LOC110371428	NA	NA	NA	NA	-4.826336088	0.002062584
LOC110380588	succinate dehydrogenase	NA	NA	NA	NA	-4.756957623	1.66E-05
LOC110384514	serine protease snake-like	NA	NA	NA	NA	-4.748360184	2.46E-08
LOC110375592	coiled-coil domain-containing protein 40	NA	NA	NA	NA	-4.656939993	0.001351671
LOC110378338	uncharacterized protein LOC110370816	NA	NA	NA	NA	-4.564587881	0.00155029
LOC110381615	mitochondrial 2-oxoglutarate/malate carrier protein-like	NA	NA	NA	NA	-4.538445473	6.33E-05
LOC110373622	tektin-4-like	NA	NA	NA	NA	-4.534658777	0.000167652
LOC110382890	decaprenyl-diphosphate synthase subunit 2-like	NA	NA	NA	NA	-4.534382845	0.002398083
LOC110382890	uncharacterized protein LOC110384665	NA	NA	NA	NA	-4.498196522	0.003894707
LOC110371956	uncharacterized protein LOC110383656, partial	NA	NA	NA	NA	-4.481422561	0.002346517

LOC110371956	uncharacterized protein LOC110371816	NA	NA	NA	NA	-4.409064356	0.000132364
LOC110382756	uncharacterized protein LOC110373624	NA	NA	NA	NA	-4.40681628	2.04E-06
LOC110377991	NA	NA	NA	NA	NA	-4.388480889	0.003332435
LOC110373289	proton-coupled amino acid transporter-like protein CG1139	NA	NA	NA	NA	-4.363428067	3.62E-09
LOC110379885	uncharacterized protein LOC110374512	NA	NA	NA	NA	-4.285582346	0.002977979
LOC110373081	uncharacterized protein LOC110372889 isoform X1	NA	NA	NA	NA	-4.242278622	0.000328426
LOC110383213	uncharacterized protein LOC110372889 isoform X2	NA	NA	NA	NA	-4.242278622	0.000328426
LOC110383213	major heat shock 70 kDa protein Ab-like	NA	NA	NA	NA	-4.165810193	4.26E-05
LOC110383213	mitochondrial pyruvate carrier 1-like	NA	NA	NA	NA	-4.05682417	4.60E-05
LOC110382489	uncharacterized protein LOC110378259	NA	NA	NA	NA	-4.048052125	6.42E-05
LOC110380870	solute carrier family 13 member 5-like isoform X3	NA	NA	NA	NA	-4.032155359	1.00E-06
LOC110380870	solute carrier family 13 member 5-like isoform X2	NA	NA	NA	NA	-4.032155359	1.00E-06

LOC110380413	solute carrier family 13 member 5-like isoform X1	NA	NA	NA	NA	-4.032155359	1.00E-06
LOC110378201	uncharacterized protein LOC110378184	NA	NA	NA	NA	-3.998752875	3.99E-08
LOC110382545	WAS/WASL-interacting protein family member 3-like	NA	NA	NA	NA	-3.973345463	0.001060574
LOC110373882	uncharacterized protein LOC110381130	NA	NA	NA	NA	-3.902437264	4.44E-06
LOC110373882	uncharacterized protein LOC110376624	NA	NA	NA	NA	-3.881690952	0.000314745
LOC110373882	glutamate	NA	NA	NA	NA	-3.835576547	0.001892628
LOC110373882	uncharacterized protein LOC110374199	NA	NA	NA	NA	-3.812043479	0.004721512
LOC110373882	uncharacterized protein LOC110382225 isoform X2	NA	NA	NA	NA	-3.79583054	1.66E-05
LOC110374941	uncharacterized protein LOC110382225 isoform X1	NA	NA	NA	NA	-3.79583054	1.66E-05
LOC110380439	uncharacterized protein LOC110382225 isoform X3	NA	NA	NA	NA	-3.79583054	1.66E-05
LOC110377216	uncharacterized protein LOC110370601	NA	NA	NA	NA	-3.790353397	5.37E-07
LOC110383040	serine/arginine repetitive matrix protein 1-like isoform X1	NA	NA	NA	NA	-3.753131136	0.002238272

LOC110378680	serine/arginine repetitive matrix protein 1-like isoform X2	NA	NA	NA	NA	-3.753131136	0.002238272
LOC110373119	uncharacterized protein LOC110384654	NA	NA	NA	NA	-3.684219407	0.004817686
LOC110373119	uncharacterized protein LOC110378119	NA	NA	NA	NA	-3.647858606	2.34E-05
LOC110375790	LOW QUALITY PROTEIN: uncharacterized protein LOC110372172	NA	NA	NA	NA	-3.647119213	0.001648621
LOC110380199	uncharacterized protein LOC110373825 isoform X1	NA	NA	NA	NA	-3.623559271	3.42E-07
LOC110378184	uncharacterized protein LOC110373825 isoform X2	NA	NA	NA	NA	-3.623559271	3.42E-07
LOC110382578	uncharacterized protein LOC110373825 isoform X3	NA	NA	NA	NA	-3.623559271	3.42E-07
LOC110371394	putative nuclease HARBI1	NA	NA	NA	NA	-3.622900817	0.001881687
LOC110371394	ADP/ATP translocase 1-like	NA	NA	NA	NA	-3.617905776	2.16E-05
LOC110370804	NA	NA	NA	NA	NA	-3.603153293	0.000214885
LOC110374557	uncharacterized protein LOC110373473	NA	NA	NA	NA	-3.597680575	0.004588998
LOC110371907	protamine-like isoform X3	NA	NA	NA	NA	-3.576884752	2.53E-05
LOC110383985	protamine-2-like isoform X4	NA	NA	NA	NA	-3.576884752	2.53E-05

LOC110370125	protamine-like isoform X1	NA	NA	NA	NA	-3.576884752	2.53E-05
LOC110373825	protamine-like isoform X2	NA	NA	NA	NA	-3.576884752	2.53E-05
LOC110373825	uncharacterized protein LOC110379359	NA	NA	NA	NA	-3.550404019	4.73E-05
LOC110373825	uncharacterized protein LOC110374876	NA	NA	NA	NA	-3.535483955	3.17E-05
LOC110377379	NA	NA	NA	NA	NA	-3.463800942	0.001572646
LOC110381716	uncharacterized protein LOC110376912 isoform X1	NA	NA	NA	NA	-3.458350929	0.001775313
LOC110377393	uncharacterized protein LOC110376912 isoform X2	NA	NA	NA	NA	-3.458350929	0.001775313
LOC110374160	kallikrein-7-like	NA	NA	NA	NA	-3.44838192	0.000169507
LOC110376175	NA	NA	NA	NA	NA	-3.446705324	0.002438247
LOC110370601	keratin-associated protein 10-4-like	NA	NA	NA	NA	-3.433251762	2.66E-06
LOC110375849	pyruvate kinase-like	NA	NA	NA	NA	-3.429901133	0.002534577
LOC110380762	uncharacterized protein LOC110374870	NA	NA	NA	NA	-3.426754453	0.00022929
LOC110380762	dihydrolipoyllysine-residue succinyltransferase component of 2-oxoglutarate dehydrogenase complex, mitochondrial-like	NA	NA	NA	NA	-3.396800842	0.000132364

LOC110380762	cAMP and cAMP-inhibited cGMP 3\\',5\\'-cyclic phosphodiesterase 10A-like	NA	NA	NA	NA	-3.366888969	0.001768452
LOC110374549	uncharacterized protein LOC110376454 isoform X1	NA	NA	NA	NA	-3.352056818	0.000365632
LOC110374403	uncharacterized protein LOC110376454 isoform X2	NA	NA	NA	NA	-3.352056818	0.000365632
LOC110371672	uncharacterized protein LOC110378349 isoform X1	NA	NA	NA	NA	-3.339023654	0.000183019
LOC110382492	uncharacterized protein LOC110378349 isoform X2	NA	NA	NA	NA	-3.339023654	0.000183019
LOC110380587	mitochondrial 2-oxoglutarate/malate carrier protein-like isoform X1	NA	NA	NA	NA	-3.326237696	0.000313224
LOC110374272	mitochondrial 2-oxoglutarate/malate carrier protein-like isoform X2	NA	NA	NA	NA	-3.326237696	0.000313224
LOC110377447	uncharacterized protein LOC110377976	NA	NA	NA	NA	-3.290192392	3.17E-05
LOC110377447	NA	NA	NA	NA	NA	-3.287382156	0.002683447
LOC110377447	uncharacterized protein LOC110373980	NA	NA	NA	NA	-3.272088027	0.001735353

LOC110377398	uncharacterized protein LOC110373463	NA	NA	NA	NA	-3.252712552	0.000100073
LOC110377398	uncharacterized protein LOC110373892 isoform X3	NA	NA	NA	NA	-3.20666571	1.26E-05
LOC110377398	uncharacterized protein LOC110373892 isoform X2	NA	NA	NA	NA	-3.20666571	1.26E-05
LOC110372872	proteoglycan 4-like isoform X1	NA	NA	NA	NA	-3.20666571	1.26E-05
LOC110372872	uncharacterized protein LOC110373892 isoform X4	NA	NA	NA	NA	-3.20666571	1.26E-05
LOC110373566	nuclear pore complex protein Nup153-like isoform X1	NA	NA	NA	NA	-3.190236336	8.45E-09
LOC110371614	nuclear pore complex protein Nup153-like isoform X1	NA	NA	NA	NA	-3.190236336	8.45E-09
LOC110371614	nuclear pore complex protein Nup153-like isoform X2	NA	NA	NA	NA	-3.190236336	8.45E-09
LOC110372624	nuclear pore complex protein Nup153-like isoform X1	NA	NA	NA	NA	-3.190236336	8.45E-09
LOC110384475	uncharacterized protein LOC110382545	NA	NA	NA	NA	-3.175742222	7.43E-09
LOC110373624	63 kDa chaperonin, mitochondrial	NA	NA	NA	NA	-3.175067254	3.01E-05

LOC110373914	neuronal acetylcholine receptor subunit alpha-5-like	NA	NA	NA	NA	-3.14414855	3.19E-05
LOC110370370	glutamate dehydrogenase, mitochondrial-like	NA	NA	NA	NA	-3.127032403	0.002599103
LOC110372452	organic cation/carnitine transporter 7-like	NA	NA	NA	NA	-3.09173794	7.54E-07
LOC110378771	uncharacterized protein LOC110372316	NA	NA	NA	NA	-3.091077245	0.003162923
LOC110378771	testis-specific serine/threonine-protein kinase 3 isoform X3	NA	NA	NA	NA	-3.088229852	0.004538228
LOC110382743	testis-specific serine/threonine-protein kinase 3 isoform X5	NA	NA	NA	NA	-3.088229852	0.004538228
LOC110384152	testis-specific serine/threonine-protein kinase 3 isoform X4	NA	NA	NA	NA	-3.088229852	0.004538228
LOC110384152	testis-specific serine/threonine-protein kinase 3 isoform X2	NA	NA	NA	NA	-3.088229852	0.004538228
LOC110384152	testis-specific serine/threonine-protein kinase 3 isoform X1	NA	NA	NA	NA	-3.088229852	0.004538228
LOC110384152	testis-specific serine/threonine-protein kinase 3 isoform X6	NA	NA	NA	NA	-3.088229852	0.004538228

LOC110376119	uncharacterized protein LOC110375026	NA	NA	NA	NA	-2.987590883	0.00152734
LOC110381109	UDP-glucuronosyltransferase 2B17-like isoform X1	NA	NA	NA	NA	-2.980621908	8.62E-06
LOC110380906	UDP-glucuronosyltransferase 2B17-like isoform X2	NA	NA	NA	NA	-2.980621908	8.62E-06
LOC110373440	UDP-glucuronosyltransferase 2B19-like isoform X3	NA	NA	NA	NA	-2.980621908	8.62E-06
LOC110373444	uncharacterized protein LOC110381094	NA	NA	NA	NA	-2.971232595	0.000641889
LOC110373457	secretory phospholipase A2 receptor-like, partial	NA	NA	NA	NA	-2.970123677	1.66E-09
LOC110380178	glutathione S-transferase 1-like	NA	NA	NA	NA	-2.966234642	0.003005044
LOC110380178	peroxisomal N(1)-acetyl-spermine/spermidine oxidase-like	NA	NA	NA	NA	-2.949257272	0.002517614
LOC110380419	mono	NA	NA	NA	NA	-2.923416515	0.001627238
LOC110380606	secretory phospholipase A2 receptor-like	NA	NA	NA	NA	-2.905922138	8.00E-07
LOC110370036	uncharacterized protein LOC110376175	NA	NA	NA	NA	-2.905143021	4.64E-07
LOC110380790	uncharacterized protein LOC110373242 isoform X2	NA	NA	NA	NA	-2.898909953	0.000421655

LOC110380962	uncharacterized protein LOC110373242 isoform X1	NA	NA	NA	NA	-2.898909953	0.000421655
LOC110380962	uncharacterized protein LOC110378550 isoform X2	NA	NA	NA	NA	-2.883225249	0.003064121
LOC110381130	uncharacterized protein LOC110378550 isoform X3	NA	NA	NA	NA	-2.883225249	0.003064121
LOC110378552	uncharacterized protein LOC110378550 isoform X4	NA	NA	NA	NA	-2.883225249	0.003064121
LOC110377312	uncharacterized protein LOC110378550 isoform X5	NA	NA	NA	NA	-2.883225249	0.003064121
LOC110384189	uncharacterized protein LOC110378550 isoform X5	NA	NA	NA	NA	-2.883225249	0.003064121
LOC110384189	uncharacterized protein LOC110378550 isoform X1	NA	NA	NA	NA	-2.883225249	0.003064121
LOC110371551	uncharacterized protein LOC110374478	NA	NA	NA	NA	-2.836215055	0.001782194
LOC110375842	otoferlin-like	NA	NA	NA	NA	-2.81695139	0.00364023
LOC110384310	ubiquitin carboxyl-terminal hydrolase 7-like isoform X1	NA	NA	NA	NA	-2.813646378	0.002977979

LOC110372788	ubiquitin carboxyl-terminal hydrolase 7-like isoform X2	NA	NA	NA	NA	-2.813646378	0.002977979
LOC110372788	ubiquitin carboxyl-terminal hydrolase 7-like isoform X1	NA	NA	NA	NA	-2.813646378	0.002977979
LOC110375674	ubiquitin carboxyl-terminal hydrolase 7-like isoform X1	NA	NA	NA	NA	-2.813646378	0.002977979
LOC110375674	ubiquitin carboxyl-terminal hydrolase 7-like isoform X3	NA	NA	NA	NA	-2.813646378	0.002977979
LOC110375674	uncharacterized protein LOC110381643	NA	NA	NA	NA	-2.807797295	0.002581542
LOC110375674	septin-2 isoform X1	NA	NA	NA	NA	-2.806224865	7.24E-06
LOC110382754	septin-2 isoform X2	NA	NA	NA	NA	-2.806224865	7.24E-06
LOC110382754	dynein heavy chain 5, axonemal isoform X1	NA	NA	NA	NA	-2.754034751	6.00E-05
LOC110382754	dynein heavy chain 5, axonemal isoform X2	NA	NA	NA	NA	-2.754034751	6.00E-05
LOC110377896	cytochrome P450 6B5-like	NA	NA	NA	NA	-2.749328186	0.000268929
LOC110373892	uncharacterized protein LOC110378813	NA	NA	NA	NA	-2.714782379	0.001567361
LOC110373892	ommochrome-binding protein-like	NA	NA	NA	NA	-2.688600189	0.002222249
LOC110373892	UDP-glucuronosyltransferase 2B14-like	NA	NA	NA	NA	-2.638273802	3.30E-12

LOC110373892	aldose 1-epimerase-like	NA	NA	NA	NA	-2.637126827	0.003372824
LOC110380698	uncharacterized protein LOC110369700 isoform X2	NA	NA	NA	NA	-2.626704268	0.000397669
LOC110381609	uncharacterized protein LOC110369700 isoform X1	NA	NA	NA	NA	-2.626704268	0.000397669
LOC110381609	uncharacterized protein LOC110377973 isoform X1	NA	NA	NA	NA	-2.580708868	0.004909905
LOC110381609	uncharacterized protein LOC110377973 isoform X2	NA	NA	NA	NA	-2.580708868	0.004909905
LOC110371753	high mobility group protein B1-like isoform X2	NA	NA	NA	NA	-2.506893656	3.28E-21
LOC110369863	high mobility group protein B3-like isoform X1	NA	NA	NA	NA	-2.506893656	3.28E-21
LOC110381433	high mobility group protein B1-like isoform X2	NA	NA	NA	NA	-2.506893656	3.28E-21
LOC110381642	uncharacterized protein LOC110380962 isoform X2	NA	NA	NA	NA	-2.469532366	4.44E-06
LOC110381642	uncharacterized protein LOC110380962 isoform X1	NA	NA	NA	NA	-2.469532366	4.44E-06

LOC110373271	Fanconi anemia group I protein-like	NA	NA	NA	NA	-2.459431071	2.18E-12
LOC110382225	titin-like	NA	NA	NA	NA	-2.455988556	0.000169988
LOC110382225	uncharacterized protein LOC110381137 isoform X1	NA	NA	NA	NA	-2.454064038	0.001445558
LOC110382225	uncharacterized protein LOC110381137 isoform X2	NA	NA	NA	NA	-2.454064038	0.001445558
LOC110376850	LOW QUALITY PROTEIN: uncharacterized protein LOC110381109, partial	NA	NA	NA	NA	-2.452494619	3.20E-06
LOC110372805	UDP-glucuronosyltransferase 2B17-like	NA	NA	NA	NA	-2.45134448	2.45E-05
LOC110372805	LOW QUALITY PROTEIN: sugar transporter ERD6-like 4	NA	NA	NA	NA	-2.448632076	9.32E-07
LOC110372805	uncharacterized protein LOC110376926	NA	NA	NA	NA	-2.445659913	2.14E-05
LOC110372805	myosin-2 heavy chain-like isoform X1	NA	NA	NA	NA	-2.413979428	4.70E-05
LOC110372805	myosin-2 heavy chain-like isoform X2	NA	NA	NA	NA	-2.413979428	4.70E-05
LOC110376926	myosin-7-like isoform X5	NA	NA	NA	NA	-2.402406699	0.00079107
LOC110373034	myosin-7-like isoform X2	NA	NA	NA	NA	-2.402406699	0.00079107
LOC110384309	myosin-7-like isoform X3	NA	NA	NA	NA	-2.402406699	0.00079107
LOC110378119	myosin-7-like isoform X4	NA	NA	NA	NA	-2.402406699	0.00079107
LOC110377183	myosin-7-like isoform X1	NA	NA	NA	NA	-2.402406699	0.00079107

LOC110382752	uncharacterized protein LOC110369613	NA	NA	NA	NA	-2.388083991	0.00054607
LOC110369792	facilitated trehalose transporter Tret1-like isoform X2	NA	NA	NA	NA	-2.35736184	4.10E-08
LOC110380185	facilitated trehalose transporter Tret1-like isoform X1	NA	NA	NA	NA	-2.35736184	4.10E-08
LOC110382327	NADP-dependent malic enzyme-like	NA	NA	NA	NA	-2.356336156	0.00155029
LOC110382327	uncharacterized protein LOC110375008	NA	NA	NA	NA	-2.3398373	0.002919786
LOC110382327	probable cytochrome P450 301a1, mitochondrial	NA	NA	NA	NA	-2.331485372	0.002222249
LOC110382327	pregnancy zone protein-like	NA	NA	NA	NA	-2.315231422	0.000950836
LOC110373500	uncharacterized protein DDB_G0284459-like isoform X2	NA	NA	NA	NA	-2.314376333	0.000132364
LOC110379053	uncharacterized protein DDB_G0284459-like isoform X1	NA	NA	NA	NA	-2.314376333	0.000132364
LOC110374682	uncharacterized protein DDB_G0284459-like isoform X3	NA	NA	NA	NA	-2.314376333	0.000132364
LOC110382370	brachyurin-like	NA	NA	NA	NA	-2.301738869	1.09E-12
LOC110381119	NA	NA	NA	NA	NA	-2.242926733	0.000530148

LOC110374636	uncharacterized protein LOC110384189 isoform X2	NA	NA	NA	NA	-2.223241013	5.89E-06
LOC110374636	uncharacterized protein LOC110384189 isoform X1	NA	NA	NA	NA	-2.223241013	5.89E-06
LOC110379119	uncharacterized protein LOC110381629	NA	NA	NA	NA	-2.189375447	0.002461655
LOC110370737	uncharacterized protein LOC110378563	NA	NA	NA	NA	-2.187906932	0.002217304
LOC110381762	regucalcin-like	NA	NA	NA	NA	-2.18230287	6.38E-05
LOC110374876	GILT-like protein 1	NA	NA	NA	NA	-2.174565606	0.002438247
LOC110377976	luciferin 4-monooxygenase-like	NA	NA	NA	NA	-2.167968049	2.17E-12
LOC110384095	uncharacterized protein LOC110376913	NA	NA	NA	NA	-2.151334161	0.000236099
LOC110373418	cytochrome P450 6l1-like	NA	NA	NA	NA	-2.119319641	7.35E-14
LOC110371978	methyltransferase-like protein 6	NA	NA	NA	NA	-2.1168318	2.70E-05
LOC110372020	proteoglycan Cow	NA	NA	NA	NA	-2.108624207	0.004992002
LOC110371748	UDP-glucuronosyltransferase 2B10-like	NA	NA	NA	NA	-2.090751783	1.14E-11
LOC110376455	cholinesterase 1-like	NA	NA	NA	NA	-2.078629895	3.75E-21
LOC110381494	paramyosin-like	NA	NA	NA	NA	-2.06865056	0.000366664
LOC110381494	high affinity copper uptake protein 1-like	NA	NA	NA	NA	-2.059779935	4.78E-12
LOC110375099	high affinity copper uptake protein 1-like	NA	NA	NA	NA	-2.059779935	4.78E-12

LOC110375101	UDP-glucuronosyltransferase 2B13-like	NA	NA	NA	NA	-2.059134183	1.70E-05
LOC110371042	uncharacterized protein LOC110369643 isoform X2	NA	NA	NA	NA	-2.019189234	0.000132364
LOC110371042	uncharacterized protein LOC110369643 isoform X1	NA	NA	NA	NA	-2.019189234	0.000132364
LOC110378776	cytochrome P450 6B5-like	NA	NA	NA	NA	-2.017988033	0.000764853
LOC110379359	trypsin, alkaline C-like	NA	NA	NA	NA	-2.013123353	5.25E-13
LOC110383666	uncharacterized protein LOC110379213	NA	NA	NA	NA	-2.000076962	0.003145329
LOC110373001	uncharacterized protein LOC110382578	NA	NA	NA	NA	-1.984979883	3.99E-08
LOC110373001	trypsin inhibitor-like	NA	NA	NA	NA	-1.921601015	0.00020625
LOC110379857	secretory phospholipase A2 receptor-like	NA	NA	NA	NA	-1.879392978	0.001752584
LOC110373062	NA	NA	NA	NA	NA	-1.864242648	5.39E-06
LOC110373062	uncharacterized protein LOC110382220 isoform X1	NA	NA	NA	NA	-1.82899937	0.000726069
LOC110380203	uncharacterized protein LOC110382220 isoform X3	NA	NA	NA	NA	-1.82899937	0.000726069
LOC110373251	uncharacterized protein LOC110382220 isoform X3	NA	NA	NA	NA	-1.82899937	0.000726069

LOC110370749	uncharacterized protein LOC110382220 isoform X2	NA	NA	NA	NA	-1.82899937	0.000726069
LOC110377194	putative carbonic anhydrase 5	NA	NA	NA	NA	-1.805287566	0.00011775
LOC110380201	acidic amino acid decarboxylase GADL1	NA	NA	NA	NA	-1.765580687	1.98E-08
LOC110380201	NA	NA	NA	NA	NA	-1.760941511	6.87E-05
LOC110380201	putative inorganic phosphate cotransporter	NA	NA	NA	NA	-1.743004273	0.003706063
LOC110378259	fatty acid-binding protein 2-like	NA	NA	NA	NA	-1.736530899	3.31E-06
LOC110371455	uncharacterized protein LOC110374087	NA	NA	NA	NA	-1.706464225	0.001241569
LOC110376059	high affinity copper uptake protein 1-like	NA	NA	NA	NA	-1.666864355	2.05E-17
LOC110371244	high affinity copper uptake protein 1-like	NA	NA	NA	NA	-1.666864355	2.05E-17
LOC110384365	inactive hydroxysteroid dehydrogenase-like protein 1	NA	NA	NA	NA	-1.64169493	4.12E-06
LOC110384365	trans-1,2-dihydrobenzene-1,2-diol dehydrogenase-like	NA	NA	NA	NA	-1.632075019	0.000167652
LOC110370170	paired box protein Pax-1-like	NA	NA	NA	NA	-1.618774475	3.42E-07
LOC110370170	esterase B1-like	NA	NA	NA	NA	-1.61574614	6.00E-05

LOC110380903	peroxisomal N(1)-acetyl-spermine/spermidine oxidase-like isoform X2	NA	NA	NA	NA	-1.595159678	2.98E-17
LOC110376833	peroxisomal N(1)-acetyl-spermine/spermidine oxidase-like isoform X7	NA	NA	NA	NA	-1.595159678	2.98E-17
LOC110379855	peroxisomal N(1)-acetyl-spermine/spermidine oxidase-like isoform X4	NA	NA	NA	NA	-1.595159678	2.98E-17
LOC110371313	peroxisomal N(1)-acetyl-spermine/spermidine oxidase-like isoform X3	NA	NA	NA	NA	-1.595159678	2.98E-17
LOC110377897	peroxisomal N(1)-acetyl-spermine/spermidine oxidase-like isoform X1	NA	NA	NA	NA	-1.595159678	2.98E-17
LOC110373463	peroxisomal N(1)-acetyl-spermine/spermidine oxidase-like isoform X7	NA	NA	NA	NA	-1.595159678	2.98E-17
LOC110383019	peroxisomal N(1)-acetyl-spermine/spermidine oxidase-like isoform X5	NA	NA	NA	NA	-1.595159678	2.98E-17
LOC110374791	peroxisomal N(1)-acetyl-spermine/spermidine oxidase-like isoform X6	NA	NA	NA	NA	-1.595159678	2.98E-17
LOC110378001	protein lethal(2)essential for life-like	NA	NA	NA	NA	-1.589827773	2.25E-06

LOC110376139	multiple inositol polyphosphate phosphatase 1-like	NA	NA	NA	NA	-1.534435	0.000935839
LOC110369643	neuroglian-like	NA	NA	NA	NA	-1.51826385	0.001302381
LOC110369643	probable sulfite oxidase, mitochondrial isoform X2	NA	NA	NA	NA	-1.483824913	6.58E-17
LOC110371816	probable sulfite oxidase, mitochondrial isoform X3	NA	NA	NA	NA	-1.483824913	6.58E-17
LOC110377275	probable sulfite oxidase, mitochondrial isoform X1	NA	NA	NA	NA	-1.483824913	6.58E-17
LOC110377275	uncharacterized protein LOC110377859	NA	NA	NA	NA	-1.481456948	0.001161383
LOC110377275	centrosomal protein of 290 kDa-like	NA	NA	NA	NA	-1.466400513	0.004147885
LOC110382131	uncharacterized protein LOC110370334	NA	NA	NA	NA	-1.454784292	0.000609021
LOC110383757	glutathione S-transferase 1-like isoform X2	NA	NA	NA	NA	-1.450550112	2.66E-06
LOC110384440	glutathione S-transferase 1-like isoform X1	NA	NA	NA	NA	-1.450550112	2.66E-06
LOC110384440	glutathione S-transferase 1-like isoform X3	NA	NA	NA	NA	-1.450550112	2.66E-06
LOC110376915	glutathione S-transferase 1-like isoform X4	NA	NA	NA	NA	-1.450550112	2.66E-06
LOC110377335	cationic amino acid transporter 2-like isoform X1	NA	NA	NA	NA	-1.44548587	4.12E-06

LOC110384153	cationic amino acid transporter 2-like isoform X2	NA	NA	NA	NA	-1.44548587	4.12E-06
LOC110379956	lipase 3-like	NA	NA	NA	NA	-1.445159398	0.002222249
LOC110374139	carotenoid isomerooxygenase isoform X2	NA	NA	NA	NA	-1.443041872	0.000133231
LOC110377790	carotenoid isomerooxygenase isoform X1	NA	NA	NA	NA	-1.443041872	0.000133231
LOC110381513	NA	NA	NA	NA	NA	-1.427668249	0.00028438
LOC110369711	trypsin, alkaline B-like	NA	NA	NA	NA	-1.424809597	0.0004451
LOC110382814	long-chain-fatty-acid--CoA ligase 5 isoform X2	NA	NA	NA	NA	-1.40557619	0.000863713
LOC110384673	long-chain-fatty-acid--CoA ligase 5 isoform X1	NA	NA	NA	NA	-1.40557619	0.000863713
LOC110373646	long-chain-fatty-acid--CoA ligase 5 isoform X1	NA	NA	NA	NA	-1.40557619	0.000863713
LOC110378349	long-chain-fatty-acid--CoA ligase 5 isoform X3	NA	NA	NA	NA	-1.40557619	0.000863713
LOC110378349	long-chain-fatty-acid--CoA ligase 5 isoform X1	NA	NA	NA	NA	-1.40557619	0.000863713
LOC110372451	facilitated trehalose transporter Tret1-like	NA	NA	NA	NA	-1.402888577	0.004721512
LOC110379983	NA	NA	NA	NA	NA	-1.388327191	0.00075503
LOC110384677	apoptosis-inducing factor 1, mitochondrial-like	NA	NA	NA	NA	-1.371992205	0.001280943

LOC110373702	ATP-dependent DNA helicase 2 subunit 1	NA	NA	NA	NA	-1.333061148	0.000325235
LOC110382090	cytochrome P450 6B6-like	NA	NA	NA	NA	-1.332585877	7.35E-14
LOC110374194	4-coumarate--CoA ligase 1-like	NA	NA	NA	NA	-1.326415348	1.57E-05
LOC110377338	glutathione S-transferase 1-like	NA	NA	NA	NA	-1.322227842	0.000147116
LOC110378942	ethanolaminephosphotransferase 1-like	NA	NA	NA	NA	-1.303866499	0.001687261
LOC110381554	juvenile hormone epoxide hydrolase-like	NA	NA	NA	NA	-1.301445084	0.000100128
LOC110377917	probable fatty acid-binding protein	NA	NA	NA	NA	-1.297095657	0.001735353
LOC110377917	cytochrome P450 6l1-like	NA	NA	NA	NA	-1.2775755	0.000983503
LOC110377917	4-coumarate--CoA ligase 1-like	NA	NA	NA	NA	-1.263206122	2.50E-05
LOC110374870	uncharacterized protein LOC110376079 isoform X2	NA	NA	NA	NA	-1.251586436	0.002222249
LOC110381422	uncharacterized protein LOC110376079 isoform X1	NA	NA	NA	NA	-1.251586436	0.002222249
LOC110381422	cytochrome P450 6B6-like	NA	NA	NA	NA	-1.246677851	4.12E-06
LOC110381422	peroxisomal membrane protein 11C-like	NA	NA	NA	NA	-1.244573191	0.000790767
LOC110381422	retinol dehydrogenase 12-like	NA	NA	NA	NA	-1.241941403	2.60E-05

LOC110381422	cholinesterase 1-like	NA	NA	NA	NA	-1.223766458	6.85E-05
LOC110381422	UDP-glucuronosyltransferase 2C1-like	NA	NA	NA	NA	-1.218732095	0.001110739
LOC110381422	cytochrome P450 4C1-like	NA	NA	NA	NA	-1.218031413	0.000215519
LOC110381422	DNA repair and recombination protein RAD54-like	NA	NA	NA	NA	-1.211168392	0.002023184
LOC110381422	luciferin 4-monooxygenase-like	NA	NA	NA	NA	-1.209019169	6.28E-05
LOC110372509	delta-1-pyrroline-5-carboxylate synthase	NA	NA	NA	NA	-1.205616312	0.001241569
LOC110372509	replication protein A 32 kDa subunit	NA	NA	NA	NA	-1.204421314	0.000101831
LOC110372509	sterol O-acyltransferase 2	NA	NA	NA	NA	-1.183862728	7.45E-06
LOC110376913	sterol O-acyltransferase 2	NA	NA	NA	NA	-1.183862728	7.45E-06
LOC110377750	sterol O-acyltransferase 2	NA	NA	NA	NA	-1.183862728	7.45E-06
LOC110371736	carboxypeptidase B-like	NA	NA	NA	NA	-1.183649804	1.56E-05
LOC110378575	b(0,+)-type amino acid transporter 1-like isoform X1	NA	NA	NA	NA	-1.165078026	0.000231012
LOC110380898	b(0,+)-type amino acid transporter 1-like isoform X1	NA	NA	NA	NA	-1.165078026	0.000231012

LOC110373446	b(0,+)-type amino acid transporter 1-like isoform X2	NA	NA	NA	NA	-1.165078026	0.000231012
LOC110375628	small G protein signaling modulator 3 homolog	NA	NA	NA	NA	-1.161896659	2.77E-05
LOC110378355	F-BAR and double SH3 domains protein 2 isoform X1	NA	NA	NA	NA	-1.159213664	0.001030157
LOC110376752	F-BAR and double SH3 domains protein 2 isoform X3	NA	NA	NA	NA	-1.159213664	0.001030157
LOC110379762	F-BAR and double SH3 domains protein 2 isoform X2	NA	NA	NA	NA	-1.159213664	0.001030157
LOC110383969	F-BAR and double SH3 domains protein 2 isoform X4	NA	NA	NA	NA	-1.159213664	0.001030157
LOC110372813	facilitated trehalose transporter Tret1-2 homolog	NA	NA	NA	NA	-1.156803633	0.003483132
LOC110373755	esterase FE4-like	NA	NA	NA	NA	-1.155475929	0.000313224
LOC110373755	cytochrome b5-related protein-like	NA	NA	NA	NA	-1.151146476	0.001405392
LOC110373755	uncharacterized protein LOC110372334	NA	NA	NA	NA	-1.127621439	0.004323649
LOC110373755	glucosylceramidase-like	NA	NA	NA	NA	-1.119150975	1.14E-07
LOC110373755	cytochrome P450 4d2-like	NA	NA	NA	NA	-1.113697677	3.51E-07

LOC110370672	venom carboxylesterase-6-like	NA	NA	NA	NA	-1.109379198	3.80E-05
LOC110374972	zinc transporter 1-like	NA	NA	NA	NA	-1.10344744	8.30E-10
LOC110377761	zinc transporter 1-like	NA	NA	NA	NA	-1.10344744	8.30E-10
LOC110371295	zinc transporter 1-like	NA	NA	NA	NA	-1.10344744	8.30E-10
LOC110373207	glutamine synthetase 2 cytoplasmic-like	NA	NA	NA	NA	-1.10244257	0.000197581
LOC110373207	trypsin CFT-1-like	NA	NA	NA	NA	-1.097557377	0.000313224
LOC110374597	NA	NA	NA	NA	NA	-1.097423842	0.001995342
LOC110378565	seipin isoform X2	NA	NA	NA	NA	-1.089862237	0.002212869
LOC110379933	seipin isoform X1	NA	NA	NA	NA	-1.089862237	0.002212869
LOC110376624	zinc finger protein 14-like	NA	NA	NA	NA	-1.085846838	2.94E-07
LOC110383126	aminoacylase-1-like	NA	NA	NA	NA	-1.072240959	6.38E-05
LOC110371257	alpha-(1,3)-fucosyltransferase 9-like	NA	NA	NA	NA	-1.070311769	2.45E-05
LOC110374925	venom dipeptidyl peptidase 4-like isoform X1	NA	NA	NA	NA	-1.06946905	1.02E-06
LOC110372889	venom dipeptidyl peptidase 4-like isoform X1	NA	NA	NA	NA	-1.06946905	1.02E-06
LOC110372889	venom dipeptidyl peptidase 4-like isoform X2	NA	NA	NA	NA	-1.06946905	1.02E-06
LOC110378667	alpha-tocopherol transfer protein-like	NA	NA	NA	NA	-1.055565898	0.000924345
LOC110376388	protein lethal(2)essential for life-like	NA	NA	NA	NA	-1.053436432	0.000424394

LOC110377204	probable alpha-aspartyl dipeptidase	NA	NA	NA	NA	-1.047625974	3.44E-08
LOC110369661	probable alpha-aspartyl dipeptidase	NA	NA	NA	NA	-1.047625974	3.44E-08
LOC110376454	uncharacterized protein LOC110375790	NA	NA	NA	NA	-1.039778304	3.44E-08
LOC110376454	cytochrome P450 6B6-like	NA	NA	NA	NA	-1.031480079	2.59E-09
LOC110374802	lipoyltransferase 1, mitochondrial isoform X1	NA	NA	NA	NA	-1.030628516	0.000238051
LOC110379210	lipoyltransferase 1, mitochondrial isoform X2	NA	NA	NA	NA	-1.030628516	0.000238051
LOC110371954	glycine N-methyltransferase	NA	NA	NA	NA	-1.025843839	0.000210625
LOC110371122	NA	NA	NA	NA	NA	-1.020557139	0.000863713
LOC110372927	facilitated trehalose transporter Tret1-like	NA	NA	NA	NA	-1.017061678	0.004659813
LOC110377222	calphotin-like	NA	NA	NA	NA	-1.015219421	0.001447383
LOC110369700	zinc transporter ZIP10	NA	NA	NA	NA	-1.011025042	0.004000639
LOC110369700	riboflavin kinase	NA	NA	NA	NA	-1.001840234	8.07E-05
LOC110375085	oleosin-B3-like	NA	NA	NA	NA	1.010233505	0.003108599
LOC110373484	uncharacterized protein LOC110374597	NA	NA	NA	NA	1.010467342	0.000313224
LOC110373484	gamma-glutamyltranspeptidase 1-like isoform X2	NA	NA	NA	NA	1.017486127	1.94E-05

LOC110370389	gamma-glutamyltranspeptidase 1-like isoform X2	NA	NA	NA	NA	1.017486127	1.94E-05
LOC110374758	gamma-glutamyltranspeptidase 1-like isoform X3	NA	NA	NA	NA	1.017486127	1.94E-05
LOC110371298	gamma-glutamyltranspeptidase 1-like isoform X2	NA	NA	NA	NA	1.017486127	1.94E-05
LOC110374092	gamma-glutamyltranspeptidase 1-like isoform X1	NA	NA	NA	NA	1.017486127	1.94E-05
LOC110379681	serine protease inhibitor 88Ea-like isoform X1	NA	NA	NA	NA	1.037371484	1.57E-05
LOC110379681	serine protease inhibitor 88Ea-like isoform X2	NA	NA	NA	NA	1.037371484	1.57E-05
LOC110379681	uncharacterized protein LOC110376388	NA	NA	NA	NA	1.04914466	0.000341121
LOC110381359	trehalase isoform X1	NA	NA	NA	NA	1.056379095	0.003063828
LOC110377251	trehalase isoform X1	NA	NA	NA	NA	1.056379095	0.003063828
LOC110372136	trehalase isoform X2	NA	NA	NA	NA	1.056379095	0.003063828
LOC110372136	trehalase isoform X4	NA	NA	NA	NA	1.056379095	0.003063828
LOC110371619	trehalase isoform X3	NA	NA	NA	NA	1.056379095	0.003063828
LOC110375008	probable cytochrome P450 304a1	NA	NA	NA	NA	1.104849077	2.85E-07
LOC110371408	E3 ubiquitin-protein ligase siah2-like	NA	NA	NA	NA	1.108704034	0.000171936

LOC110371408	uncharacterized protein LOC110370170 isoform X2	NA	NA	NA	NA	1.114963911	7.38E-05
LOC110371408	uncharacterized protein LOC110370170 isoform X1	NA	NA	NA	NA	1.114963911	7.38E-05
LOC110371408	brain tumor protein isoform X3	NA	NA	NA	NA	1.116225556	0.001280943
LOC110371408	brain tumor protein isoform X2	NA	NA	NA	NA	1.116225556	0.001280943
LOC110374512	brain tumor protein isoform X2	NA	NA	NA	NA	1.116225556	0.001280943
LOC110377912	brain tumor protein isoform X1	NA	NA	NA	NA	1.116225556	0.001280943
LOC110379944	stearoyl-CoA desaturase 5	NA	NA	NA	NA	1.118090234	1.65E-06
LOC110379944	uncharacterized protein LOC110373453	NA	NA	NA	NA	1.127120455	0.004514791
LOC110379944	uncharacterized protein LOC110381433	NA	NA	NA	NA	1.146433386	1.57E-05
LOC110378550	WD repeat-containing protein 75-like	NA	NA	NA	NA	1.15252528	0.000366664
LOC110378550	mitochondrial import inner membrane translocase subunit Tim29	NA	NA	NA	NA	1.154972835	2.33E-06
LOC110378550	uncharacterized protein LOC110372872 isoform X2	NA	NA	NA	NA	1.159287023	1.20E-06

LOC110378550	uncharacterized protein LOC110372872 isoform X1	NA	NA	NA	NA	1.159287023	1.20E-06
LOC110378550	phospholipid phosphatase 2-like isoform X1	NA	NA	NA	NA	1.169297436	0.00192378
LOC110378550	phospholipid phosphatase 2-like isoform X2	NA	NA	NA	NA	1.169297436	0.00192378
LOC110376049	trypsin, alkaline C-like	NA	NA	NA	NA	1.169803415	8.57E-07
LOC110376049	probable ATP-dependent RNA helicase YTHDC2	NA	NA	NA	NA	1.18056138	0.0012598
LOC110376049	insecticyanin-A-like	NA	NA	NA	NA	1.196956152	4.26E-05
LOC110376049	mitochondrial pyruvate carrier 1-like	NA	NA	NA	NA	1.206709087	0.000848454
LOC110376049	protein mothers against dpp	NA	NA	NA	NA	1.208799523	0.000808509
LOC110373321	probable ATP-dependent RNA helicase DDX43	NA	NA	NA	NA	1.209825111	0.000344984
LOC110379213	esterase FE4-like	NA	NA	NA	NA	1.210228924	0.003305182
LOC110372316	attacin-E-like	NA	NA	NA	NA	1.215449014	0.000863713
LOC110372790	adiponectin receptor protein	NA	NA	NA	NA	1.221485928	4.07E-07
LOC110372790	uncharacterized protein LOC110374972	NA	NA	NA	NA	1.231324687	0.000311859
LOC110372790	serine/threonine-protein kinase pelle-like	NA	NA	NA	NA	1.251181947	0.000549177

LOC110372790	uncharacterized protein LOC110377829 isoform X4	NA	NA	NA	NA	1.251551185	0.003596383
LOC110379022	uncharacterized protein LOC110377829 isoform X2	NA	NA	NA	NA	1.251551185	0.003596383
LOC110376163	uncharacterized protein LOC110377829 isoform X1	NA	NA	NA	NA	1.251551185	0.003596383
LOC110372063	uncharacterized protein LOC110377829 isoform X1	NA	NA	NA	NA	1.251551185	0.003596383
LOC110380745	uncharacterized protein LOC110377829 isoform X3	NA	NA	NA	NA	1.251551185	0.003596383
LOC110377104	cytochrome P450 6a2-like	NA	NA	NA	NA	1.254873663	4.44E-06
LOC110380230	elongation factor-like GTPase 1	NA	NA	NA	NA	1.261516653	0.002217304
LOC110378185	uncharacterized protein LOC110371834	NA	NA	NA	NA	1.26667306	0.002163856
LOC110382761	U3 small nucleolar RNA-associated protein 6 homolog	NA	NA	NA	NA	1.276553772	3.65E-05
LOC110374988	embryonic polarity protein dorsal-like	NA	NA	NA	NA	1.289527179	0.002517614
LOC110382162	sialin	NA	NA	NA	NA	1.341972069	0.000858142

LOC110382162	iron-regulated transcriptional activator AFT2-like	NA	NA	NA	NA	1.344186306	0.000382693
LOC110377265	uncharacterized protein LOC110378771	NA	NA	NA	NA	1.35174497	2.45E-06
LOC110377829	uncharacterized protein LOC110378771	NA	NA	NA	NA	1.35174497	2.45E-06
LOC110377829	uncharacterized protein LOC110377897	NA	NA	NA	NA	1.361061767	9.23E-05
LOC110377829	leishmanolysin-like peptidase	NA	NA	NA	NA	1.366571794	0.001313408
LOC110377829	neuroguidin	NA	NA	NA	NA	1.379198381	0.000374478
LOC110377829	uncharacterized protein LOC110377265	NA	NA	NA	NA	1.385548862	0.003524913
LOC110373810	uncharacterized protein LOC110373537 isoform X3	NA	NA	NA	NA	1.388930286	0.003643587
LOC110376967	uncharacterized protein LOC110373537 isoform X1	NA	NA	NA	NA	1.388930286	0.003643587
LOC110378029	uncharacterized protein LOC110373537 isoform X2	NA	NA	NA	NA	1.388930286	0.003643587
LOC110372422	uncharacterized protein LOC110373537 isoform X3	NA	NA	NA	NA	1.388930286	0.003643587
LOC110373002	uncharacterized protein LOC110384310	NA	NA	NA	NA	1.395642301	6.33E-06
LOC110373537	NA	NA	NA	NA	NA	1.396028909	0.001230502

LOC110373537	leucine zipper putative tumor suppressor 2 homolog isoform X1	NA	NA	NA	NA	1.39727039	6.15E-05
LOC110373537	leucine zipper putative tumor suppressor 2 homolog isoform X2	NA	NA	NA	NA	1.39727039	6.15E-05
LOC110373537	uncharacterized protein LOC110370901	NA	NA	NA	NA	1.405279755	0.001391832
LOC110376148	esterase B1-like	NA	NA	NA	NA	1.412653248	1.26E-08
LOC110373758	uncharacterized protein LOC110376752	NA	NA	NA	NA	1.417884847	0.000301203
LOC110375910	F-box/WD repeat-containing protein 11, partial	NA	NA	NA	NA	1.470244099	0.000210411
LOC110379715	uncharacterized protein LOC110381494 isoform X1	NA	NA	NA	NA	1.472066677	4.59E-05
LOC110373534	uncharacterized protein LOC110381494 isoform X2	NA	NA	NA	NA	1.472066677	4.59E-05
LOC110372024	anillin-like	NA	NA	NA	NA	1.473132984	0.000712832
LOC110382943	NA	NA	NA	NA	NA	1.508953817	0.002160798
LOC110382943	uncharacterized protein LOC110383126	NA	NA	NA	NA	1.515524446	0.000317414
LOC110384665	uncharacterized protein LOC110373446	NA	NA	NA	NA	1.515624687	0.0002822
LOC110376952	uncharacterized protein LOC110373566	NA	NA	NA	NA	1.524030289	1.47E-06

LOC110383125	RNA helicase Mov10l1-like	NA	NA	NA	NA	1.550937505	1.31E-08
LOC110383125	leucine-rich repeat-containing G-protein coupled receptor 5	NA	NA	NA	NA	1.568228996	3.42E-07
LOC110383125	peroxisomal acyl-coenzyme A oxidase 3	NA	NA	NA	NA	1.581484296	0.000169507
LOC110377314	neural/ectodermal development factor IMP-L2-like	NA	NA	NA	NA	1.583601728	0.001227934
LOC110380574	neural/ectodermal development factor IMP-L2-like	NA	NA	NA	NA	1.583601728	0.001227934
LOC110376460	microvitellogenin-like	NA	NA	NA	NA	1.639777825	0.002309253
LOC110376460	uncharacterized protein LOC110380790	NA	NA	NA	NA	1.647211687	4.44E-06
LOC110376460	solute carrier family 22 member 5-like	NA	NA	NA	NA	1.659423193	0.000225124
LOC110382956	solute carrier family 22 member 5-like	NA	NA	NA	NA	1.659423193	0.000225124
LOC110376169	solute carrier family 22 member 5-like	NA	NA	NA	NA	1.659423193	0.000225124
LOC110376169	cytochrome P450 6B2-like	NA	NA	NA	NA	1.664627549	1.32E-07
LOC110376738	uncharacterized protein LOC110382370	NA	NA	NA	NA	1.678276122	2.70E-05
LOC110376738	uncharacterized protein LOC110372624	NA	NA	NA	NA	1.701926694	1.69E-06
LOC110378137	NA	NA	NA	NA	NA	1.711356551	0.003784138

LOC110370841	LOW QUALITY PROTEIN: hemolin-like	NA	NA	NA	NA	1.761497011	1.34E-05
LOC110382299	uncharacterized protein LOC110376119	NA	NA	NA	NA	1.787119099	2.85E-06
LOC110373858	juvenile hormone esterase-like	NA	NA	NA	NA	1.797008724	1.92E-06
LOC110373858	LOW QUALITY PROTEIN: uncharacterized protein LOC110379596	NA	NA	NA	NA	1.80692756	0.000458726
LOC110373858	uncharacterized protein LOC110375849	NA	NA	NA	NA	1.819759725	5.37E-07
LOC110373858	aromatic-L-amino-acid decarboxylase	NA	NA	NA	NA	1.820017762	5.21E-12
LOC110372334	aromatic-L-amino-acid decarboxylase	NA	NA	NA	NA	1.820017762	5.21E-12
LOC110383442	uncharacterized protein LOC110377991	NA	NA	NA	NA	1.820966347	8.80E-11
LOC110373453	cholinesterase 1-like	NA	NA	NA	NA	1.845310949	0.001313408
LOC110371237	uncharacterized protein LOC110381554	NA	NA	NA	NA	1.850517899	0.000223817
LOC110371237	uncharacterized protein LOC110373444	NA	NA	NA	NA	1.904026711	4.12E-06
LOC110371237	eukaryotic translation initiation factor 4E-1A-like	NA	NA	NA	NA	1.93762262	6.42E-07
LOC110371237	NA	NA	NA	NA	NA	1.953424171	6.73E-10
LOC110371237	beta-1,4-mannosyltransferase egh	NA	NA	NA	NA	1.956850481	5.83E-06

LOC110371237	uncharacterized protein K02A2.6-like	NA	NA	NA	NA	1.957763802	0.000160025
LOC110371237	alcohol dehydrogenase-like	NA	NA	NA	NA	1.985228033	2.25E-05
LOC110383195	NA	NA	NA	NA	NA	2.067499165	0.000859765
LOC110373473	uncharacterized protein LOC110373755 isoform X3	NA	NA	NA	NA	2.103996171	0.000303074
LOC110371572	uncharacterized protein LOC110373755 isoform X2	NA	NA	NA	NA	2.103996171	0.000303074
LOC110380956	uncharacterized protein LOC110373755 isoform X1	NA	NA	NA	NA	2.103996171	0.000303074
LOC110370548	uncharacterized protein LOC110373755 isoform X4	NA	NA	NA	NA	2.103996171	0.000303074
LOC110370548	uncharacterized protein LOC110373755 isoform X5	NA	NA	NA	NA	2.103996171	0.000303074
LOC110370548	uncharacterized protein LOC110371455	NA	NA	NA	NA	2.10948996	6.72E-05
LOC110370548	caspase-1-like	NA	NA	NA	NA	2.135394459	6.28E-06
LOC110374199	cyclin-dependent kinase 9 isoform X2	NA	NA	NA	NA	2.163568749	2.29E-09
LOC110375119	cyclin-dependent kinase 9 isoform X1	NA	NA	NA	NA	2.163568749	2.29E-09
LOC110375119	uncharacterized protein LOC110373440	NA	NA	NA	NA	2.209869407	4.12E-06

LOC110377633	apyrase-like	NA	NA	NA	NA	2.554134268	7.96E-05
LOC110379292	glutathione S-transferase 1-like	NA	NA	NA	NA	2.567740226	2.03E-10
LOC110379225	zonadhesin	NA	NA	NA	NA	2.620605184	7.96E-07
LOC110383973	uncharacterized protein LOC110371485	NA	NA	NA	NA	3.018042621	3.13E-32
LOC110384654	rhomboid-related protein 2 isoform X1	NA	NA	NA	NA	3.636913985	0.004147885
LOC110371837	rhomboid-related protein 3 isoform X2	NA	NA	NA	NA	3.636913985	0.004147885
LOC110377973	NA	NA	NA	NA	NA	3.8723004	3.00E-30
LOC110377973	uncharacterized protein LOC110379885	NA	NA	NA	NA	3.915750263	3.61E-10
LOC110378325	uncharacterized protein LOC110383737	NA	NA	NA	NA	3.934604304	1.68E-16
LOC110383998	uncharacterized protein LOC110375807	NA	NA	NA	NA	3.997046171	5.76E-14
LOC110383208	NA	NA	NA	NA	NA	6.966946512	0.000295039
LOC110376001	peroxidase-like	2.636265329	0.022408152	NA	NA	NA	NA
LOC110377042	collagenase-like	2.321907442	0.020938003	NA	NA	NA	NA
LOC110383668	transmembrane protease serine 9-like	2.194967561	4.94E-06	NA	NA	NA	NA
LOC110384488	brachyurin-like	2.182138127	0.04823475	NA	NA	NA	NA
LOC110370329	circadian clock-controlled protein-like	2.092762716	0.031718602	NA	NA	NA	NA
LOC110370021	probable serine hydrolase	1.963415278	0.023289563	NA	NA	NA	NA

LOC110383548	trypsin, alkaline C-like isoform X1	1.865653998	0.000129783	NA	NA	NA	NA
LOC110383548	trypsin CFT-1-like isoform X3	1.865653998	0.000129783	NA	NA	NA	NA
LOC110383548	trypsin CFT-1-like isoform X2	1.865653998	0.000129783	NA	NA	NA	NA
LOC110383666	trypsin, alkaline C-like	1.839577789	6.99E-20	NA	NA	NA	NA
LOC110384095	neuronal acetylcholine receptor subunit alpha-5-like	1.680836192	0.04978224	NA	NA	NA	NA
LOC110380584	trypsin, alkaline C-like	1.667637955	0.00156718	NA	NA	NA	NA
LOC110377047	serine protease 3-like isoform X3	1.664854387	6.64E-05	NA	NA	NA	NA
LOC110377047	serine protease 3-like isoform X2	1.664854387	6.64E-05	NA	NA	NA	NA
LOC110377047	serine protease 3-like isoform X4	1.664854387	6.64E-05	NA	NA	NA	NA
LOC110377047	serine protease 3-like isoform X1	1.664854387	6.64E-05	NA	NA	NA	NA
LOC110383822	endothelial lipase-like	1.620911727	0.000712158	NA	NA	NA	NA
LOC110384489	brachyurin-like	1.565981186	0.000238418	NA	NA	NA	NA
LOC110376134	trypsin CFT-1-like	1.391100046	1.25E-06	NA	NA	NA	NA
LOC110379949	vitellogenin-like	1.292430297	0.007074947	NA	NA	NA	NA
LOC110384347	ATP-binding cassette sub-family G member 1-like	1.26917551	0.004020078	NA	NA	NA	NA
LOC110380906	fatty acid-binding protein 2-like	1.251556352	0.001467687	NA	NA	NA	NA
LOC110369923	probable serine hydrolase	1.233498139	0.049659453	NA	NA	NA	NA

LOC110382227	hexokinase-2-like isoform X1	1.192743474	0.009278077	NA	NA	NA	NA
LOC110382227	hexokinase-2-like isoform X2	1.192743474	0.009278077	NA	NA	NA	NA
LOC110380200	uncharacterized protein LOC110380200	1.154771763	0.004203356	NA	NA	NA	NA
LOC110371735	4-coumarate--CoA ligase-like 9	1.147344524	0.001854156	NA	NA	NA	NA
LOC110373169	glutathione S-transferase 2-like	1.133581153	0.015044647	NA	NA	NA	NA
LOC110379934	ethanolaminephosphotransferase 1-like	1.08632038	0.011602282	NA	NA	NA	NA
LOC110382890	high affinity copper uptake protein 1-like	1.079165814	0.001274377	NA	NA	NA	NA
LOC110376924	sorbitol dehydrogenase-like	1.072973928	0.005140128	NA	NA	NA	NA
LOC110377790	trans-1,2-dihydrobenzene-1,2-diol dehydrogenase-like	1.006241009	0.043914759	NA	NA	NA	NA
LOC110380370	collagen alpha-1(IV) chain	1.003724708	0.006883417	NA	NA	NA	NA
LOC110381310	translational regulator orb2 isoform X4	-1.000193373	0.000568527	NA	NA	NA	NA
LOC110381310	cytoplasmic polyadenylation element-binding protein 2 isoform X1	-1.000193373	0.000568527	NA	NA	NA	NA
LOC110381310	translational regulator orb2 isoform X2	-1.000193373	0.000568527	NA	NA	NA	NA

LOC110381310	translational regulator orb2 isoform X3	-1.000193373	0.000568527	NA	NA	NA	NA
LOC110380173	uncharacterized protein LOC110380173 isoform X4	-1.007257005	0.000975126	NA	NA	NA	NA
LOC110380173	uncharacterized protein LOC110380173 isoform X2	-1.007257005	0.000975126	NA	NA	NA	NA
LOC110380173	uncharacterized protein LOC110380173 isoform X1	-1.007257005	0.000975126	NA	NA	NA	NA
LOC110380173	uncharacterized protein LOC110380173 isoform X3	-1.007257005	0.000975126	NA	NA	NA	NA
LOC110374687	fasciclin-1 isoform X1	-1.010523814	0.002373765	NA	NA	NA	NA
LOC110374687	fasciclin-1 isoform X2	-1.010523814	0.002373765	NA	NA	NA	NA
LOC110371889	schwannomin-interacting protein 1 homolog isoform X2	-1.017219129	4.78E-05	NA	NA	NA	NA
LOC110371889	schwannomin-interacting protein 1 homolog isoform X1	-1.017219129	4.78E-05	NA	NA	NA	NA
LOC110383036	gelsolin-like isoform X5	-1.021222343	0.048677967	NA	NA	NA	NA
LOC110383036	gelsolin-like isoform X3	-1.021222343	0.048677967	NA	NA	NA	NA
LOC110383036	gelsolin-like isoform X1	-1.021222343	0.048677967	NA	NA	NA	NA
LOC110383036	gelsolin-like isoform X2	-1.021222343	0.048677967	NA	NA	NA	NA
LOC110383036	gelsolin-like isoform X4	-1.021222343	0.048677967	NA	NA	NA	NA

LOC110377255	microtubule-associated protein Jupiter isoform X4	-1.032787405	0.001343764	NA	NA	NA	NA
LOC110377255	microtubule-associated protein Jupiter isoform X1	-1.032787405	0.001343764	NA	NA	NA	NA
LOC110377255	uncharacterized protein LOC110377255 isoform X10	-1.032787405	0.001343764	NA	NA	NA	NA
LOC110377255	microtubule-associated protein Jupiter isoform X5	-1.032787405	0.001343764	NA	NA	NA	NA
LOC110377255	microtubule-associated protein Jupiter isoform X6	-1.032787405	0.001343764	NA	NA	NA	NA
LOC110377255	microtubule-associated protein Jupiter isoform X9	-1.032787405	0.001343764	NA	NA	NA	NA
LOC110377255	microtubule-associated protein Jupiter isoform X2	-1.032787405	0.001343764	NA	NA	NA	NA
LOC110377255	microtubule-associated protein Jupiter isoform X8	-1.032787405	0.001343764	NA	NA	NA	NA
LOC110377255	microtubule-associated protein Jupiter isoform X3	-1.032787405	0.001343764	NA	NA	NA	NA

LOC110377255	microtubule-associated protein Jupiter isoform X7	-1.032787405	0.001343764	NA	NA	NA	NA
LOC110377265	uncharacterized protein LOC110377265	-1.068184388	0.040744839	NA	NA	NA	NA
LOC110379317	myrosinase 1-like	-1.070836209	2.40E-05	NA	NA	NA	NA
LOC110374557	cytochrome P450 6B2-like	-1.072699541	0.001610248	NA	NA	NA	NA
LOC110371445	uncharacterized protein LOC110371445 isoform X1	-1.072851487	0.027964593	NA	NA	NA	NA
LOC110371445	uncharacterized protein LOC110371445 isoform X2	-1.072851487	0.027964593	NA	NA	NA	NA
LOC110382960	SWI5-dependent HO expression protein 3-like	-1.079108058	0.013558052	NA	NA	NA	NA
LOC110382655	LOW QUALITY PROTEIN: neutral ceramidase	-1.095551721	1.04E-05	NA	NA	NA	NA
LOC110381181	carcinine transporter-like isoform X2	-1.103905207	0.008642938	NA	NA	NA	NA
LOC110381181	carcinine transporter-like isoform X1	-1.103905207	0.008642938	NA	NA	NA	NA
LOC110378976	proteoglycan 4-like	-1.108596453	0.000729019	NA	NA	NA	NA
LOC110375344	uncharacterized protein LOC110375344	-1.109044664	0.024096797	NA	NA	NA	NA
LOC110380678	uncharacterized protein LOC110380678	-1.123870399	0.018288317	NA	NA	NA	NA

LOC110370760	phosphoinositide 3-kinase adapter protein 1 isoform X2	-1.128124038	0.012742026	NA	NA	NA	NA
LOC110370760	phosphoinositide 3-kinase adapter protein 1 isoform X3	-1.128124038	0.012742026	NA	NA	NA	NA
LOC110370760	phosphoinositide 3-kinase adapter protein 1 isoform X5	-1.128124038	0.012742026	NA	NA	NA	NA
LOC110370760	phosphoinositide 3-kinase adapter protein 1 isoform X1	-1.128124038	0.012742026	NA	NA	NA	NA
LOC110370760	phosphoinositide 3-kinase adapter protein 1 isoform X4	-1.128124038	0.012742026	NA	NA	NA	NA
LOC110382635	ommochrome-binding protein-like	-1.139807135	0.031948394	NA	NA	NA	NA
LOC110371467	desumoylating isopeptidase 2 isoform X1	-1.1503791	0.000601139	NA	NA	NA	NA
LOC110371467	desumoylating isopeptidase 2 isoform X5	-1.1503791	0.000601139	NA	NA	NA	NA
LOC110371467	desumoylating isopeptidase 2 isoform X3	-1.1503791	0.000601139	NA	NA	NA	NA
LOC110371467	desumoylating isopeptidase 2 isoform X4	-1.1503791	0.000601139	NA	NA	NA	NA

LOC110371467	desumoylating isopeptidase 2 isoform X2	-1.1503791	0.000601139	NA	NA	NA	NA
LOC110370807	apolipoprotein D-like	-1.161226313	0.004277108	NA	NA	NA	NA
LOC110383997	ommochrome-binding protein-like	-1.177826889	0.038628215	NA	NA	NA	NA
LOC110378831	uncharacterized protein LOC110378831 isoform X2	-1.184512253	0.001044393	NA	NA	NA	NA
LOC110378831	uncharacterized protein LOC110378831 isoform X1	-1.184512253	0.001044393	NA	NA	NA	NA
LOC110377315	potassium channel subfamily K member 18-like isoform X1	-1.254449091	0.026093642	NA	NA	NA	NA
LOC110377315	TWiK family of potassium channels protein 7-like isoform X2	-1.254449091	0.026093642	NA	NA	NA	NA
LOC110379703	putative inorganic phosphate cotransporter	-1.266783414	0.010644468	NA	NA	NA	NA
LOC110378426	neuroglobin-like	-1.325499857	0.01387025	NA	NA	NA	NA
LOC110369916	transcription initiation factor TFIID subunit 5-like isoform X2	-1.365358336	0.039391525	NA	NA	NA	NA
LOC110369916	transcription initiation factor TFIID subunit 5-like isoform X1	-1.365358336	0.039391525	NA	NA	NA	NA

LOC110377595	zinc-type alcohol dehydrogenase-like protein C1773.06c	-1.413241894	0.02130042	NA	NA	NA	NA
LOC110377607	lachesin-like isoform X2	-1.419340231	0.003986071	NA	NA	NA	NA
LOC110377607	lachesin-like isoform X1	-1.419340231	0.003986071	NA	NA	NA	NA
LOC110372624	uncharacterized protein LOC110372624	-1.470488041	4.13E-05	NA	NA	NA	NA
LOC110384435	nidogen-1	-1.480282181	5.77E-05	NA	NA	NA	NA
LOC110371228	CD82 antigen-like	-1.513849231	0.010239074	NA	NA	NA	NA
LOC110380721	lachesin-like	-1.531575392	0.045871971	NA	NA	NA	NA
LOC110373813	pancreatic triacylglycerol lipase-like	-1.542865703	0.000899266	NA	NA	NA	NA
LOC110371039	tubulin polyglutamylase complex subunit 2	-1.551065665	0.027575883	NA	NA	NA	NA
LOC110369990	heparan sulfate glucosamine 3-O-sulfotransferase 1	-1.55432489	0.01967722	NA	NA	NA	NA
LOC110382451	ankyrin repeat domain-containing protein 50-like	-1.596801214	0.010276845	NA	NA	NA	NA
LOC110384297	organic cation transporter protein-like	-1.602376793	0.03233705	NA	NA	NA	NA
LOC110374192	uncharacterized protein LOC110374192	-1.6401675	0.000606293	NA	NA	NA	NA
LOC110374315	uncharacterized protein LOC110374315	-1.657486633	0.027384102	NA	NA	NA	NA
LOC110375545	beta-1,3-glucan-binding protein-like	-1.748593784	3.05E-09	NA	NA	NA	NA

LOC110372874	multidrug resistance protein 1B-like	-1.75611272	0.041234159	NA	NA	NA	NA
LOC110374901	uncharacterized protein LOC110374901 isoform X2	-1.770354336	0.021794605	NA	NA	NA	NA
LOC110374901	uncharacterized protein LOC110374901 isoform X1	-1.770354336	0.021794605	NA	NA	NA	NA
LOC110383784	uncharacterized protein LOC110383784	-1.813524749	0.001831296	NA	NA	NA	NA
LOC110375642	angiotensin-converting enzyme-like isoform X1	-1.904468782	0.000709894	NA	NA	NA	NA
LOC110375642	angiotensin-converting enzyme-like isoform X2	-1.904468782	0.000709894	NA	NA	NA	NA
LOC110376969	hydrocephalus-inducing protein homolog	-2.06241517	0.039106464	NA	NA	NA	NA
LOC110373239	NA	-2.122405523	0.028630108	NA	NA	NA	NA
LOC110383364	dynein regulatory complex protein 1	-2.198380242	0.002230591	NA	NA	NA	NA
LOC110381866	uncharacterized protein LOC110381866	-2.268238042	0.047073899	NA	NA	NA	NA
LOC110381918	annexin A7-like	-2.387553544	2.28E-05	NA	NA	NA	NA
LOC110372675	MORN repeat-containing protein 5-like	-2.457126614	0.007801674	NA	NA	NA	NA
LOC110369721	uncharacterized protein LOC110369721	-2.558926796	0.014983167	NA	NA	NA	NA
LOC110370133	uncharacterized protein LOC110370133	-2.802511211	0.01846131	NA	NA	NA	NA

LOC110379032	UPF0193 protein EVG1 homolog isoform X1	-2.915735588	0.009759101	NA	NA	NA	NA
LOC110379032	UPF0193 protein EVG1 isoform X2	-2.915735588	0.009759101	NA	NA	NA	NA
LOC110371006	tenascin-like isoform X2	-2.966598664	0.019082503	NA	NA	NA	NA
LOC110371006	tenascin-like isoform X3	-2.966598664	0.019082503	NA	NA	NA	NA
LOC110371006	tenascin-like isoform X1	-2.966598664	0.019082503	NA	NA	NA	NA
LOC110371008	uncharacterized protein LOC110371008	-3.19170528	0.040567504	NA	NA	NA	NA
LOC110379082	katanin p60 ATPase-containing subunit A-like 2	-3.45273885	0.013549563	NA	NA	NA	NA
LOC110380360	transmembrane protease serine 9-like	-3.506880192	0.014741219	NA	NA	NA	NA
LOC110374749	gustatory receptor 68a isoform X2	-3.641998304	0.022408152	NA	NA	NA	NA
LOC110374749	gustatory receptor 68a isoform X1	-3.641998304	0.022408152	NA	NA	NA	NA
LOC110381076	alpha-tocopherol transfer protein-like	-3.696619321	0.002597251	NA	NA	NA	NA
LOC110380902	luciferin 4-monooxygenase-like	-3.769548852	0.000654017	NA	NA	NA	NA
LOC110377504	uncharacterized protein LOC110377504	-3.78130668	0.038329544	NA	NA	NA	NA
LOC110380450	luciferin 4-monooxygenase-like	-12.46164171	0.04978224	NA	NA	NA	NA

Table S2. Differentially methylated genes in the genome of *H. armigera* larvae exposed to SPI.

Gene	Protein	No. of sites
LOC110378315	ATP synthase subunit alpha, mitochondrial	3
LOC110384175	uncharacterized LOC110384175	3
LOC110370397	probable bifunctional methylenetetrahydrofolate dehydrogenase/cyclohydrolase 2, transcript variant X1	2
LOC110375315	ER membrane protein complex subunit 1, transcript variant X1	2
LOC110379868	RNA-binding protein lark, transcript variant X1	2
LOC110382346	acylglycerol kinase, mitochondrial	2
LOC110369599	zinc finger Y-chromosomal protein 2-like	1
LOC110369894	kinesin heavy chain	1
LOC110369910	serine/threonine-protein phosphatase 2B catalytic subunit 3-like	1
LOC110369926	pre-rRNA processing protein FTSJ3	1
LOC110369936	A-kinase anchor protein 10, mitochondrial	1
LOC110370106	acyl-protein thioesterase 1	1
LOC110370345	maternal protein pumilio, transcript variant X1	1
LOC110370365	46 kDa FK506-binding nuclear protein	1
LOC110370373	ubiquitin carboxyl-terminal hydrolase 3-like	1
LOC110370633	lipoma-preferred partner homolog	1
LOC110370777	serine/threonine-protein phosphatase 4 catalytic subunit, transcript variant X1	1
LOC110370916	cytochrome b-c1 complex subunit 6, mitochondrial-like	1
LOC110370979	tumor suppressor candidate 3	1
LOC110371225	tumor susceptibility gene 101 protein	1
LOC110371715	microprocessor complex subunit DGCR8, transcript variant X1	1
LOC110371742	PHD finger protein 12, transcript variant X1	1
LOC110372656	U3 small nucleolar ribonucleoprotein protein MPP10	1
LOC110372825	THO complex subunit 4	1
LOC110372886	beta-arrestin-1, transcript variant X1	1
LOC110372934	elongation factor 1-alpha, transcript variant X1	1
LOC110373071	39S ribosomal protein L3, mitochondrial	1
LOC110373091	elongation factor Ts, mitochondrial, transcript variant X1	1
LOC110373572	coatomer subunit alpha	1
LOC110373693	mucolipin-3-like	1
LOC110374162	zinc finger SWIM domain-containing protein 8-like	1
LOC110374222	ras-related protein Rab-21	1
LOC110374361	regulator of nonsense transcripts 1 homolog	1
LOC110374522	polypeptide N-acetylgalactosaminyltransferase 35A, transcript variant X1	1
LOC110374523	nuclear pore complex protein Nup153-like	1
LOC110374600	serine/threonine-protein kinase tricorner, transcript variant X1	1
LOC110374608	ankyrin repeat domain-containing protein 11, transcript variant X1	1

LOC110374662	nudC domain-containing protein 1	1
LOC110374702	uncharacterized LOC110374702, transcript variant X1	1
LOC110374741	interferon-inducible double-stranded RNA-dependent protein kinase activator A homolog, transcript variant X1	1
LOC110374778	acetyl-coenzyme A transporter 1	1
LOC110375090	39S ribosomal protein L1, mitochondrial	1
LOC110375095	uncharacterized LOC110375095	1
LOC110375190	uncharacterized LOC110375190	1
LOC110375331	uncharacterized LOC110375331, transcript variant X1	1
LOC110375512	transmembrane protein 64	1
LOC110375794	voltage-dependent anion-selective channel-like	1
LOC110375967	spermatogenesis-associated protein 5	1
LOC110376098	lipoma-preferred partner homolog	1
LOC110376292	cysteine and histidine-rich protein 1 homolog, transcript variant X1	1
LOC110376416	serine/threonine-protein phosphatase 5	1
LOC110376630	uncharacterized LOC110376630, transcript variant X1	1
LOC110377207	hsp90 co-chaperone Cdc37	1
LOC110377217	uncharacterized LOC110377217	1
LOC110377968	folliculin	1
LOC110377970	protein LSM14 homolog B-like, transcript variant X1	1
LOC110377971	15-hydroxyprostaglandin dehydrogenase [NAD(+)]-like	1
LOC110378337	polynucleotide 5'-hydroxyl-kinase NOL9, transcript variant X1	1
LOC110378675	alpha-ketoglutarate-dependent dioxygenase alkB homolog 7, mitochondrial	1
LOC110378917	proteasomal ubiquitin receptor ADRM1, transcript variant X1	1
LOC110379071	BTB/POZ domain-containing protein 2-like	1
LOC110379128	ephrin type-B receptor 2	1
LOC110379255	treacle protein-like, transcript variant X1	1
LOC110379285	ornithine carbamoyltransferase-like	1
LOC110379390	rhythmically expressed gene 2 protein-like	1
LOC110379455	lymphokine-activated killer T-cell-originated protein kinase-like	1
LOC110380153	uncharacterized LOC110380153	1
LOC110380382	apoptosis-inducing factor 1, mitochondrial-like	1
LOC110380385	E3 ubiquitin-protein ligase TRIM33	1
LOC110380387	prolyl 3-hydroxylase sudestada1	1
LOC110380458	transcription elongation factor B polypeptide 3-like	1
LOC110380558	GTP-binding protein Rheb homolog	1
LOC110380563	ran-binding protein 3	1
LOC110380616	SWI/SNF-related matrix-associated actin-dependent regulator of chromatin subfamily B member 1-A, transcript variant X1	1
LOC110380775	UPF0553 protein C9orf64 homolog	1
LOC110381017	nucleolysin TIAR, transcript variant X1	1
LOC110381117	uncharacterized LOC110381117	1
LOC110381188	uncharacterized LOC110381188	1
LOC110381189	m7GpppX diphosphatase	1
LOC110381195	pre-mRNA-splicing factor CWC22 homolog	1

LOC110381293	UMP-CMP kinase	1
LOC110381308	protoporphyrinogen oxidase	1
LOC110381400	transcriptional repressor CTCF-like, transcript variant X1	1
LOC110381584	DNA repair protein XRCC1	1
LOC110382008	zinc finger protein 2-like	1
LOC110382341	transcriptional protein SWT1-like, transcript variant X1	1
LOC110382796	polyribonucleotide nucleotidyltransferase 1, mitochondrial	1
LOC110383013	thioredoxin-related transmembrane protein 2 homolog, transcript variant X1	1
LOC110383163	26S protease regulatory subunit 8	1
LOC110383207	inorganic pyrophosphatase	1
LOC110383246	baculoviral IAP repeat-containing protein 3-like	1
LOC110383296	protein IWS1 homolog	1
LOC110383539	zinc finger protein on ecdysone puffs-like, transcript variant X1	1
LOC110383900	thiamine transporter 2-like	1
LOC110384069	ruvB-like helicase 1	1
LOC110384234	mucin-5AC-like	1
LOC110384235	nuclear cap-binding protein subunit 1	1
LOC110384601	DNA repair protein REV1	1

Table S3. Components of artificial diets tested for rearing *H. armigera*.

**Component**	**Artificial diet**	
	**Control****	**Control + 0.5% SPIs**
Bean	75 g	75 g
Wheat germ	60 g	60 g
Soy protein	30 g	30 g
Casein	30 g	30 g
Brewer´s yeast	37.5 g	37.5 g
Ascorbic acid	3.6 g	3.6 g
Sorbic acid	1.8 g	1.8 g
Methyl parahydroxybenzoate (Nipagin)	3 g	3 g
Tetracycline	113 mg	113 mg
Formaldehyde 37%	3.6 ml	3.6 ml
Agar	23 g	23 g
Vitamin solution*	9 ml	9 ml
Water	1200 ml	1200 ml
Soy peptidase inhibitors (0.5%)	-	7.7 g

*Vitamin solution constituents were: 1 g niacinamide, 1 g calcium pantothenate, 0.5 g thiamine, 0.25 g pyridoxine, 0.1 g folic acid, 0.02 g biotin, 1000 (mg/cc) b12 vitamin.
** Mihsfeldt and Parra (1999) [56].

Table S4. Primers for DNA methylation analysis of CpG islands of serine peptidase genes, using the BSP method.

Locus		Primers used in BSP method
LOC110384510	*F*	TTTTTAGGTTTAAAGATTAAATAATTTATT
LOC110384510	*R*	CAAATAACTCACCAAAAATAACAAC
LOC110376134	*F*	TTTTTAATTTATATAATGGTGGTGGTG
LOC110376134	*R*	AATCATAAATAAACCTTTATCAATTCTC
LOC110378565	*F*	AAATTAGGGTTTTGAGTAGTTG
LOC110378565	*R*	TTAAAAAAAATCCAAAATATATAAC

Table S5. Primers used for RT-qPCR analysis.

**Locus**		**Primer**
LOC110380583	*F*	5′-TCGGTCTACTAGCCCTCTGC-3′
Trypsin	*R*	5′-CAGCGATGGAGGGGTATTGG-3′
LOC110378559	*F*	5′-CCTCAACAACCGAGCTTTGC-3′
Trypsin	*R*	5′-CACTGTTAGCCCAGGTGGAG-3′
LOC110380575	*F*	5′-CGCAATCGTACGTTCCGCTA-3′
Trypsin	*R*	5′-GGAAGAGGTTGTGCCCCATC-3′
LOC110378559	*F*	5′-CCTCAACAACCGAGCTTTGC-3′
Trypsin	*R*	5′-CACTGTTAGCCCAGGTGGAG-3′
LOC110379155	*F*	5′-GTGTGGAAAATTCACCCGCC-3′
Trypsin	*R*	5′-GCCACCAACAATCCTTGCAG-3′
LOC110384480	*F*	5′-CTGGTTTCGGTCTGGCCAAT-3′
Chymotrypsin	*R*	5′-GGTGATCACGGGCAAGTTGA-3′
LOC110384481	*F*	5′-GTGACGGTGGAAGTGTCGAT-3′
Chymotrypsin	*R*	5′-GCAGATGTTGGATGCCTGGA-3′
LOC110384483	*F*	5′-CCAGGGATGGTGGAAGTGTC-3′
Chymotrypsin	*R*	5′-GCCGCTGGTGCAAATGTTAG-3′
LOC110384484	*F*	5′-CGGTGGCACCAGACTTTTCA-3′
Chymotrypsin	*R*	5′-GGGCGATGTTGCCAGAAGTA-3′
DNMT1*	*F*	5′-ATTCACTGGGACGAATCTGC-3′
	R	5′-TTTCGTCTTTGCTCCTGACC-3′
DNMT2*	*F*	5′-TCTTTTGGCGTGCCTAACTC-3′
	R	5′-GGCAATCCATAATCCTTAGGC-3′
LOC110375492	*F*	5′-TGTCAGTAGTCACAGAAGACGC-3′
Acetyltransferase-like protein	*R*	5′-CCATCATGGAAGCCCCAACT-3′
LOC110382234	*F*	5′-GGGATCACAAAAACCATAAAGTCG-3′
Nuclear factor NF-kappa-B p100 subunit-like	*R*	5′-GCCGGTACATACTGCCAAGT-3′
LOC110377935	*F*	5′-AAAGTGCTTTTCCTGCGAAAT-3′
Elongation factor 1-alpha 2	*R*	5′-CATCCTTGTGGCGCGTTATG-3′

*=The specific primers to DNMT1 and DNMT2 were used as described by Baradaran et al. (2019) [73].
